# Cabozantinib and nivolumab with or without live bacterial supplementation in metastatic renal cell carcinoma: a randomized phase 1 trial

**DOI:** 10.1038/s41591-024-03086-4

**Published:** 2024-06-28

**Authors:** Hedyeh Ebrahimi, Nazli Dizman, Luis Meza, Jasnoor Malhotra, Xiaochen Li, Tanya Dorff, Paul Frankel, Marian Llamas-Quitiquit, Joann Hsu, Zeynep B. Zengin, Marice Alcantara, Daniela Castro, Benjamin Mercier, Neal Chawla, Alex Chehrazi-Raffle, Regina Barragan-Carrillo, Salvador Jaime-Casas, Ameish Govindarajan, John Gillece, Jeffrey Trent, Peter P. Lee, Thomas P. Parks, Motomichi Takahashi, Atsushi Hayashi, Marcin Kortylewski, J. Gregory Caporaso, Keehoon Lee, Abhishek Tripathi, Sumanta K. Pal

**Affiliations:** 1 Department of Medical Oncology, City of Hope Comprehensive Cancer Center, Duarte, CA USA; 2 MD Anderson Cancer Center, Houston, TX USA; 3 Yale University School of Medicine, New Haven, CT USA; 4 Department of Biostatistics, City of Hope Comprehensive Cancer Center, Duarte, CA USA; 5 Department of Immuno-Oncology, Beckman Research Institute, City of Hope Comprehensive Cancer Center, Duarte, CA USA; 6 Translational Genomics Research Institute (TGen), Phoenix, AZ USA; 7 Osel, Inc., Mountain View, CA USA; 8 Miyarisan Pharmaceutical Co., Ltd, Tokyo, Japan; 9 Translational Genomics Research Institute (TGen), Flagstaff, AZ USA

**Keywords:** Metagenomics, Cancer immunotherapy, Renal cell carcinoma

## Abstract

Supplementation with CBM588, a bifidogenic live bacterial product, has been associated with improved clinical outcomes in persons with metastatic renal cell carcinoma (mRCC) receiving nivolumab and ipilimumab. However, its effect on those receiving tyrosine kinase inhibitor-based combinations is unknown. In this open-label, randomized, investigator-initiated, phase 1 study, 30 participants with locally advanced or mRCC with histological confirmation of clear cell, papillary or sarcomatoid component were randomized in a 2:1 fashion to receive cabozantinib (an inhibitor of vascular endothelial growth factor receptor, MET and AXL) and nivolumab (anti-programmed cell death protein 1) with or without CBM588 as first-line treatment. Metagenomic sequencing was performed on stool samples to characterize their gut microbiome at baseline and 13 weeks into treatment. The primary endpoint was a change in the relative abundance of *Bifidobacterium* spp.; secondary endpoints included objective response rate (ORR), progression-free survival (PFS) and toxicity profile. The primary endpoint of the study was not met and the addition of CBM588 to cabozantinib and nivolumab did not result in a difference in the relative abundance of *Bifidobacterium* spp. or alpha diversity (as measured by the Shannon index). However, ORR was significantly higher in participants treated with CBM588 compared to those in the control arm (14 of 19, 74% versus 2 of 10, 20%; *P* = 0.01). PFS at 6 months was 84% (16 of 19) and 60% (6 of 10) in the experimental and control arms, respectively. No significant difference in toxicity profile was seen between the study arms. Our results provide a preliminary signal of improved clinical activity with CBM588 in treatment-naive participants with mRCC receiving cabozantinib and nivolumab. Further investigation is needed to confirm these findings and better characterize the underlying mechanism driving this effect.

ClinicalTrials.gov identifier: NCT05122546

## Abstract (web summary)

In a randomized phase 1 trial, the addition of a live *Clostridium* species-containing product to a tyrosine kinase inhibitor and anti-programmed cell death protein 1 treatment combination did not increase bacterial abundance of *Bifidobacterium* spp. but enhanced clinical responses in participants with metastatic renal cell carcinoma.

## Main

Outcomes for metastatic renal cell carcinoma (mRCC) have improved markedly with the advent of immune checkpoint inhibitors (ICIs)^1^. Approved ICIs for mRCC promote antitumor activity through blockade of programmed cell death ligand 1 (PDL1), its cognate receptor programmed cell death protein 1 (PD1) or cytotoxic T lymphocyte-associated protein 4 (CTLA4)^2^. Current guidelines recommend that persons with newly diagnosed mRCC receive either a combination of nivolumab with ipilimumab (PD1 and CTLA4 inhibitors, respectively) or a vascular endothelial growth factor receptor tyrosine kinase inhibitor (VEGFR-TKI) with a PD1 inhibitor, based on the improved overall survival (OS) seen in multiple recent randomized clinical trials^3^. The most commonly used VEGFR-TKI + PD1 inhibitor combinations include cabozantinib + nivolumab, lenvatinib + pembrolizumab and axitinib + pembrolizumab, all supported by randomized phase 3 clinical trials showing a survival benefit over VEGFR-TKI monotherapy^4–6^.

Although a modest proportion of persons (10–17%) will achieve a complete response (CR) to these therapies, the vast majority will ultimately experience disease progression on treatment^4–7^. Subsequent lines of salvage therapy for mRCC remain largely palliative with limited durability of responses^8–10^. In an effort to improve front-line therapy, further treatment intensification with triplet regimens has been proposed, such as combining VEGFR-TKI therapy with CTLA4 and PD1 inhibition. To date, only one phase 3 trial comparing triplet and doublet therapy has been completed^11^. However, although there was a signal of activity with triplet therapy, the regimen was marred by notable toxicity concerns.

An alternative approach to build on the currently approved doublets could be to combine them with strategies with novel mechanisms of action and nonoverlapping toxicity. Microbiome modulation represents one such approach. To date, multiple studies spanning lung cancer, melanoma and mRCC, among others, have shown that the composition of the gut microbiome can potentially predict outcomes with immunotherapy^12–14^. The first suggestion that microbiome modulation could augment ICI activity was derived from studies assessing fecal microbiome transplant^15,16^. Although this approach is promising, there are undoubtedly challenges related to safety, acceptance among patients and scalability for widespread clinical use. Another approach to microbiome modulation is through the administration of prebiotics, probiotics or live bacterial products (LBPs). CBM588 belongs to the latter category and is a strain of *Clostridium butyricum* that is widely used in Japan for a variety of gastrointestinal disorders. In preclinical models, CBM588 demonstrated butyrogenic properties that foster the growth of *Bifidobacterium* spp. We postulated that these changes could be associated with improved ICI response^17,18^. To examine this clinically, our group previously conducted and reported the results of a pilot trial assessing nivolumab + ipilimumab with or without CBM588 in participants with mRCC, suggesting a significant improvement in progression-free survival (PFS) and objective response rate (ORR) with the addition of the LBP^19^. To explore whether CBM588 might complement not only dual ICI therapy but also VEGFR-TKI + PD1 combinations, we undertook the current study evaluating its effect on the gut microbiome composition when administered in combination with cabozantinib + nivolumab as front-line therapy for locally advanced or mRCC.

## Results

### Trial design and participant characteristics

We conducted a single-center, randomized, open-label, investigator-initiated phase 1 study to evaluate the effects of CBM588 on the gut microbiome composition when administered in combination with cabozantinib + nivolumab in persons with advanced or mRCC. This trial enrolled persons with histologically confirmed advanced or mRCC with a clear cell, papillary or sarcomatoid component who did not receive prior systemic therapy for mRCC and had a Karnofsky performance status ≥ 70%. The primary endpoint was to determine the change in *Bifidobacterium* spp. composition of stool from baseline to week 13 of treatment. Secondary endpoints included comparing the Shannon index (a measure of microbial alpha diversity) from baseline to week 13 of therapy, clinical efficacy measures such as best ORR and PFS, safety and changes in circulating cytokines and immune cell populations.

**Fig. 1:**
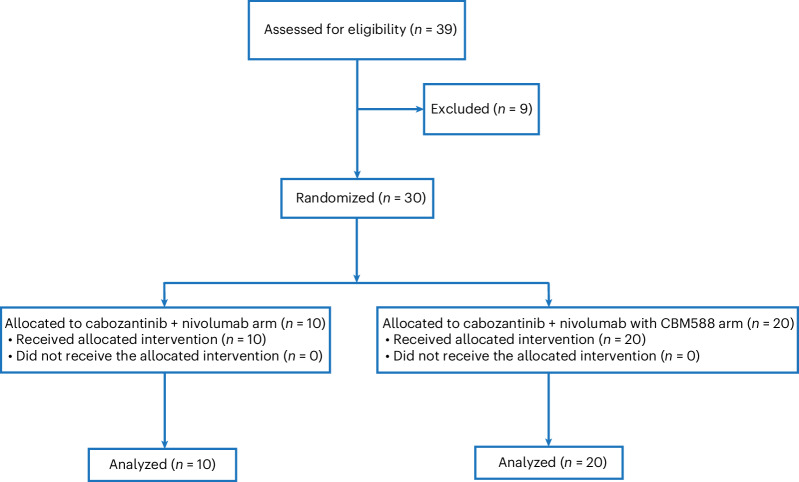
CONSORT (Consolidated Standards of Reporting Trials) diagram. CONSORT diagram showing the flow of participant enrollment and treatment.

A total of 30 participants with locally advanced or mRCC were randomized and treated between November 3, 2021 and March 6, 2023 to receive the combination of cabozantinib and nivolumab with or without CBM588 (Fig. 1). Baseline characteristics were comparable between arms and are summarized in Table 1. The median age in the overall cohort at the time of treatment initiation was 65 years (range, 36–84 years). The majority of participants were male (67%) and had intermediate-risk or poor-risk disease (60%), as defined by the International mRCC Database Consortium (IMDC). While clear cell RCC comprised the majority of participants (87%), five participants (17%) had sarcomatoid features or dedifferentiation and two participants had papillary RCC. The most common sites of metastasis at the time of enrollment were lung (80%), lymph nodes (50%) and bone (40%).

**Table 1:** Participant characteristics

	Overall (*n* = 30), median (range) or *n* (%)	Cabozantinib + nivolumab (*n* = 10), median (range) or *n* (%)	Cabozantinib + nivolumab + CBM588 (*n* = 20), median (range) or *n* (%)	*P* value
Age (years)	65 (36–84)	60 (48–67)	68 (36–84)	0.237
Gender				
Male	20 (67)	5 (50)	15 (75)	0.230
Female	10 (33)	5 (50)	5 (25)	
Race				
White	26 (87)	7 (70)	19 (95)	0.563
Asian	3 (10)	2 (20)	1 (5)	
Other	1 (3)	1 (10)	0 (0)	
Ethnicity				
Non-Hispanic or non-Latinx	15 (50)	4 (40)	11 (55)	0.699
Hispanic or Latinx	15 (50)	6 (60)	9 (45)	
Histologic subtype				
Clear cell	26 (87)	8 (80)	18 (90)	0.584
Clear cell with sarcomatoid features	3 (10)	1 (10)	2 (10)	
Papillary	2 (7)	0 (0)	2 (10)	
Sarcomatoid dedifferentiation	2 (7)	2 (20)	0 (0)	
IMDC prognostic risk				
Favorable	12 (40)	3 (30)	9 (45)	0.070
Intermediate	12 (40)	5 (50)	7 (35)	
Poor	6 (20)	2 (20)	4 (20)	
Nephrectomy				
Yes	20 (67)	6 (60)	14 (70)	0.690
No	10 (33)	4 (40)	6 (30)	
Number of metastatic sites				
≥2	24 (80)	8 (80)	16 (80)	1.000
Most common metastatic sites				
Lung	24 (80)	8 (80)	16 (80)	1.000
Lymph node	15 (50)	6 (60)	9 (45)	0.699
Bone	12 (40)	4 (40)	8 (40)	1.000
Adrenal	5 (17)	0 (0)	5 (25)	0.140
Liver	3 (10)	2 (20)	1 (5)	0.251
Pancreas	1 (3)	1 (10)	0 (0)	0.333

### Microbiome assessment

**Fig. 2:**
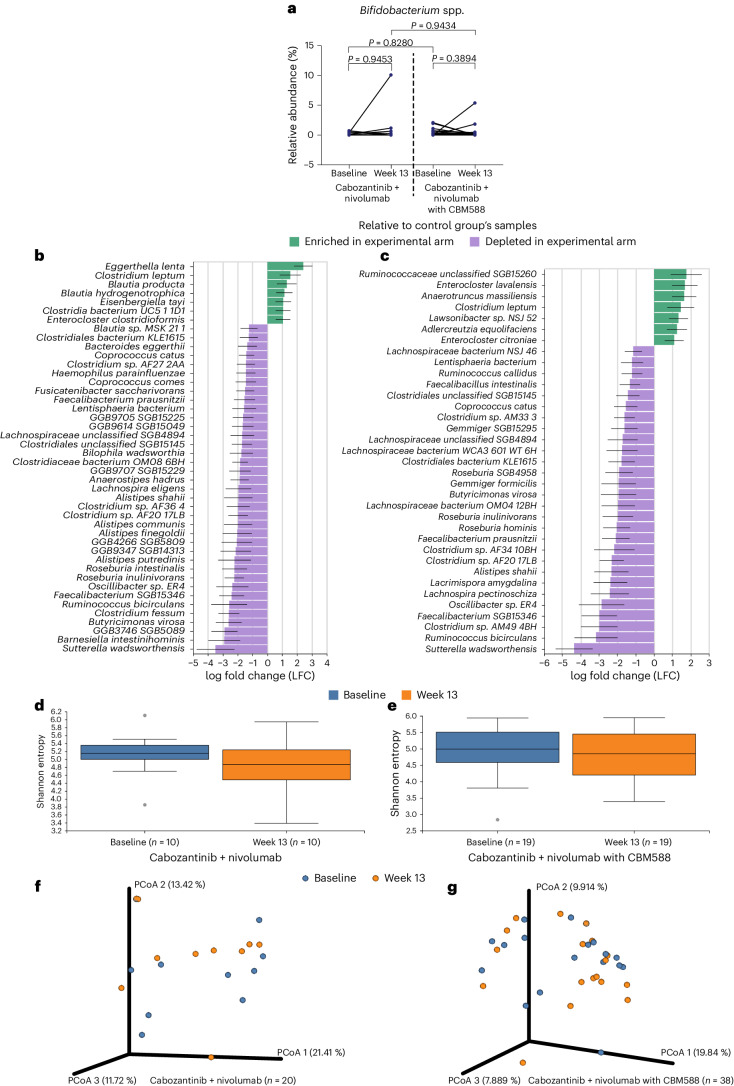
Microbiome assessment in participants with mRCC treated with cabozantinib + nivolumab with or without CBM588 revealed no significant changes in *Bifidobacterium* spp. with treatment. Analyses were performed using *n* = 58 stool samples from *n* = 29 participants (*n* = 10 participants in the cabozantinib + nivolumab arm and *n* = 19 participants in the cabozantinib + nivolumab with CBM588 arm). **a**, Change in relative abundance of *Bifidobacterium* spp. from baseline to week 13 in participants by treatment arm. A two-sided Wilcoxon signed rank test was used to perform comparisons between two time points within the same treatment arm and a two-sided Mann–Whitney *U* test was used for comparisons between the two arms. **b**, Difference in the relative abundance of several bacterial species in baseline samples from participants receiving CBM588 (*n* = 19) compared to those in the control arm (*n* = 10). The ANCOM-BC was used to perform comparisons in the CBM588 arm relative to the control arm at baseline. Data are presented by effect size depicting features with LFC > 1 and *P* < 0.05 (per two-sided *z*-test using the Wilcoxon test statistics). Error bars represent the effect size error (SE). **c**, Difference in the relative abundance of several bacterial species in week 13 samples from participants receiving CBM588 (*n* = 19) compared to those in the control arm (*n* = 10). ANCOM-BC was used to perform comparisons in the CBM588 arm relative to the control arm at week 13. Data are presented by effect size depicting features with LFC > 1 and *P* < 0.05 (per two-sided *z*-test using the Wilcoxon test statistics). Error bars represent the SE. **d**, Microbial richness between baseline and week 13 in participants with the cabozantinib + nivolumab treatment. The Shannon entropy diversity metric was used to compare two time points. The median and interquartile range are depicted, with whiskers extending to the minimum and maximum values. **e**, Microbial richness between baseline and week 13 in participants with the cabozantinib + nivolumab with CBM588 treatment. The Shannon entropy diversity metric was used to compare two time points. The median and interquartile range are depicted, with whiskers extending to the minimum and maximum values. **f**, Relative similarities of microbiome composition as a principal coordinate analysis (PCoA) of Bray–Curtis distances between control samples. **g**, Relative similarities of microbiome composition as a PCoA of Bray–Curtis distances between samples of participants receiving CBM588.

**Extended Data Fig. 1:**
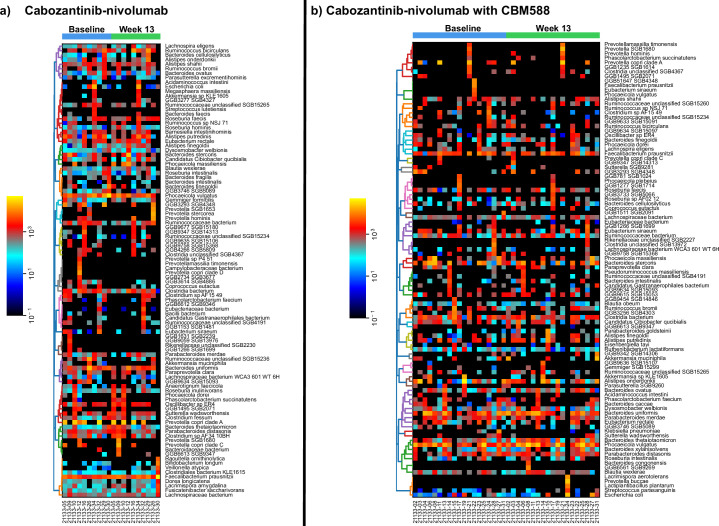
Differentially abundant bacterial species in stool microbiome. Heatmap of differentially abundant bacterial species in stool microbiome across patients in (**a**) control arm (n = 10) and (**b**) experimental arm (n = 19) of the study at baseline and week 13 (top 100 most abundant bacterial species are presented).

**Extended Data Fig. 2:**
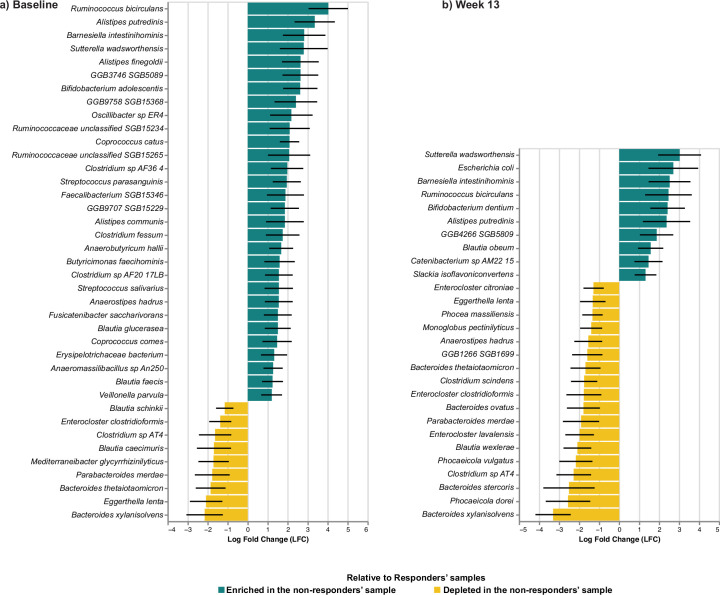
Differentially abundant microbial feature assessment in patients with or without objective response. (**a**) Difference in the relative abundances of several bacterial species in baseline samples of patients without objective response (n = 13) compared to patients with response (n = 16). The ANCOM-BC (Analysis of Composition of Microbiomes with Bias Correction) was used to determine the differentiating features in patients without response relative to those with response at baseline (differential features with log fold change (LFC) greater than 1 and p < 0.05 are indicated). The P value was calculated through two-sided z-test using the W test statistics. Error bars represent effect size error (SE). (**b**) Difference in the relative abundances of several bacterial species in week 13 samples of patients without objective response (n = 13) compared to patients with response (n = 16). The ANCOM-BC was used to determine the differentiating features in patients without response relative to those with response at week 13 (differential features with LFC greater than 1 and p < 0.05 are indicated). The P value was calculated through two-sided z-test using the W test statistics. Error bars represent effect size error (SE).

Baseline and week 13 stool samples were collected for all participants except for one participant randomized to the intervention arm, who withdrew from the study before the collection of the second stool sample. No significant difference in the relative abundance of *Bifidobacterium* spp. was found between baseline and week 13 samples for either treatment arms using the Wilcoxon matched-pairs test (*P* = 0.95 and *P* = 0.39 for the control and experimental arms, respectively; Fig. 2a). Using ANCOM-BC (analysis of composition of microbiomes with bias correction), we identified that, at week 13, there was an enrichment of Ruminococcaceae unclassified SGB15260 in the experimental arm compared to the control arm (log fold change (LFC) = 1.76, *P* = 0.03 and *q* = 1; Fig. 2b,c). When examining the stool’s alpha bacterial diversity, no statistically significant difference based on time of collection was observed with cabozantinib + nivolumab alone or with CBM588 (*P* = 0.17 and *P* = 0.65, respectively; Fig. 2d,e). Using Bray–Curtis and Jaccard dissimilarity analysis as a measure of beta diversity, no statistically significant difference in taxonomic relative abundance and presence of the features was observed between baseline and week 13 stool samples in the control and experimental arms (*P* = 0.97 and *P* = 0.99, respectively; Fig. 2f,g). A summary of differentially abundant bacterial species in the stool microbiome across participants in each arm of the study at baseline and week 13 and a comparison of differentially abundant bacterial species in participants with or without objective response at baseline and week 13 are provided in Extended Data Figs. 1 and 2, respectively.

**Fig. 3:**
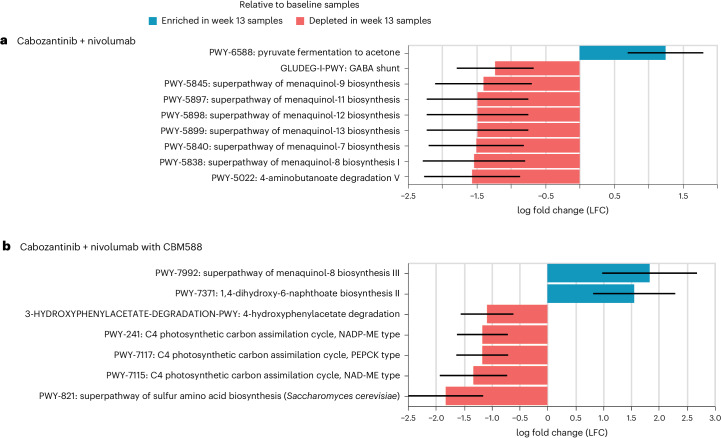
Differentially abundant microbial metabolic pathways in participants with mRCC treated with cabozantinib + nivolumab with or without CBM588. **a**, Differentially abundant microbial metabolic pathways between baseline and week 13 in participants with the cabozantinib + nivolumab treatment (*n* = 10). **b**, Differentially abundant microbial metabolic pathways between baseline and week 13 in participants with the cabozantinib + nivolumab with CBM588 treatment (*n* = 19). ANCOM-BC was used to perform comparisons between two time points within the same treatment arm (differential features with LFC > 1 and *P* < 0.05 are indicated). The *P* value was calculated through a two-sided *z*-test using the Wilcoxon test statistics. Error bars represent the SE.

ANCOM-BC analysis also yielded detailed information regarding differences in the functional metabolic pathways expressed at baseline and week 13. As shown in Fig. 3, a total of seven and nine functional metabolic pathways were found to be differentially expressed after treatment in the experimental and control arms, respectively (with LFC > 1 and *P* < 0.05). Among these, samples from participants receiving cabozantinib + nivolumab plus CBM588 showed relative enrichment of the superpathways of menaquinol-8 biosynthesis III (LFC = 1.83, *P* = 0.03 and *q* = 1) and 1,4-dihydroxy-6-naphthoate biosynthesis II (LFC = 1.55, *P* = 0.03 and *q* = 1), while also showing relative depletion in the superpathways of sulfur amino acid biosynthesis (LFC = −1.82, *P* = 0.006 and *q* = 1) and 3-hydroxyphenylacetate degradation (LFC = −1.08, *P* = 0.02 and *q* = 1). In the control arm, enrichment of the pathway of pyruvate fermentation to acetone (LFC = 1.25, *P* = 0.02 and *q* = 1) and depletion of six superpathways of menaquinol biosynthesis and GABA shunt (LFC = −1.23, *P* = 0.02 and *q* = 1) and 4-aminobutanoate degradation V (LFC = −1.56, *P* = 0.02 and *q* = 1) were observed after treatment.

### Efficacy outcomes

**Fig. 4:**
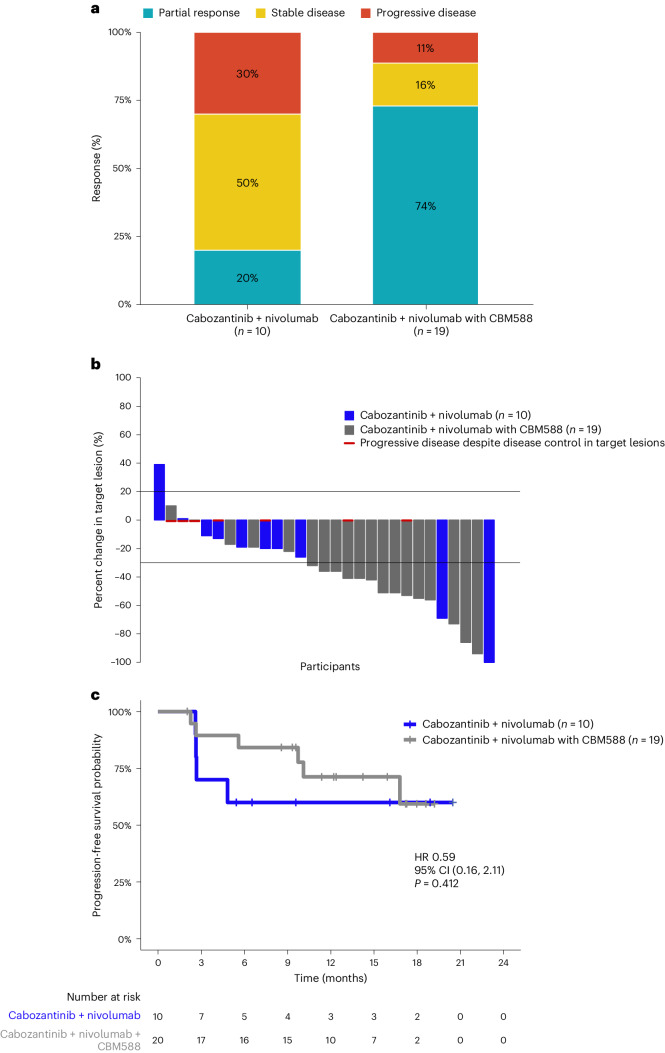
Clinical outcomes in participants with mRCC treated with cabozantinib + nivolumab with or without CBM588. **a**, ORR. **b**, Best change in target lesions. **c**, PFS. The data are from *n* = 29 participants (19 participants in the cabozantinib + nivolumab with CBM588 arm and 10 participants in the cabozantinib + nivolumab arm). The Kaplan–Meier log-rank test was used to compare survival between the two arms.

At the time of data cutoff (August 16, 2023), 18 participants were still on treatment with a median follow-up of 15.9 months (interquartile range, 9.6–18.0). One participant in the cabozantinib + nivolumab + CBM588 arm withdrew from the study before the first objective response assessment. ORR was significantly higher among participants treated with cabozantinib + nivolumab + CBM588 compared to those in the control arm (74% (14 of 19) versus 20% (2 of 10), *P* = 0.01; Fig. 4a). A total of 17 (89%) participants in the intervention arm and eight (80%) in the control arm experienced a reduction in target lesion size. The median decrease in target lesions was 42% (range, 17–94%) in the CBM588 arm compared to 20% (range, 11–100%) in the control arm (Fig. 4b). Additionally, clinical benefit, defined as CR, partial response (PR) or stable disease (SD) for at least 6 months, was achieved in 16 of 20 (80%) participants treated with CBM588 and 6 of 10 (60%) participants not receiving this LBP. Median follow-up was 14.2 and 16.1 months in the experimental and control arms, respectively. The median OS and PFS were not reached in either of the arms at the time of data cutoff; however, landmark PFS at 6 months was 84% and 60% in the experimental and control arms, respectively (Fig. 4c). Extended Data Table 1 provides a summary of participants’ response characteristics by study arm and IMDC prognostic risk.

### Safety

The prevalence of grade 3 or 4 adverse events attributable to treatment was similar across the intervention and control arms (40% each). The most common grade ≥3 toxicities observed in the overall cohort were transaminitis (10%), hypertension (7%) and diarrhea (7%), with no significant differences being observed between treatment arms. A summary of grade ≥2 adverse events is provided in Table 2 and Supplementary Table 1 provides a full list of all recorded adverse events. No treatment-related deaths were observed. Four participants (13%), three in the CBM588-containing arm and one in the control arm, required discontinuation of nivolumab because of adverse events of any cause.

**Table 2:** Grade ≥2 adverse events of treated participants

	Cabozantinib + nivolumab (*n* = 10), *n* (%)			Cabozantinib + nivolumab + CBM588 (*n* = 20), *n* (%)		
	Grade2	Grade 3	Grade 4	Grade2	Grade 3	Grade 4
Overall	4 (40)	3 (30)	1 (10)	5 (25)	8 (40)	0 (0)
Hyponatremia			1 (10)	1 (5)		
Transaminitis		2 (20)		2 (10)	1 (5)	
Hypertension	6 (60)	1 (10)		7(35)	1 (5)	
Diarrhea				1 (5)	2 (10)	
Palmar-plantar erythrodysesthesia syndrome	1 (10)			2 (10)	1 (5)	
White blood cell count drop	1 (10)	1 (10)		2 (10)		
Hypocalcemia	1 (10)	1 (10)		1 (5)		
Arthralgia					1 (5)	
Bullous dermatitis				1 (5)	1 (5)	
Cough					1 (5)	
Pneumonitis					1 (5)	
Vomiting		1 (10)				
Hypoalbuminemia	1 (10)			1 (5)		
Anemia				1 (5)		
Hemorrhoids				1 (5)		
Hyperkalemia				1 (5)		
Hypermagnesemia					1 (5)	
Hypokalemia	1 (10)					
Hypothyroidism				2 (10)		
Lipase elevation	1 (10)					
Sore throat				1 (5)		
Upper gastrointestinal hemorrhage		1 (10)				
Weight loss	1 (10)			1 (5)		

### Circulating cytokines and immune cell populations

**Extended Data Fig. 3:**
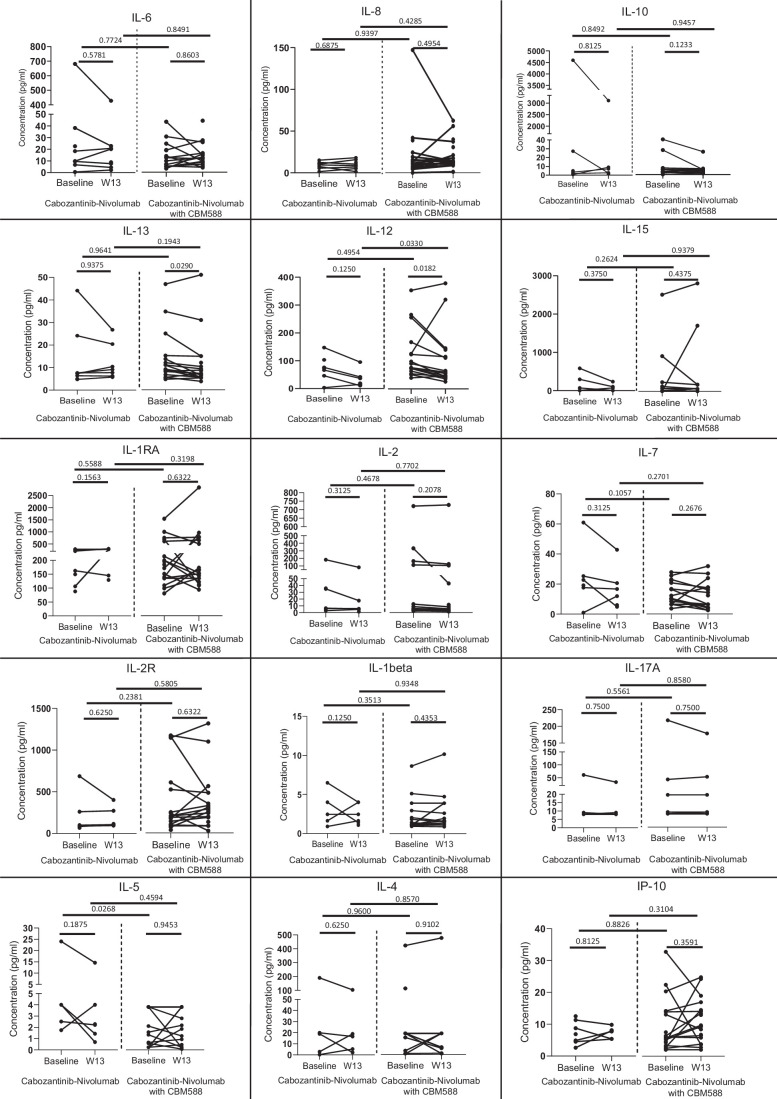
Changes in levels of circulating cytokines from baseline to week 13 by treatment arm – Part 1. A total of 53 samples from 30 patients were available for the final analysis. Two-sided Wilcoxon matched-pairs test was used to compare the levels of cytokines at the two prespecified timepoints. Two-sided Mann–Whitney U test was used for comparisons between the two arms.

**Extended Data Fig. 4:**
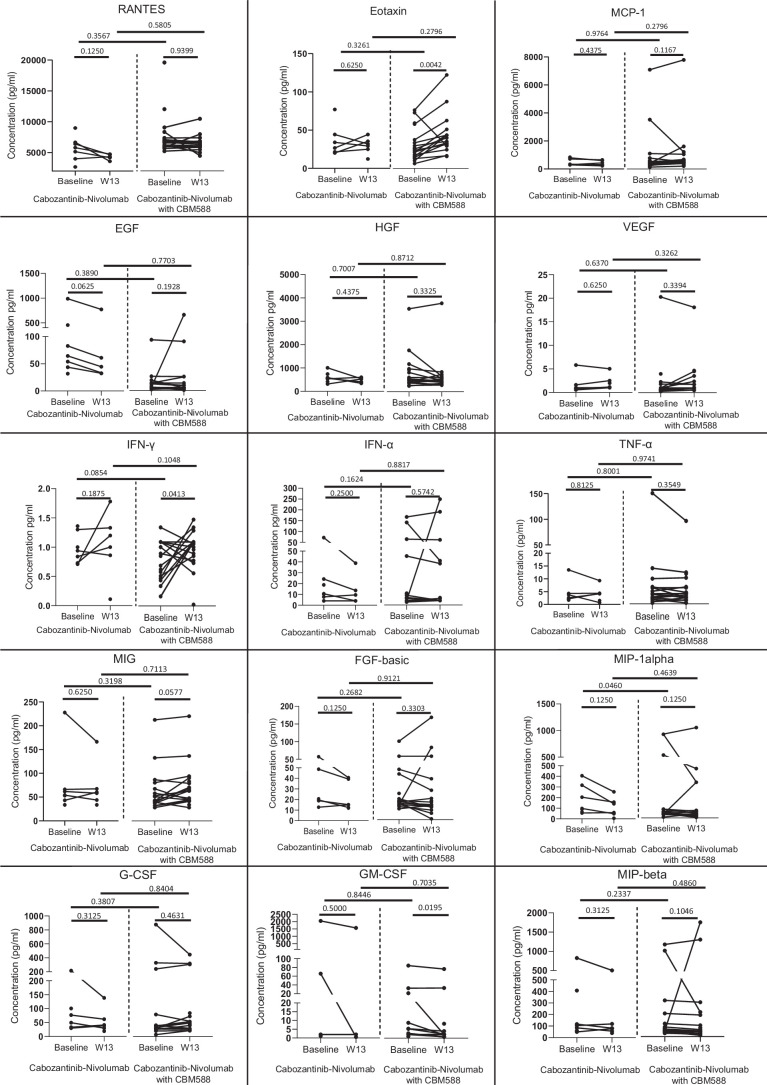
Changes in levels of circulating cytokines from baseline to week 13 by treatment arm – Part 2. A total of 53 samples from 30 patients were available for the final analysis. Two-sided Wilcoxon matched-pairs test was used to compare the levels of cytokines at the two prespecified timepoints. Two-sided Mann–Whitney U test was used for comparisons between the two arms.

**Extended Data Fig. 5:**
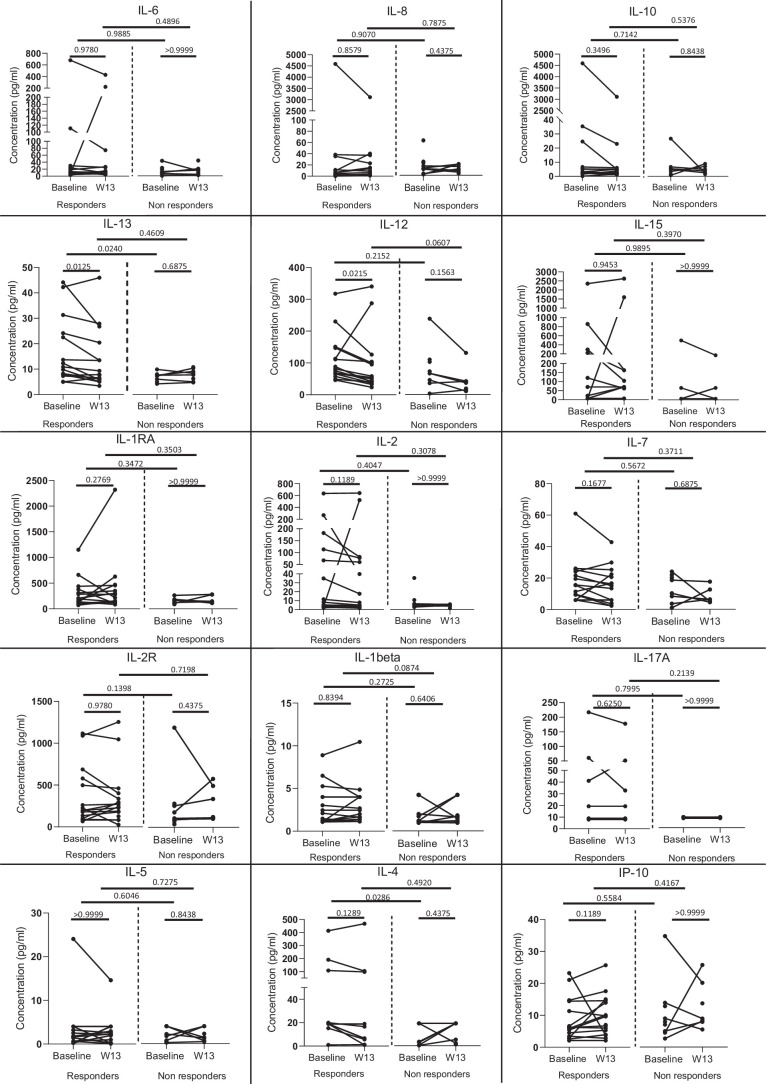
Assessment of circulating cytokine levels in patients with or without response – Part 1. Changes in circulating cytokine levels from baseline to week 13 in patients with or without response were assessed using the two-sided Wilcoxon matched-pairs test. Comparison of circulating cytokine levels at baseline and week 13 between patients with or without response was performed using the two-sided Mann-Whitney U test.

**Extended Data Fig. 6:**
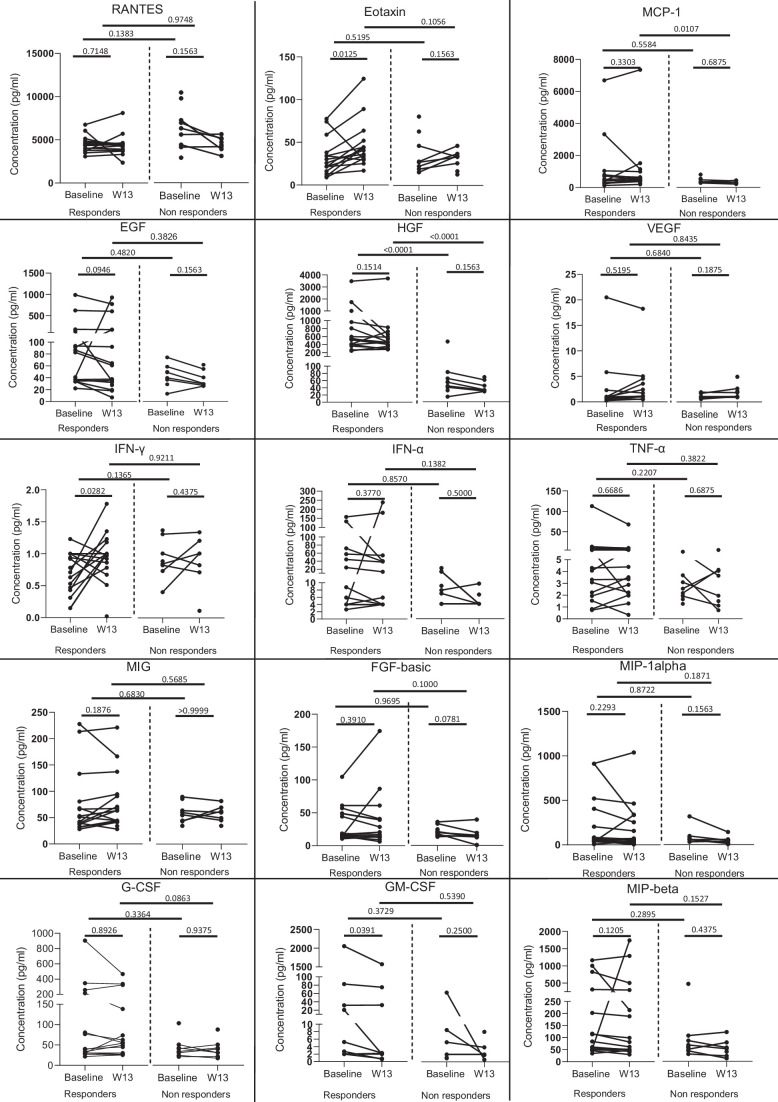
Assessment of circulating cytokine levels in patients with or without response – Part 2. Changes in circulating cytokine levels from baseline to week 13 in patients with or without response were assessed using the two-sided Wilcoxon matched-pairs test. Comparison of circulating cytokine levels at baseline and week 13 between patients with or without response was performed using the two-sided Mann-Whitney U test.

**Extended Data Fig. 7:**
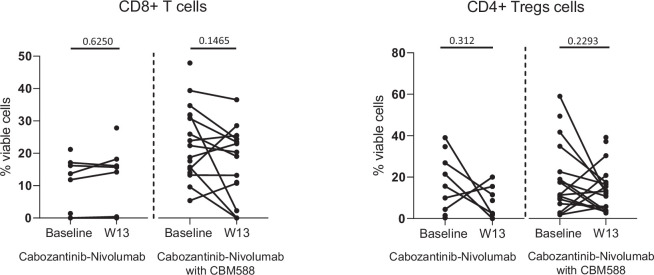
Changes in circulating immune cell populations between baseline and week 13 by treatment arm. Changes in the populations of **(a)** CD8+ T cells and **(b)** CD4+ regulatory T cells from baseline to week 13 in cabozantinib-nivolumab arm or cabozantinib-nivolumab with CBM588 arm. Two-sided Wilcoxon matched-pairs test was used to compare the immune cell populations between the two prespecified timepoints.

Peripheral blood samples were collected at baseline and weeks 9, 13, 17 and 25 of treatment. As week 13 (±7 days) was the expected time for the first response assessment, we chose baseline and week 13 (±7 days) samples for cytokine analysis. A total of 53 samples from 30 participants had the required quality and were available for the final analysis and 30 different cytokines were evaluated. Changes in circulating cytokine levels by treatment arm between baseline and week 13 and a comparison of cytokine levels between arms at baseline and week 13 are shown in Extended Data Figs. 3 and 4. There was a significant difference in the levels of interleukin (IL)-12, IL-13, eotaxin, granulocyte-macrophage colony-stimulating factor (GM-CSF) and interferon-γ (IFNγ) at baseline compared to week 13 in participants who received cabozantinib + nivolumab + CBM588. No other significant changes in cytokine levels were observed between baseline and week 13 samples in either of the study arms. No significant difference in cytokine levels was noted between the control and experimental arms at week 13, except in the level of IL-12. An assessment of changes in cytokine levels between baseline and week 13 in participants with or without response also revealed a significant change in the levels of IL-12, IL-13, eotaxin, IFNγ and GM-CSF in participants who had an objective response (Extended Data Figs. 5 and 6). No significant changes were observed in CD8^+^ T cell and CD4^+^ regulatory T cell populations from baseline to week 13 in the cabozantinib + nivolumab arm or cabozantinib + nivolumab with CBM588 arm (Extended Data Fig. 7).

## Discussion

Our study demonstrates that an LBP may complement the clinical efficacy of combined VEGFR-TKI + PD1 inhibition in persons with mRCC. Although limited by the sample size, the results bolster findings from our previous trial, which showed a similar improvement in clinical efficacy with the addition of CBM588 to nivolumab + ipilimumab^19^. Although no increase in *Bifidobacterium* spp. was observed with CBM588 therapy in the current study, we observed an increase in the abundance of unclassified Ruminococcaceae genera, which were associated with improved clinical outcomes with ICIs in several other recent publications, providing a mechanistic rationale for our results^14,20^.

The first published report to demonstrate the benefit of CBM588 in the context of ICI therapy was a retrospective experience in non-small cell lung cancer (NSCLC)^21^. In this series of 118 participants, 39 participants (33%) received CBM588 before and/or during ICI therapy. These participants were confirmed to have prolonged PFS on both univariate and multivariate analyses. A substantial proportion of participants (39%) had received antibiotic therapy ahead of ICI treatment. This subgroup was particularly intriguing given multiple prior datasets suggesting that antibiotics may diminish the ICI response; however, those participants who received both CBM588 and antibiotic therapy had an even more pronounced benefit with ICI therapy^12,22^. More recently, the same group showed a similar positive impact of CBM588 therapy in persons with NSCLC receiving chemo-immunotherapy. In a cohort of 106 participants with metastatic NSCLC treated with chemo-immunotherapy combinations, the use of CBM588 was associated with significantly higher OS, including participants treated with concurrent antibiotics. Interestingly, the survival benefit of CBM588 was most pronounced in participants with low (<1%) PDL1 levels^23^. In our prior study in mRCC, treatment with CBM588 in combination with a dual ICI-based regimen of ipilimumab and nivolumab resulted in an improvement in PFS^19^. In the current study, we again noted a similar signal of improved clinical efficacy with statistical improvement in ORR and numerically higher landmark PFS. The ORR (74%) and 6-month PFS (84%) with the combination of CBM588 with cabozantinib + nivolumab seen in our study are higher than in the previously reported CheckMate 9ER study^4^. Although the ORR (20%) in the control arm was numerically lower compared to the results of the CheckMate 9ER trial (56%), given the small sample size of our study and inherent differences in eligibility and enrolled participant population, the results of the study should be interpreted within the context of the randomized treatment arms.

Investigating potential changes in the microbiome profile with CBM588 supplementation was a crucial aim of our study, which was designed and conceptualized parallel to our prior trial with the primary endpoint of increase in *Bifidobacterium* spp. with CBM588 supplementation. In line with our prior study, we did not observe a significant increase in *Bifidobacterium* spp. as a result of CBM588-containing therapy. However, the consistent improvement in clinical outcomes seen in both studies suggests that other mechanisms or biomarkers not explored in our study could be driving this effect. One or more unclassified Ruminococcaceae genera were enriched in on-therapy samples from participants in the CBM588 arm but not in the control arm. A higher abundance of bacteria of the Ruminococcaceae family was one of the first gut microbiome features to be associated with favorable outcomes and response to ICI treatment^14^. In persons with melanoma, higher levels of Ruminococcaceae in the gut correlated with increased circulating effector CD4^+^ and CD8^+^ T cells and higher infiltrating antitumor immune cells, as well as a maintained cytokine response to anti-PD1 therapy. It should be noted that the observations made herein regarding Ruminococcaceae (along with other observations related to changes in the microbiome profile) are distinct from our prior study evaluating nivolumab and ipilimumab with CBM588. It is possible that the use of a VEGFR-TKI (cabozantinib) in the current study could account for the differing evolution in microbiome profile across these studies. Ultimately, however, only larger randomized efforts including both ICI-based regimens will clarify whether this indeed accounts for the difference.

Another notable finding from our stool metabolomic analysis was an enrichment of menaquinol-8 biosynthesis III and 1,4-dihydroxy-6-naphthoate biosynthesis II in on-therapy samples from participants treated with CBM588. Both of these pathways have been implicated in the vitamin K2 biosynthesis by intestinal microbiota^24,25^. However, in the control arm, six superpathways of biosynthesis of different forms of menaquinole, a reversible redox component of the electron transfer chain, were depleted^25^. Although traditionally linked to maintaining bone health and working synergistically with vitamin D, vitamin K2 was also shown to have immunomodulatory and antitumor effects in preliminary studies^26^. Although these findings should be regarded as hypothesis generating, the underlying mechanisms behind the improved outcomes seen in our study still need to be examined in the context of larger clinical trials.

Key differences in blood-based biomarker results were observed across the two trials we conducted. In our prior study, we observed an increase in circulating cytokines such as C-C motif chemokine ligand 2 (CCL2), CCL4, C-X-C motif chemokine ligand 9 (CXCL9) and CXCL10 in participants receiving CBM588 (ref. ^19^). In the current study, levels of circulating IL-12, eotaxin and IFNγ were significantly higher on therapy (week 13) compared to baseline in the CBM588 arm. In contrast, no change in cytokine levels was seen in the control arm. The differences in cytokine profile seen across both studies could be because of the differential immunomodulatory effects of the ICI-based regimens examined. While ICI treatment incorporating CTLA4 and PD1 inhibitors could induce immune stimulation with a global increase in cytokines (for example, IL-1β, IL-2, IL-6, IL-8, IL-10 and IFNγ), it is important to acknowledge that VEGFR-TKIs also appear to have immunomodulatory properties^27^. Cabozantinib, in particular, has been shown to increase the ratio of effector CD8^+^ T cells to regulatory T cells in the periphery^28^. However, this effect is not consistent across VEGFR-TKIs; for example, while sunitinib and pazopanib appear to have immunostimulatory properties, sorafenib may have the opposite effect^29,30^.

Taken together with the two small prospective trials now completed in mRCC, the data with CBM588 are intriguing enough that larger studies should be completed in order to confirm activity. The National Cancer Institute (NCI)-supported Southwest Oncology Group (SWOG) has plans to conduct a multi-institutional phase 3 clinical trial comparing an ICI-based combination therapy with or without CBM588, which will assess microbiome modulation in persons with advanced cancer. On the basis of our previous study, CBM588 may well have activity in other settings where dual ICI treatment is a standard, including but not limited to NSCLC and melanoma^31,32^. Given our current data, it may also be worthwhile to explore the addition of CBM588 to other malignancies where combinations of VEGF-directed therapy and ICI are standard, such as hepatocellular carcinoma^33^.

Recently, the concept of antibiotic preconditioning has become a point of discussion in prospective studies aiming to manipulate the microbiome. Indeed, in a retrospective experience evaluating CBM588 in persons with NSCLC receiving ICIs, concomitant use of antibiotics led to superior outcomes^21^. A counterpoint to this is a plethora of literature suggesting that antibiotics, in general, can diminish outcomes with multiple forms of immunotherapy, ranging from ICIs to chimeric antigen receptor (CAR) T cell treatments^22,34–36^. Prospective evidence for this approach comes from a recent study evaluating SER-401, an oral Firmicutes-enriched spore formulation^36^. In this trial, participants with advanced melanoma were randomized to receive vancomycin preconditioning followed by SER-401 with nivolumab or a placebo-conditioning regimen followed by nivolumab with placebo. The response rate was 25% in the SER-401 arm compared to 67% in the placebo arm. Detailed preclinical efforts accompanying this study showed that vancomycin preconditioning led to changes in the microbiome of representative preclinical models that could impede response. Of course, much larger efforts are needed to determine the role of antibiotic preconditioning; at the moment, it should be approached with caution.

Limitations of the current study include, first and foremost, the modest sample size. The current study was designed before the results of our previous trial were available; therefore, we picked an identical biological endpoint. With knowledge of these results, we might have designed a larger study to assess efficacy appropriately. Another limitation that must be acknowledged is the heterogeneity in the baseline characteristics of study arms. The impact of this is quite challenging to predict; for instance, while there was a larger proportion of participants on the experimental arm with favorable risk disease (45% versus 30%), there were also more participants with papillary histology (10% versus 0%). Favorable risk and papillary histology would be predicted to have a positive and negative impact on outcomes, respectively. It is worth noting that many potentially prognostic characteristics (for example, presence of bone metastases and previous nephrectomy) were balanced. Valuable insight might have been gained from the plasma or stool assessment of metabolites such as butyrate; however, these analytes are very labile. Our collection methodologies for stool and blood did not have time or temperature sensitivity that would have allowed for satisfactory characterization. In addition, our study may have also been confounded by differences in diet among study participants. Through monitoring of detailed dietary logs, we attempted to ensure that participants had not ingested yogurt or other bacterially fortified foods. However, recent data suggest that dietary constituents such as fiber could have a profound effect on microbiome composition and, through increasing the proportion of certain bacteria (for example, Ruminococcaceae family), enhance clinical outcomes^37^. These elements were not accounted for in our study design. The use of a placebo control arm would have also indeed strengthened our findings. Although a detailed review of dietary logs did not reveal any deviations from protocol-stipulated criteria for supplement use (for example, no use of probiotics or bacterially fortified foods), it is hard to fully account for any surreptitious use of these agents. In the aforementioned phase 3 trial planned to evaluate CBM588, a placebo control arm has been suggested.

In summary, the totality of our data offers a preliminary signal to suggest that CBM588 may complement ICI-based regimens (either as ICI doublets or in combination with VEGFR-TKIs) for the first-line treatment of mRCC. However, it is critical to acknowledge that these observations are only hypothesis generating. Given the limited sample size across both experiences, plans for larger studies to confirm our findings are underway. The phase 3 NCI-sponsored cooperative group trial is currently planned to include persons with previously untreated mRCC and randomize participants to receive ICI-based therapy with CBM588 or placebo. In this study, participants may receive ICI-based therapies, including nivolumab + ipilimumab, or (based on the current trial) a combination of VEGF-TKI with ICI^19^. As the addition of CBM588 does not appear to add toxicity to treatment, this LBP could represent a safe approach to enhance clinical outcomes in earlier stages of the disease and in other tumor types.

## Methods

### Participant eligibility

This was a randomized, single-center, open-label, investigator-initiated clinical trial (NCT05122546). Participant inclusion criteria included the following: male or female of any ethnicity or race with age ≥ 18 years and histologically confirmed advanced or mRCC with a clear cell, papillary or sarcomatoid component. No prior systemic therapy for mRCC was permitted. Prior adjuvant or neoadjuvant treatment for completely resected RCC was allowed if disease recurrence occurred at least 6 months after the last dose of adjuvant or neoadjuvant therapy. Participants were required to have measurable disease as per the Response Evaluation Criteria in Solid Tumors (RECIST 1.1), a Karnofsky performance status ≥ 70%, adequate organ and marrow function within 14 days before the first dose of study treatment and improvement of toxicities related to any prior treatments to baseline or Grade ≤1 per Common Terminology Criteria for Adverse Events (CTCAE) v5 unless adverse event(s) were clinically nonsignificant and/or stable on supportive therapy. Participants had to be capable of understanding and complying with the protocol requirements and had to have signed the informed consent document. Sexually active fertile subjects and their partners were required to agree to use medically accepted methods of contraception during the study. This requirement was 4 months after the last dose of cabozantinib or 5 months after the last dose of nivolumab for women with childbearing potential and 7 months after the last dose of nivolumab for men. Female participants of childbearing potential could not be pregnant at screening. The sex and gender of participants were determined on the basis of self-report. Further analysis of sex or gender in regard to outcomes was not carried out as advanced RCC occurs in both males and females.

Exclusion criteria included prior treatment with cabozantinib, the current use of or intent to use probiotics, yogurt or bacterially fortified foods during the period of treatment, active interstitial lung disease (ILD) or pneumonitis or a history of ILD or pneumonitis requiring treatment with systemic steroids and a known medical condition that would increase the risk associated with study participation. Receipt of any type of small-molecule kinase inhibitor within 2 weeks before the first dose of study treatment, cytotoxic, biologic or other systemic anticancer therapy within 4 weeks before the first dose of study treatment or radiation therapy for bone metastasis within 2 weeks or any other radiation therapy within 4 weeks before the first dose of study treatment was not allowed. Persons with known brain metastases or cranial epidural disease were excluded unless adequately treated with radiotherapy and/or surgery and stable for at least 4 weeks before the first dose of study treatment. Other exclusion criteria included concomitant use of anticoagulation, administration of a live, attenuated vaccine within 30 days before the first dose of study treatment, uncontrolled, significant intercurrent or recent illness, clinically significant hematuria, hematemesis or hemoptysis, cavitating pulmonary lesion(s) or known endotracheal or endobronchial disease manifestation, lesions invading or encasing any major blood vessels, other clinically significant disorders that would preclude safe study participation, major surgery within 2 weeks before the first dose of study treatment, minor surgeries within 10 days before the first dose of study treatment, corrected QT interval > 500 ms per electrocardiogram, pregnant or lactating women, inability to swallow tablets or unwillingness or inability to receive intravenous administration, previously identified allergy or hypersensitivity to components of the study treatment formulations or history of severe infusion-related reactions to monoclonal antibodies and any other active malignancy at the time of first dose of study treatment or diagnosis of another malignancy within 3 years before first dose of study treatment that required active treatment, except for localized curable cancers. Full inclusion and exclusion criteria are presented in the study protocol (Supplementary Information). All participants were required to withhold from consuming other probiotics or any bacterially fortified foods while on the protocol, regardless of their assigned treatment arm. The study was approved by the US Food and Drug Administration and by the City of Hope Institutional Review Board. Written informed consent was supplied by all participants in accordance with the Declaration of Helsinki. The full clinical trial protocol is included in the Supplementary Information.

### Study design and treatment

Eligible participants were randomized in a 2:1 fashion to receive the combination of cabozantinib and nivolumab with or without CBM588. To generate the random allocation sequence, permutation within a block was conducted using the ‘sample’ function in R, without replacement, with a set seed documented. A fixed block size of 6 was used. The study statistician supplied the randomization log to the City of Hope central data coordinating center (DCC). This file was kept in a secure computer folder within the DCC and not shared with anyone outside the DCC. The block size and method chosen were not shared with the clinical team by the statistician or DCC, nor was the allocation sequence. The study statistician generated the allocation sequence and the DCC staff conducted the actual assignment. Neither the study statistician nor the DCC staff had any contact with the participants. Access to the randomization table was restricted to the DCC and lead statistician only.

In both treatment arms, participants received cabozantinib (40 mg) by mouth daily along with nivolumab (480 mg) once a month by intravenous infusion. Participants in the experimental arm also received CBM588 (80 mg) by mouth twice daily. CBM588 was manufactured under Current Good Manufacturing Practice (cGMP) at Miyarisan Pharmaceutical Company. Each gram of manufactured CBM588 contained 40 mg of CBM588 powder, the active pharmaceutical ingredient, and 2 × 10^8^ colony-forming units of *C*. *butyricum*. Participants in the experimental arm were instructed to take CBM588 indefinitely as long as they were in the study. Participants were required to maintain a diet and log their stool while in protocol therapy, irrespective of the treatment arm. The potential use of systemic antibiotics was monitored during the study (Supplementary Table 2). Treatment was continued until the completion of protocol therapy, unacceptable adverse events, withdrawal of consent or disease progression.

### Stool collection, DNA extraction and metagenomics sequencing

Participants underwent response evaluation every 12 weeks with either computed tomography or magnetic resonance imaging of the chest, abdomen and pelvis. Response evaluation was performed by a study radiologist who documented the RECIST response at each predesignated time point independent of the clinical team. Safety assessments were conducted every 4 weeks during protocol therapy and at 30 days after the last dose. Stool collection for assessment of the primary endpoint was conducted before treatment at baseline and on therapy at the start of week 13 using the OMNIgene Gut Collection Kit. Samples underwent genomic DNA extraction utilizing the MagMAX Microbiome Ultra Nucleic Acid Isolation Kit protocol. The metagenomic DNA was sequenced using the NextSeq 500/550 High-Output KT version 2.5 kit, specifically designed for metagenome sequencing, on the Illumina NextSeq platform.

### Metagenomics bioinformatics

The human reads were identified and filtered out by aligning them to the human genome GRCh38.p7 (https://www.ncbi.nlm.nih.gov/datasets/genome/GCF_000001405.33/, National Center for Biotechnology Information (NCBI) RefSeq assembly number: GCF_000001405.33) using BowTie2 and removing reads that matched, thereby depleting potential contamination originating from the host genome. Demultiplexed reads were subjected to trimming using Trimmomatic 0.33, which eliminates adapter sequences and low-quality bases, enhancing the accuracy of downstream analyses. Taxonomic profiling of the trimmed metagenomic reads was conducted using MetaPhlAn 4.0, enabling the identification and quantification of microbial taxa present in the sample. Functional profiling was performed using HUMAnN3, which annotates open reading frames and provides comprehensive information on gene family abundances, metabolic pathway coverage and abundances^38^.

### Cytokine and immune cell analyses

To evaluate the concentrations of cytokines and chemokines, peripheral blood samples from participants were obtained using 10-ml cell preparation tubes (BD Biosciences) at baseline and at weeks 9, 13, 17 and 25. All samples underwent processing within a 4–6-h window after collection. The separation of peripheral plasma from peripheral blood mononuclear cells (PBMCs) was achieved through centrifugation at 1,800*g* for 20 min. Subsequently, the plasma was extracted and stored at −80 °C until analysis. A total of 30 circulating cytokines (IL-1RA, IL-1b, IL-2, IL-2R, IL-4, IL-5, IL-6, IL-7, IL-8, IL-10, IL-12, IL-13, IL-15, IL-17, eotaxin, epidermal growth factor, hepatocyte growth factor, fibroblast, G-CSF, GM-CSF, IFNα, IFNγ, monokine-induced IFNγ, IFNγ-induced protein 10, monocyte chemoattractant protein 1, macrophage inflammatory protein (MIP)1α, MIPβ, RANTES (regulated on activation, normal T cell expressed and secreted), tumor necrosis factor-α and VEGF) were assessed using the Luminex Flexmap 3D system (Biotechne). Changes in circulating cytokine levels between baseline and week 13 (±7 days) were examined across the treatment arms and between responders and nonresponders to investigate the impact of CBM588 on the immune system. The remaining peripheral blood was then resuspended in a 1:1 ratio in FBS and 10% DMSO and stored in liquid nitrogen until flow cytometric analysis. The remaining cell suspension was transferred to conical propylene tubes, washed in complete RPMI medium and recentrifuged at 250*g* for 7 min at room temperature to isolate PBMCs. PBMCs were then immersed in a mixture of PBS, fetal calf serum and sodium azide with Fc III/IIR-specific antibody (commercially available Fc III/IIR-specific antibodies validated by Biolegend, Invitrogen and BD) to block nonspecific binding and the cells were stained with viability dye-Zombie NIR and different combinations of fluorochrome-labeled antibodies to CD3–BUV496, CD4–PeCy7, CD8–BUV805 and intracellular FoxP3–PE. Flow cytometry data were collected using Cytek Aurora and analyzed using FlowJo software version 10.7.1.

### Statistical analyses

The primary endpoint of this pilot study was to determine the change in *Bifidobacterium* spp. composition of stool from baseline to week 13 of therapy. Key secondary endpoints included a comparison of the Shannon index (a measure of microbial alpha diversity) from baseline to week 13 of therapy, along with efficacy measures such as best ORR and PFS by RECIST 1.1 criteria, with cabozantinib + nivolumab alone versus cabozantinib + nivolumab with CBM588. With the enrollment of 20 participants on the CBM588-containing experimental arm and 10 participants on the non-CBM588 arm, the study had 80% power to detect a difference of 1 s.d. (common for the change in *Bifidobacterium* spp.) between the mean change detected in the two groups using a two-group *t*-test with a one-sided type I error of 0.05.

Participant characteristics were summarized using descriptive statistics. Microbiome composition comparisons were performed using QIIME 2 (ref. ^38^). To identify differentially abundant microbial features or functional pathways, we used the ANCOM-BC method^39^. ANCOM-BC is a statistical method for identifying differentially abundant microbial taxa in microbiome studies, taking into account the compositional nature of the data. ANCOM-BC calculations include transforming raw counts using a central log ratio transformation, applying bias correction and performing statistical tests using a linear model. Multiple comparisons are adjusted to control for false discovery rates to ensure that identified differences in microbial abundance are statistically valid (*q* value). Beta diversity was assessed using the Bray–Curtis and Jaccard dissimilarity measures and permutational multivariate analysis of variance was employed for the statistical analysis of beta diversity. Alpha diversity was evaluated using the Shannon diversity index and Pielou’s evenness with the Kruskal–Wallis test^40,41^. PFS was assessed as the time from enrollment to radiographic progression and was estimated using the Kaplan–Meier method and compared between treatment arms using the Cox proportional hazards model. Median follow-up was calculated using the reverse Kaplan–Meier method. The association between the treatment arm and overall response as per RECIST criteria was evaluated using Fisher’s exact test. A two-sided Wilcoxon matched-pairs test was used to compare the levels of cytokines at the two prespecified time points. A two-sided Mann–Whitney *U* test was used for comparisons between the two arms. Cytokine and immune cell populations were analyzed using GraphPad Prism version 8.4.2. Clinical data were analyzed using R version 4.3.0.

### Reporting summary

Further information on research design is available in the Nature Portfolio Reporting Summary linked to this article.

## Online content

Any methods, additional references, Nature Portfolio reporting summaries, source data, extended data, supplementary information, acknowledgements, peer review information; details of author contributions and competing interests; and statements of data and code availability are available at 10.1038/s41591-024-03086-4.

## Supplementary information

Supplementary Information Supplementary Fig. 1, Tables 1 and 2, study protocol and data transfer agreement.

Reporting Summary

## Figures and Tables

**Extended Data Table 1:**
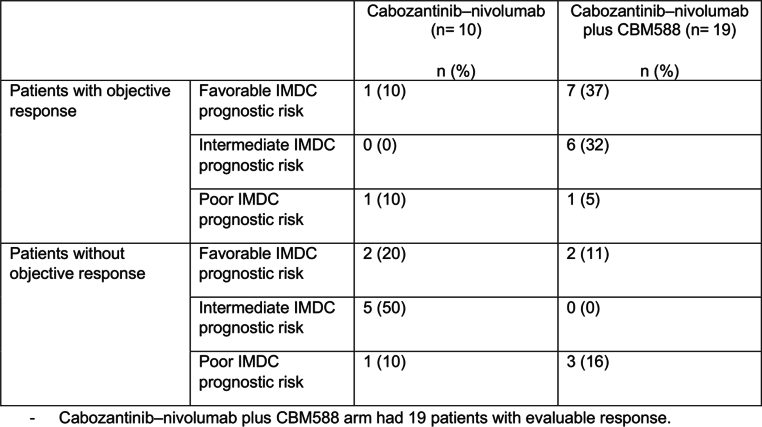
Objective response rate by IMDC risk category

Objective response assessment in cabozantinib–nivolumab arm (n = 10) and cabozantinib–nivolumab plus CBM588 arm (n = 19) by IMDC risk category.

## Data Availability

Human genome GRCh38.p7 was accessed through https://www.ncbi.nlm.nih.gov/datasets/genome/GCF_000001405.33/ (NCBI RefSeq assembly number: GCF_000001405.33). Metagenomic data sourced from stool, essential for replicating the analyses detailed in this paper, will be archived at the Translational Genomics Research Institute (TGen) and will be made available upon request. The authors have deferred depositing the participant genomic data in national and international public repositories based on institutional policies and the absence of statements in patient consent forms allowing controlled access distribution and genomic data availability. Deidentified individual participant whole metagenome libraries and clinical data, which form the foundation of the results presented in this article, are available for transfer on a specific secure server housed at TGen. Researchers interested in obtaining the data are required to complete and certify the data transfer agreement (DTA), available in the Supplementary Information, and submit requests to the principal investigator, S.K.P., with an approximate response time of 30 business days. The TGen data access committee will assess and vet proposals. Upon agreement to the terms outlined in the DTA, including the restricted use of data for specific research projects and the safeguarding of participant confidentiality, including but not limited to limiting the possibility of identification of participants in any way whatsoever, throughout the agreement’s duration, investigators and institutions will be granted access. TGen will facilitate the transfer of the requested deidentified data. This mechanism is expected to be through an Aspera High-Speed File Transfer Server. However, TGen retains the flexibility to modify the transfer method, ensuring that the appropriate levels of access authorization and control are maintained.

## References

[1] Choueiri, T. K. & Motzer, R. J. Systemic therapy for metastatic renal-cell carcinoma. *N. Engl. J. Med.* **376**, 354–366 (2017). 28121507 10.1056/NEJMra1601333

[2] Govindarajan, A. et al. Front-line therapy for metastatic renal cell carcinoma: a perspective on the current algorithm and future directions. *Cancers (Basel)* **14**, 2049 (2022). 35565179 10.3390/cancers14092049 PMC9106028

[3] Motzer, R. J. et al. Kidney Cancer, version 3.2022, NCCN clinical practice guidelines in oncology. *J. Natl Compr. Canc. Netw.* **20**, 71–90 (2022). 34991070 10.6004/jnccn.2022.0001 PMC10191161

[4] Choueiri, T. K. et al. Nivolumab plus cabozantinib versus sunitinib for advanced renal-cell carcinoma. *N. Engl. J. Med.* **384**, 829–841 (2021). 33657295 10.1056/NEJMoa2026982 PMC8436591

[5] Rini, B. I. et al. Pembrolizumab plus axitinib versus sunitinib for advanced renal-cell carcinoma. *N. Engl. J. Med.* **380**, 1116–1127 (2019). 30779529 10.1056/NEJMoa1816714

[6] Motzer, R. et al. Lenvatinib plus pembrolizumab or everolimus for advanced renal cell carcinoma. *N. Engl. J. Med.* **384**, 1289–1300 (2021). 33616314 10.1056/NEJMoa2035716

[7] Motzer, R. J. et al. Nivolumab plus ipilimumab versus sunitinib in advanced renal-cell carcinoma. *N. Engl. J. Med.* **378**, 1277–1290 (2018). 29562145 10.1056/NEJMoa1712126 PMC5972549

[8] Dizman, N., Arslan, Z. E., Feng, M. & Pal, S. K. Sequencing therapies for metastatic renal cell carcinoma. *Urol. Clin. North Am.* **47**, 305–318 (2020). 32600533 10.1016/j.ucl.2020.04.008

[9] Navani, V. et al. CABOSEQ: the effectiveness of cabozantinib in patients with treatment refractory advanced renal cell carcinoma: results from the International Metastatic Renal Cell Carcinoma Database Consortium (IMDC). *Clin. Genitourin. Cancer* **21**, 106 (2023). 10.1016/j.clgc.2022.07.008 35945133

[10] Pal, S. K. et al. Assessing the safety and efficacy of two starting doses of lenvatinib plus everolimus in patients with renal cell carcinoma: a randomized phase 2 trial. *Eur. Urol.* **82**, 283–292 (2022). 35210132 10.1016/j.eururo.2021.12.024

[11] Choueiri, T. K. et al. LBA8 phase III study of cabozantinib (C) in combination with nivolumab (N) and ipilimumab (I) in previously untreated advanced renal cell carcinoma (aRCC) of IMDC intermediate or poor risk (COSMIC-313). *Ann. Oncol.* **33**, S1430–S1431 (2022). 10.1016/j.annonc.2022.08.070

[12] Routy, B. et al. The gut microbiota influences anticancer immunosurveillance and general health. *Nat. Rev. Clin. Oncol.* **15**, 382–396 (2018). 29636538 10.1038/s41571-018-0006-2

[13] Matson, V. et al. The commensal microbiome is associated with anti-PD-1 efficacy in metastatic melanoma patients. *Science* **359**, 104–108 (2018). 29302014 10.1126/science.aao3290 PMC6707353

[14] Gopalakrishnan, V. et al. Gut microbiome modulates response to anti-PD-1 immunotherapy in melanoma patients. *Science* **359**, 97–103 (2018). 29097493 10.1126/science.aan4236 PMC5827966

[15] Davar, D. et al. Fecal microbiota transplant overcomes resistance to anti-PD-1 therapy in melanoma patients. *Science* **371**, 595–602 (2021). 33542131 10.1126/science.abf3363 PMC8097968

[16] Baruch, E. N. et al. Fecal microbiota transplant promotes response in immunotherapy-refractory melanoma patients. *Science* **371**, 602–609 (2021). 33303685 10.1126/science.abb5920

[17] Hagihara, M. et al. *Clostridium butyricum* modulates the microbiome to protect intestinal barrier function in mice with antibiotic-induced dysbiosis. *iScience* **23**, 100772 (2020). 31954979 10.1016/j.isci.2019.100772 PMC6970176

[18] Hagihara, M. et al. *Clostridium butyricum* enhances colonization resistance against *Clostridioides difficile* by metabolic and immune modulation. *Sci. Rep.* **11**, 15007 (2021). 34294848 10.1038/s41598-021-94572-z PMC8298451

[19] Dizman, N. et al. Nivolumab plus ipilimumab with or without live bacterial supplementation in metastatic renal cell carcinoma: a randomized phase 1 trial. *Nat. Med.* **28**, 704–712 (2022). 35228755 10.1038/s41591-022-01694-6 PMC9018425

[20] Hakozaki, T. et al. The gut microbiome associates with immune checkpoint inhibition outcomes in patients with advanced non-small cell lung cancer. *Cancer Immunol. Res.* **8**, 1243–1250 (2020). 32847937 10.1158/2326-6066.CIR-20-0196

[21] Tomita, Y. et al. Association of probiotic *Clostridium butyricum* therapy with survival and response to immune checkpoint blockade in patients with lung cancer. *Cancer Immunol. Res.* **8**, 1236–1242 (2020). 32665261 10.1158/2326-6066.CIR-20-0051

[22] Derosa, L. et al. Negative association of antibiotics on clinical activity of immune checkpoint inhibitors in patients with advanced renal cell and non-small-cell lung cancer. *Ann. Oncol.* **29**, 1437–1444 (2018). 29617710 10.1093/annonc/mdy103 PMC6354674

[23] Tomita, Y. et al. Association of *Clostridium butyricum* therapy using the live bacterial product CBM588 with the survival of patients with lung cancer receiving chemoimmunotherapy combinations. *Cancers (Basel)* **16**, 47 (2024). 10.3390/cancers16010047 PMC10778075 38201474

[24] Hiratsuka, T. et al. An alternative menaquinone biosynthetic pathway operating in microorganisms. *Science* **321**, 1670–1673 (2008). 18801996 10.1126/science.1160446

[25] Ren, L., Peng, C., Hu, X., Han, Y. & Huang, H. Microbial production of vitamin K2: current status and future prospects. *Biotechnol. Adv.* **39**, 107453 (2020). 31629792 10.1016/j.biotechadv.2019.107453

[26] Xv, F., Chen, J., Duan, L. & Li, S. Research progress on the anticancer effects of vitamin K2 (review). *Oncol. Lett.* **15**, 8926–8934 (2018). 29805627 10.3892/ol.2018.8502 PMC5958717

[27] Chehrazi-Raffle, A. et al. Circulating cytokines associated with clinical response to systemic therapy in metastatic renal cell carcinoma. *J. Immunother. Cancer* **9**, e002009 (2021). 33688021 10.1136/jitc-2020-002009 PMC7944971

[28] Apolo, A. B. et al. Cabozantinib in patients with platinum-refractory metastatic urothelial carcinoma: an open-label, single-centre, phase 2 trial. *Lancet Oncol.* **21**, 1099–1109 (2020). 32645282 10.1016/S1470-2045(20)30202-3 PMC8236112

[29] Pal, S. K. et al. Pazopanib as third line therapy for metastatic renal cell carcinoma: clinical efficacy and temporal analysis of cytokine profile. *J. Urol.* **193**, 1114–1121 (2015). 25286010 10.1016/j.juro.2014.09.110

[30] Hipp, M. M. et al. Sorafenib, but not sunitinib, affects function of dendritic cells and induction of primary immune responses. *Blood* **111**, 5610–5620 (2008). 18310500 10.1182/blood-2007-02-075945

[31] Hellmann, M. D. et al. Nivolumab plus ipilimumab in lung cancer with a high tumor mutational burden. *N. Engl. J. Med.* **378**, 2093–2104 (2018). 29658845 10.1056/NEJMoa1801946 PMC7193684

[32] Larkin, J. et al. Five-year survival with combined nivolumab and ipilimumab in advanced melanoma. *N. Engl. J. Med.* **381**, 1535–1546 (2019). 31562797 10.1056/NEJMoa1910836

[33] Finn, R. S. et al. Atezolizumab plus bevacizumab in unresectable hepatocellular carcinoma. *N. Engl. J. Med.* **382**, 1894–1905 (2020). 32402160 10.1056/NEJMoa1915745

[34] Stein-Thoeringer, C. K. et al. A non-antibiotic-disrupted gut microbiome is associated with clinical responses to CD19-CAR-T cell cancer immunotherapy. *Nat. Med.* **29**, 906–916 (2023). 36914893 10.1038/s41591-023-02234-6 PMC10121864

[35] Derosa, L. et al. Gut bacteria composition drives primary resistance to cancer immunotherapy in renal cell carcinoma patients. *Eur. Urol.* **78**, 195–206 (2020). 32376136 10.1016/j.eururo.2020.04.044

[36] Glitza, I. C. et al. Randomized placebo-controlled, biomarker-stratified phase Ib microbiome modulation in melanoma: impact of antibiotic preconditioning on cicrobiome and immunity. *Cancer Discov.* **4**, OF1–OF15 (2024). 10.1158/2159-8290.CD-24-0066 PMC11215408 38588588

[37] Spencer, C. N. et al. Dietary fiber and probiotics influence the gut microbiome and melanoma immunotherapy response. *Science* **374**, 1632–1640 (2021). 34941392 10.1126/science.aaz7015 PMC8970537

[38] Bolyen, E. et al. Reproducible, interactive, scalable and extensible microbiome data science using QIIME 2. *Nat. Biotechnol.* **37**, 852–857 (2019). 31341288 10.1038/s41587-019-0209-9 PMC7015180

[39] Lin, H. & Peddada, S. D. Analysis of compositions of microbiomes with bias correction. *Nat. Commun.* **11**, 3514 (2020). 32665548 10.1038/s41467-020-17041-7 PMC7360769

[40] Knight, R. et al. Best practices for analysing microbiomes. *Nat. Rev. Microbiol.* **16**, 410–422 (2018). 29795328 10.1038/s41579-018-0029-9

[41] Galloway-Peña, J. & Hanson, B. Tools for analysis of the microbiome. *Dig. Dis. Sci.* **65**, 674–685 (2020). 32002757 10.1007/s10620-020-06091-y PMC7598837

