# Supplementary material for: Cabozantinib and nivolumab with or without live bacterial supplementation in metastatic renal cell carcinoma: a randomized phase 1 trial
Source: Nat Med. 2024 Jun 28;30(9):2576–85. doi: 10.1038/s41591-024-03086-4 (PMC11405272; doi:10.1038/s41591-024-03086-4)
Supplement: Supplementary file 1 — Supplementary Fig. 1, Tables 1 and 2, study protocol and data transfer agreement. [file 41591_2024_3086_MOESM1_ESM.pdf]

nature medicine

Supplementary information https://doi.org/10.1038/s41591-024-03086-4

Cabozantinib and nivolumab with or without live bacterial supplementation in metastatic renal cell carcinoma: a randomized phase 1 trial

In the format provided by the authors and unedited

Supplementary Fig 1. Flow cytometric gating strategies. The starting cell population was identified using FSC/SSC gates, and then single cells were gated using FSC-H/FSC-A. Viable cells were then gated as SSC-A/Zombie NIR negative cells before gating on SSC-A/CD3+ populations as the initial CD3+ T cell population. Following the identification of CD3+ T cells, CD4+ and CD8+ T cells were then gated as CD4+ PeCy7 cells or CD8+ BUV805+ cells, respectively. For CD4+ FoxP3+ T regulatory cells, cells were identified as CD3+ and CD4+ T cells before gating on SSC-A/Foxp3+ T cells. The gates for FoxP3+ T cells were based on fluorescence minus one (FMO) control, as shown in the supplementary data.

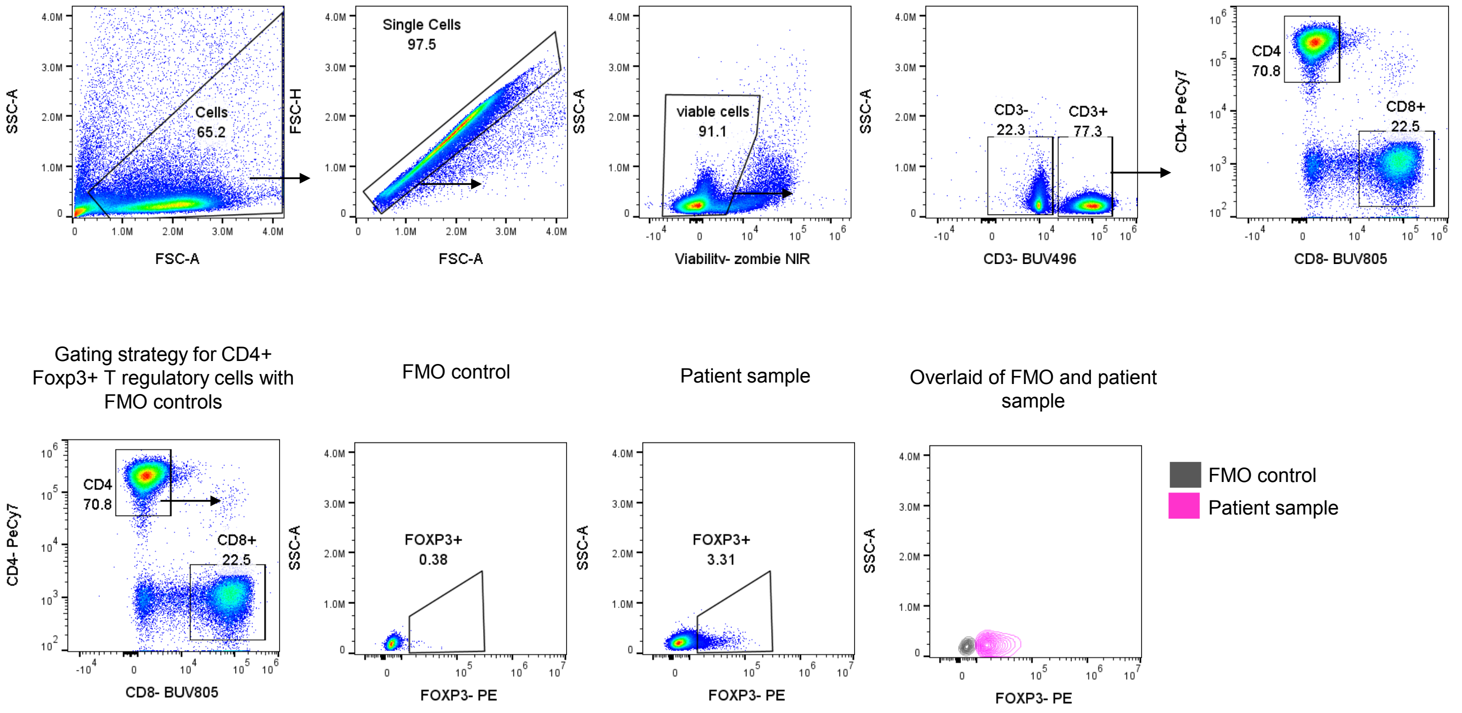

Supplementary Table 1. All adverse events recoded in the study.

Adverse Event	Cabozantinib/nivolumab (n = 10)					Cabozantinib/nivolumab plus CBM588 (n = 20)				
	n (%)					n (%)				
	Grade 1	Grade 2	Grade 3	Grade 4	Grade 5	Grade 1	Grade 2	Grade 3	Grade 4	Grade 5
Total subjects with an event	2 (20%)	4 (40%)	3 (30%)	1 (10%)		5 (25%)	5 (25%)	8 (40%)		
Anemia	4 (40%)					1 (5%)	1 (5%)			
Eosinophilia						2 (10%)				
Adrenal insufficiency	1 (10%)					1 (5%)				
Hyperthyroidism	1 (10%)									
Hypothyroidism	1 (10%)					1 (5%)	1 (5%)			
Increased Free T4 level						3 (15%)				
Flashing lights						1 (5%)				
Abdominal distension						1 (5%)				
Abdominal pain						2 (10%)				
Bloating	1 (10%)									
Constipation	3 (30%)					1 (5%)				
Diarrhea	4 (40%)					7 (35%)	1 (5%)	2 (10%)		
Dry mouth						2 (10%)				
Dysphagia						1 (5%)				
Flatulence						1 (5%)				
Gastrointestinal pain	1 (10%)									
Gingival pain						1 (5%)				
Hemorrhoids							1 (5%)			
Mucositis oral	3 (30%)					3 (15%)				
Nausea	1 (10%)					6 (30%)				
Vomiting			1 (10%)			2 (10%)				
Upper gastrointestinal hemorrhage			1 (10%)							
Gastroesophageal reflux disease	1 (10%)									
Anorectal pain						1 (5%)				
Immunotherapy induced hepatitis			1 (10%)					1 (5%)		
Mouth blister						1 (5%)				
Fatigue	6 (60%)					1 (5%)0				
Pain						4 (20%)				
Edema limbs						1 (5%)				
Non-cardiac chest pain						1 (5%)				
Progression of Disease										
Cholecystitis	1 (10%)									
Rash pustular						1 (5%)	1 (5%)			
Urinary tract infection	1 (10%)									
Papulopustular rash	1 (10%)					3 (15%)				
Fall		1 (10%)								
Infusion related reaction						1 (5%)				
Stomal ulcer	1 (10%)									
Activated partial thromboplastin time prolonged	2 (20%)									
Alanine aminotransferase increased	3 (30%)		2 (20%)			6 (30%)	2 (10%)	1 (5%)		

Alkaline phosphatase increased	1 (10%)					3 (15%)				
Aspartate aminotransferase increased	4 (40%)		2 (20%)			6 (30%)	2 (10%)			
Blood bilirubin increased	2 (20%)					3 (15%)				
Blood lactate dehydrogenase increased	3 (30%)					2 (10%)				
Cholesterol high	1 (10%)									
Creatinine increased	2 (20%)					6 (30%)				
Lipase increased	1 (10%)	1 (10%)								
Lymphocyte count decreased	1 (10%)					2 (10%)				
Neutrophil count decreased	3 (30%)					6 (30%)	1 (5%)			
Platelet count decreased	1 (10%)					6 (30%)				
Thyroid stimulating hormone increased	8 (80%)					6 (30%)	2 (10%)			
Weight loss	1 (10%)	1 (10%)				1 (5%)	1 (5%)			
White blood cell decreased	1 (10%)	1 (10%)	1 (10%)			6 (30%)	2 (10%)			
Hemoglobin increased	1 (10%)									
Appetite changes						1 (5%)				
Decreased ANC						1 (5%)				
Decreased RBC						1 (5%)				
Dysgeusia						1 (5%)				
Hypoproteinemia						3 (15%)				
Increased blood urea	1 (10%)					1 (5%)				
Mouth sore/sensitivity	1 (10%)					2 (10%)				
TSH decreased						1 (5%)				
Anorexia	2 (20%)					6 (30%)				
Dehydration						1 (5%)				
Hypercalcemia						1 (5%)				
Hyperglycemia	3 (30%)					5 (25%)				
Hyperkalemia	1 (10%)						1 (5%)			
Hypermagnesemia								1 (5%)		
Hypertriglyceridemia	1 (10%)									
Hypoalbuminemia	2 (20%)	1 (10%)				4 (20%)	1 (5%)			
Hypocalcemia	1 (10%)	1 (10%)	1 (10%)			3 (15%)	1 (5%)			
Hypoglycemia	3 (30%)					6 (30%)				
Hypokalemia	1 (10%)	1 (10%)				1 (5%)				
Hyponatremia	5 (50%)			1 (10%)		7 (35%)	1 (5%)			
Arthralgia						1 (5%)		1 (5%)		
Back pain	1 (10%)					1 (5%)				
Chest wall pain						1 (5%)				
Muscle cramp						2 (10%)				
Myalgia	1 (10%)					3 (15%)				
Pain in extremity	1 (10%)					1 (5%)				
Generalized muscle weakness	1 (10%)					1 (5%)				
Joint stiffness of left hand						1 (5%)				
Joint Swelling Hands						1 (5%)				
Dizziness	1 (10%)					1 (5%)				
Dysgeusia	3 (30%)					5 (25%)				
Headache	3 (30%)					2 (10%)				

Memory impairment						1 (5%)				
Paresthesia	1 (10%)									
Burning both feet	1 (10%)									
Lightheadedness						1 (5%)				
Numbness on both feet	1 (10%)									
Anxiety						1 (5%)				
Insomnia	1 (10%)									
Libido decreased						1 (5%)				
Apathy						1 (5%)				
Cough	1 (10%)					1 (5%)		1 (5%)		
Dyspnea						1 (5%)				
Epistaxis	1 (10%)					1 (5%)				
Hoarseness						3 (15%)				
Nasal congestion						1 (5%)				
Pneumonitis								1 (5%)		
Productive cough						1 (5%)				
Sore throat						1 (5%)	1 (5%)			
Voice alteration						1 (5%)				
Shortness of breath						1 (5%)				
Alopecia	1 (10%)					2 (10%)				
Bullous dermatitis						2 (10%)		1 (5%)		
Dry skin						1 (5%)				
Eczema						1 (5%)				
Erythroderma	1 (10%)									
Photosensitivity						1 (5%)				
Pruritus	1 (10%)									
Rash maculo-papular	4 (40%)					4 (20%)				
Palmar-plantar erythrodysesthesia syndrome	7 (70%)	1 (10%)				8 (40%)	2 (10%)	1 (5%)		
Abrasion	1 (10%)									
Angular cheilitis						1 (5%)				
Dermatitis	1 (10%)									
Erythema	1 (10%)					1 (5%)				
Erythematous patch	1 (10%)									
Left foot Soreness						1 (5%)				
Neoplasm of uncertain behavior						1 (5%)				
Nivolumab induced dermatitis								1 (5%)		
Plantar warts						1 (5%)				
Rash						1 (5%)				
Scratches on leg and back							1 (5%)			
Skin lesion on Left lower leg	1 (10%)									
Skin lesion on Right upper arm						1 (5%)				
Skin sensitivity						1 (5%)				
Soreness						1 (5%)				
Swelling on right side of temple	1 (10%)									
Thinning hair						1 (5%)				
Hypertension	1 (10%)	6 (60%)	1 (10%)			1 (5%)	7 (35%)	1 (5%)		

Supplementary Table 2. Patients antibiotic use summary.

ID	Study arm	Days from first day of treatment	Antibiotic(s)	Reason
1	Experimental	None	N/A	N/A
2	Experimental	56 to 59	Ceftriaxone	Post Covid-19 pneumonia
		56 to 59	Cefixime	Post Covid-19 pneumonia
3	Control	322 to 325	Ciprofloxacin	Urinary tract infection
4	Experimental	None	N/A	N/A
5	Control	None	N/A	N/A
6	Experimental	None	N/A	N/A
7	Control	47 to 96	Sulfamethoxazole-trimethoprim	Prophylaxis
8	Experimental	282 to 292	Ciprofloxacin	Systemic inflammatory response syndrome
9	Control	128 to 137	Sulfamethoxazole-trimethoprim	Cellulitis
10	Experimental	349 to 356	Ciprofloxacin	Conjunctivitis
11	Experimental	None	N/A	N/A
12	Experimental	None	N/A	N/A
13	Experimental	41 to 48	Doxycycline	Cellulitis
14	Experimental	280 to 304	Amoxycillin	Dental infection
	Experimental	304 to 311	Clindamycin	Dental infection
15	Control	45 to 57	Sulfamethoxazole-trimethoprim	Prophylaxis
		171 to 201	Sulfamethoxazole-trimethoprim	Prophylaxis
16	Experimental	420 to 427	Amoxicillin/Clavulanic acid	Upper respiratory tract infection
17	Experimental	195 to 199	Azithromycin	Flu
18	Experimental	None	N/A	N/A
19	Experimental	31 to 41	Ciprofloxacin	Urinary tract infection
20	Experimental	None	N/A	N/A
21	Experimental	98 to 314	Clotrimazole Troche	Yeast infection
22	Control	None	N/A	N/A
23	Experimental	51 to 58	Amoxycillin	Dental infection

		112 to 119	Amoxicillin/Clavulanic acid	Sinusitis
**24**	Experimental	None	N/A	N/A
**25**	Experimental	None	N/A	N/A
**26**	Control	None	N/A	N/A
**27**	Control	None	N/A	N/A
**28**	Control	None	N/A	N/A
**29**	Experimental	None	N/A	N/A
**30**	Control	None	N/A	N/A

- Systemic antibiotic use one week before and during the study.

Study Protocol.

CITY OF HOPE NATIONAL MEDICAL CENTER
1500 E. DUARTE ROAD
DUARTE, CA 91010

DEPARTMENT OF MEDICAL ONCOLOGY AND THERAPEUTICS RESEARCH

TITLE: **Pilot study to evaluate the biologic effect of CBM588 in combination with cabozantinib/nivolumab for patients with metastatic renal cell carcinoma**

CITY OF HOPE PROTOCOL NUMBER/VERSION: IRB # **21133** **Protocol Date: 01/21/22**

DATE(S)/ OF AMENDMENT(S)/REVISION(S):

COH Initial Review	Protocol Dated 04/29/21	Packet: 00
Amendment 1	Protocol Dated 01/21/22	Packet: 01
**Amendment 2**	**Protocol Dated 01/21/22(TP)**	**Packet:02**
Amendment 3 At Continuation	Protocol Dated 01/21/22	Packet: 03

**SPONSOR/IND NUMBER**	City of Hope/Osel, Inc/IND 18765
**DISEASE SITE:**	Renal Cell Carcinoma
**STAGE (If applicable):**	IV
**MODALITY:**	Pilot, Immunotherapy
**TYPE:**	Therapeutic
**PRINCIPAL INVESTIGATOR:**	Sumanta Pal, M.D.
**COLLABORATING INVESTIGATOR(S):**	Paul Frankel, PhD<br>Michael Valentine, MS<br>Marcin Kortylewski, PhD<br>David Engelthaler, PhD
**PARTICIPATING CLINICIANS:**	Tanya Dorff, M.D.<br>**Alexander Chehrazi Raffle ,M.D.**<br>**Adam Rock ,M.D.**

	Abhishek Tripathi, M.D.
**Research Coordinator**	JoAnn Hsu
	Luis Meza, M.D.
	Jasnoor Malhotra

EXPERIMENTAL DESIGN TABLE

The study will employ randomization (2:1) to cabozantinib/nivolumab with CBM588 or cabozantinib/nivolumab alone.

Treatment Arms

Dose Level	Number of Patients	Dose<br>CBM588<br>All Cycles	Cabozantinib<br>All Cycles	Nivolumab<br>All Cycles
**Arm 1**	**10**	None	40 mg oral daily	480 mg IV every 4 weeks
**Arm 2**	**20**	80 mg bid	40 mg oral daily	480 mg IV every 4 weeks

PROTOCOL SYNOPSIS

Protocol Title
Pilot study to evaluate the biologic effect of CBM588 in combination with cabozantinib/nivolumab for patients with mRCC
**Study Description**
The landscape of therapy for mRCC has changed drastically over the past decade. Most recently, cabozantinib/nivolumab has been introduced in the front-line setting based on data from the CheckMate9ER trial, showing a progression free survival benefit relative to suntinib. Although the data are encouraging for this strategy of combining checkpoint inhibition and tyrosine kinase inhibition, the vast majority of patients are not cured of their disease. A slight majority (55.7%) of patients respond to the combination therapy, and approximately 6% of patients will progress through therapy with no clinical benefit. Recent studies suggest that the gut microbiome may play a key role in modulating responses to immunotherapy. Our preliminary data from patients receiving immunotherapy for mRCC have shown that certain gut bacteria (e.g., *Bifidobacterium*) may predispose to response. We therefore propose assessing CBM588, a live biotherapeutic, in combination with cabozantinib/nivolumab. CBM588 is a strain of *Clostridium butyricum*, and it has been shown that butyric acid bacteria have immunomodulatory and anti-inflammatory effects on the intestinal epithelium, and can restore species such as *Bifidobacterium spp* and *Lactobacillus spp* to the gut. A previous study done within our group, COH IRB 18523, identified a higher clinical benefit rate with nivolumab/ipilimumab in combination with CBM-588 as opposed to nivolumab/ipilimumab alone. With 27 patients accrued, preliminary confidential data demonstrates that the median PFS for patients on nivolumab/ipilimumab with CBM-588 is about 41 weeks compared to about 9 weeks on nivolumab/ipilimumab alone. While the median OS has not yet been reached for patients on nivolumab/ipilimumab with CBM-588, the median OS is about 27 weeks for patients on nivolumab/ipilimumab alone.
**Objectives**
Primary Objectives<br>• (1) To determine the effect of CBM588 (in combination with cabozantinib/nivolumab) in modulation of the gut microbiome in patients with mRCC<br>*Secondary Objectives*<br>• (1) To evaluate the effect of CBM588 on the clinical efficacy of the cabozantinib/nivolumab combination<br>• (2) To determine the effect of CBM588 on systemic immunodulation of the cabozantinib/nivolumab combination in patients with mRCC<br>• (3) To determine the effect of CBM588 on toxicities such as diarrhea and nausea using CTCAE v5 criteria with the cabozantinib/nivolumab combination in patients with mRCC<br>• (4) To assess the effect of CBM588 on the change of metabolic pathways with the cabozantinib/nivolumab combination in patients with mRCC
**Evaluation Criteria and Endpoints**
Primary Endpoint

- (1) Change in *Bifidobacterium* composition of stool from baseline to week 12 of therapy on the CBM588 with cabozantinib/nivolumab vs cabozantinib/nivolumab alone.

Secondary Endpoints

- (1a) Comparison of the Shannon index (a measure of microbial diversity) from baseline to week 12 of therapy on the CBM588+ cabozantinib/nivolumab vs cabozantinib/nivolumab alone.
- (1b) Best overall response, by RECIST criteria, with cabozantinib/nivolumab alone vs cabozantinib/nivolumab with CBM588
- (1c) Progression-free survival (PFS), assessed as the duration of time from enrollment to progression, with cabozantinib/nivolumab alone vs cabozantinib/nivolumab with CBM588
- (2a) Comparison of the proportion of circulating Tregs at baseline to levels of circulating Tregs with cabozantinib/nivolumab alone vs cabozantinib/nivolumab with CBM588
- (2b) Comparison of the proportion of circulating MDSCs with cabozantinib/nivolumab alone versus cabozantinib/nivolumab with CBM588
- (2c) Comparison of IL-6, IL-8 and other cytokines/chemokines with cabozantinib/nivolumab alone versus cabozantinib/nivolumab with CBM588
- (3) Comparison of toxicities such as diarrhea and nausea using CTCAE v5 criteria with cabozantinib/nivolumab alone versus cabozantinib/nivolumab with CBM588
- (4) Comparison of metabolic pathways at baseline and week 12 of treatment, among patients receiving cabozantinib/nivolumab alone versus cabozantinib/nivolumab with CBM588.

**Study Details**	
**Phase:**	Phase I
**Study Population:**	• Age 18 or greater<br>• Histologically confirmed mRCC with clear cell histology, papillary or sarcomatoid histology<br>• Advanced, metastatic disease<br>• Planned treatment with cabozantinib/nivolumab<br>• No prior therapy with immune checkpoint inhibition
**Sample Size**	30 patients
**Accrual Duration:**	2 years
**Participant Duration:**	16 months
**Study Duration**	3.3 years
**Sites/Facilities Enrolling Participants:**	City of Hope
**Study Agents:**	CBM588
**Sponsor:**	Exelixis
**Industry Partner:**	Osel, Inc.
**Industry Partner Protocol ID:**	N/A

TABLE OF CONTENTS

SECTION		PAGE
Protocol Team		1
Experimental Design Table		2
Protocol Synopsis		3
Table of Contents		5
List of Tables and Figures		6
Abbreviations		7
1.0	Objectives and Endpoints	8
1.1	Primary Objectives	8
1.2	Secondary Objectives	8
2.0	Background	9
2.1	Cabozantinib/nivolumab for mRCC	9
2.2	The Microbiome in mRCC	9
2.3	CBM588	12
3.0	Eligibility Criteria	14
3.1	Inclusion Criteria	14
3.2	Exclusion Criteria	14
4.0	Participant Recruitment and Enrollment	18
5.0	Study Assessments and Procedures	18
5.1	Pre-treatment Period	18
5.2	Treatment Program Overview	199
5.3	Cycle Definition	20
5.4	Treatment Plan	19
5.5	Agent Administration	20
5.6	Assessments and Special Monitoring	21
5.7	Duration of Therapy and Criteria for Removal from Protocol Therapy	22
5.8	Follow-Up	23
5.9	Duration of Study Participation	23
5.10	Supportive Care, Prohibited and Concomitant Therapies/Medications	23
6.0	Dose Modification/ Delay Guidelines	25
6.1	CBM588	25
6.2	Nivolumab in combination with cabozantinib	25
7.0	Adverse Events and Unanticipated Problems	69
7.1	Definitions	69
7.2	Adverse Events of Special Interest	74
7.3	Follow-up of Adverse Events	76
7.4	Other Safety Considerations	78
7.5	Adverse Events and Unanticipated Problems	85
7.6	Assessment of Adverse Events	85
7.7	Reporting of Adverse Events	86
8.0	Agent Information	88
8.1	Nivolumab	88

8.2 Cabozantinib ... 89
8.3 CBM588 ... 90
9.0 Correlative/ Special Studies ... 91
9.1 Assessment of the Stool Microbiome ... 91
9.2 Assessment of Serum Cytokines ... 93
9.3 Correlative Banking ... 93
10.0 Study Calendar ... 94
11.0 Endpoint Definitions/Measurement of Effect ... 95
11.1 Change in Bifidobacterium composition of stool from baseline to week 12 ... 95
11.2 Best overall response, by RECIST criteria ... 95
11.3 Duration of time from enrollment to progression ... 98
11.4 Comparison of the Shannon index from baseline to week 12 ... 98
11.5 Comparison of the proportion of circulating Tregs ... 98
11.6 Comparison of the proportion of circulating MDSCs ... 98
11.7 Comparison of IL-6, IL-8 and other cytokines ... 100
12.0 Statistical Considerations ... 98
12.1 Study Design ... 98
12.2 Sample Size and Accrual Rate ... 99
12.3 Statistical Analysis Plan ... 99
13.0 Protocol Deviations and Single Subject Exceptions ... 100
13.1 Single Subject Exceptions ... 100
14.0 Conditions for Modifying the Protocol ... 100
15.0 Study Oversight, Quality Assurance, and Data & Safety Monitoring ... 101
15.1 All Investigator Responsibilities ... 101
15.2 Study Principal Investigator Responsibilities ... 101
15.3 Protocol Management Team (PMT) ... 101
15.4 Quality Assurance ... 101
15.5 Risk Determination ... 101
15.6 City of Hope Data and Safety Monitoring Committee ... 102
16.0 Conditions for Terminating the Study ... 102
17.0 Ethical and Regulatory Considerations ... 102
17.1 Patient Protection ... 102
17.2 Subject Identification ... 102
17.3 Informed Consent ... 103
18.0 Publications of Data and Protection of Trade Secrets ... 104
19.0 References ... 105
Appendix A: ECOG/Karnofsky Performance Status ... 108
Appendix B: Stool Collection Procedure ... 109
Appendix C: Study Log ... 113
Study Drug Medication Diary ... 116

LIST OF TABLES AND FIGURES

Table 10.0 Study Activity Calendar ... 94

ABBREVIATIONS

Abbreviation	Meaning
AE	Adverse Event
CFR	Code of Federal Regulations
COH	City of Hope
CR	Complete Response
CRC	Clinical Research Coordinator
CRF	Case Report Form
CTCAE	Common Terminology Criteria for Adverse Events
DLT	Dose Limiting Toxicity
DSMC	Data & Safety Monitoring Committee
ECOG	Eastern Cooperative Oncology Group
EOT	End of Treatment
FDA	Food and Drug Administration
GCP	Good Clinical Practice
HIV	Human Immunodeficiency Virus
IB	Investigator's Brochure
IDS	Investigational Drug Services
IND	Investigational New Drug
IRB	Institutional Review Board
mRCC	Metastatic Renal Cell Carcinoma
NCI	National Cancer Institute
OIDRA	Office of IND Development and Regulatory Affairs
OS	Overall Survival
OBED	Optimal Biologic Effective Dose
PD	Progressive Disease
PD-1	Programmed cell Death 1
PD-L1	Programmed cell Death Ligand 1
PFS	Progression-Free survival
PI	Principal Investigator
PMT	Protocol Management Team
PR	Partial Response
RR	Response Rate
SAE	Serious Adverse Event
SD	Stable disease
TKI	Tyrosine kinase inhibitor
UP	Unanticipated Problem

1.0 OBJECTIVES AND ENDPOINTS

1.1 Primary Objectives

Objectives	Endpoints/Measurements of Effect
○ To determine the effect of CBM588 (in combination with cabozantinib/nivolumab) on the gut microbiome in patients with mRCC	○ Primary: Change in *Bifidobacterium* composition of stool from baseline to week 12 of therapy<br>○ Secondary: Comparison of the Shannon index (a measure of microbial diversity) from baseline to week 12 of therapy

1.2 Secondary Objective

Objectives	Endpoints/Measurements of Effect
○ To evaluate the effect of CBM588 on the clinical efficacy of the cabozantinib/nivolumab combination	○ Best overall response, by RECIST criteria, with cabozantinib/nivolumab alone versus cabozantinib/nivolumab with CBM588<br>○ Progression-free survival (PFS), assessed as the duration of time from enrollment to progression, with cabozantinib/nivolumab alone vs cabozantinib/nivolumab with CBM588
○ To assess the effect of CBM588 on systemic immunodulation of the cabozantinib/nivolumab combination in patients with mRCC	○ Comparison of the proportion of circulating Tregs at baseline to levels of circulating Tregs with cabozantinib/nivolumab alone versus cabozantinib/nivolumab with CBM588<br>○ Comparison of the proportion of circulating MDSCs with cabozantinib/nivolumab alone versus cabozantinib/nivolumab with CBM588<br>○ Comparison of IL-6, IL-8 and other cytokines/chemokines with cabozantinib/nivolumab alone versus cabozantinib/nivolumab with CBM588
○ To assess the effect of CBM588 on toxicities such as diarrhea and nausea using CTCAE v5 criteria with the cabozantinib/nivolumab combination in patients with mRCC	○ Comparison of toxicities such as diarrhea and nausea using CTCAE v5 criteria with cabozantinib/nivolumab alone versus cabozantinib/nivolumab with CBM588
○ To assess the effect of CBM588 on the change of metabolic pathways with the cabozantinib/nivolumab combination in patients with mRCC	○ Comparison of metabolic pathways at baseline and week 12 of treatment, among patients receiving cabozantinib/nivolumab alone versus cabozantinib/nivolumab with CBM588.

2.0 BACKGROUND

2.1 Cabozantinib/nivolumab for mRCC

More than 76,000 patients will be diagnosed with renal cell carcinoma in 2021 in the United States. [1] At the time of initial presentation, one-third of the cases are metastatic, and the remainder have varying rates of progression to metastatic disease. The current treatment algorithm includes surgery followed by a sequence of FDA-approved agents. In the last decade, multiple agents have been approved for treatment of metastatic renal cell carcinoma (mRCC) including targeted therapies (sunitinib, sorafenib, axitinib, pazopanib, everolimus, axitinib, cabozantinib) or immunotherapy with either nivolumab monotherapy or the combination of nivolumab and ipilimumab.[2] Nivolumab and ipilimumab are fully human monoclonal antibodies targeting the programmed cell death protein 1 (PD-1) and the cytotoxic T lymphocyte antigen 4 (CTLA-4) pathway respectively. Inhibition of the PD-1 and CTLA-4 immune checkpoint pathways overcomes the immune escape mechanisms of the tumor cells and allow for enhanced antitumor activity. Recently, cabozantinib has been considered in the development of immunotherapy-TKI combinations due to its unique immunomodulatory profile and clinical efficacy as a monotherapy.

In the CheckMate9ER trial, the combination of nivolumab and cabozantinib was compared with sunitinib, which was considered the standard of care treatment for patients with mRCC. [3] Regardless of the risk classification of patients, as defined by the International mRCC Database Consortium (IMDC), progression free survival (PFS) was significantly improved with the immunotherapy-TKI combination versus sunitinib (18-month progression free survival ~50% vs ~30%, median PFS 16.6 months and 8.3 months, respectively). Additionally, the overall response rate (ORR) favored nivolumab with cabozantinib over sunitinib (55.7%, with 8% of patients showing complete response and 47.7% of patients showing partial response, vs. 27.1%, with 4.6% of patients showing a complete response and 22.6% of patients showing a partial response). While these response rates are impressive, it is important to note that they reflect the minority of patients with mRCC. Furthermore, approximately 6% of patients who receive the cabozantinib/nivolumab combination in the front-line setting will develop progressive disease. Thus, there are currently significant efforts to build on this regimen and identify novel approaches to improve on the clinical efficacy of the cabozantinib/nivolumab combination.[3]

2.2 The Microbiome in mRCC

In murine models, Vetizou and colleagues have reported that the activity of cytotoxic T-lymphocyte associated protein 4 (CTLA4)-blocking therapies is dependent upon the presence of *Bacteroides spp*. [4] In the context of PD-1, Sivan and colleagues have shown that the clinical activity of anti-PD-1 agents is related to *Bifidobacterium spp*. [5] These preclinical efforts have been bolstered by clinical data presented by Gopalakrishnan and colleagues who reported a correlation between the microbiome composition and response to anti-PD1 agents in 43 patients with metastatic melanoma. [6] Responders to anti-PD-1 agents had significantly higher microbial diversity versus non-responders (P=0.03), and responders higher levels of *Ruminococcacae spp*, respectively (P<0.01). [6] Routy et al conducted a similar analysis across a wider range of epithelial tumors, including 40 patients with mRCC and 60 patients with non-small cell lung cancer (NSCLC). They showed that the relative abundance of *Akkermansia municiphila* was closely linked to response.[7]

Our group has also interrogated the role of the microbiome in mRCC patients receiving nivolumab and TKIs. Patients receiving sunitinib or nivolumab were enrolled through two separate, IRB-approved protocols (COH IRB 16088 and COH IRB 16323, respectively). Sample collection was uniform across the studies, with the first stool specimen collected prior to the onset of treatment, the second collected after 4 weeks of therapy, and the third collected after 12 weeks of therapy. Patients were asked to submit an additional stool sample at the first onset of diarrhea, as well, if this occurred within the 12-week study period. Additional specimens were permitted at the patient's discretion. A manual was given to patients with detailed instructions for stool submission. Briefly, stool was collected at home in a sealed specimen container, surrounded by a cold pack at 4°C, and then placed in a Styrofoam shipping container. Stool was shipped overnight, frozen at -20°C upon receiving, and used for DNA extraction as described below.

Patients were asked to maintain comprehensive food diaries in both studies; however, the two studies differed in dietary restrictions. The first protocol (COH IRB 16088) randomized patients receiving sunitinib to either a diet excluding yogurt or any bacterial fortified foods, or mandated that patients take a standard 4 ounces of a standard yogurt supplement (Activia™) twice daily for the 12-week study period. The second protocol (COH IRB 16323) mandated exclusion of yogurt or bacterial fortified foods for the 12-week study period.

At the end of the 12-week study period, the response was characterized using RECIST 1.1 criteria. For purposes of that study, responders were defined as patients achieving a complete response (CR), partial response (PR) or stable disease (SD) by 3 months of therapy, while non-responders were defined as those with progressive disease (PD) as the best response.

DNA was extracted from stool samples using a KingFisher MagMax Microbiome Ultra nucleic acid isolation kit (ThermoFisher, Waltham, MA, USA). Purified DNA was separated on 1% agarose gel and quantified via densitometry and spectrophotometry using a Qubit fluorometer assay (ThermoScientific). All samples were treated with RNase A before whole-metagenome library preparation. Sequencing libraries were generated using a KAPA Biosystems Hyper Prep kit and protocol (KK8504; Roche, Pleasanton, CA, USA). Libraries were then quantified using the KAPA Library Quantification kit (KR0405; Roche). Whole-metagenome libraries were pooled and sequenced on an Illumina NextSeq High Output platform ($2 \times$ 150 bp) with runs to generate at least 2 Gb per sample. Trimmomatic was used to remove adapters and low-quality bases and reads from demultiplexed reads .[8] Taxonomic profiling of trimmed metagenomic reads was performed using MetaPhlAn 2.0. [9,10] Hierarchical clustering by Bray-Curtis distance was performed using hclust2. Linear discriminant analysis (LDA) effect size (LEfSe) was used to identify significant differences in metagenomic taxa between patients who experienced clinical benefit or no clinical benefit [4]. The Shannon diversity index was used to quantify and compare microbial diversity across samples.

The structure of stool microbial community was compared between patients responding to nivolumab and nivolumab plus ipilimumab. Stool was collected from 31 patients before initiation of nivolumab (77%) or nivolumab plus ipilimumab (23%) therapy, of whom 58% experienced clinical benefit.[11] Additional stool collections were provided 1 month and 3 months after initiation of therapy. Bray-Curtis hierarchical cluster analysis revealed differences in microbiome profile between the clinical benefit and no clinical benefit groups. LEfSe identified 13 bacterial species with a linear discriminant analysis (LDA) score of $\geq$3 that distinguished between patients who experiences clinical benefit and those who did not using stool collected across all time points. Species with the greatest significant among patients with clinical benefit were *Bifidobacterium adolescentis* (p=0.002), *Barnesiella intestinihominis* (p=0.002), *Odoribacter splanchnicus* (p=0.006), and *Bacteroides*

eggerthii (p=0.009). Patients experiencing clinical benefit had greater alpha diversity according to the Shannon index (p=0.001) using stool collected across all time points. Among patients experiencing clinical benefit, the relative abundance of *Akkermansia* spp. generally increased across the CPI therapy timeline.

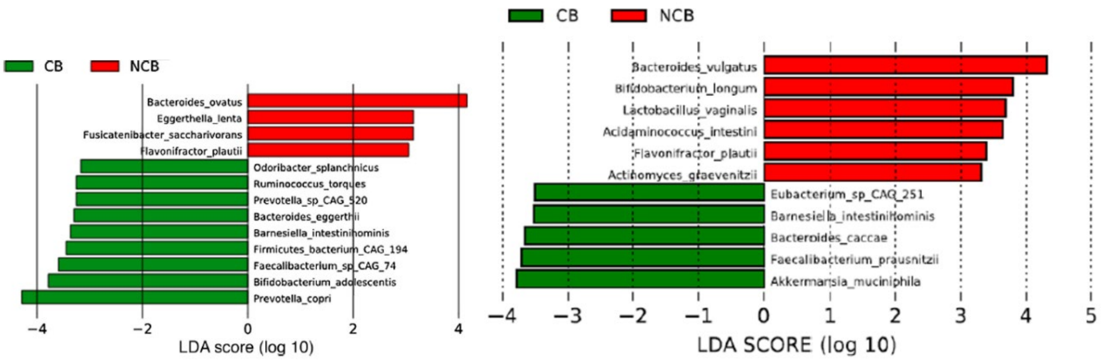

In another study, whole metagenome sequencing was performed on stool collected from all 20 patients, including specimens collected at baseline (prior to receipt of VEGF-TKI therapy) and at several sequential timepoints thereafter. With respect to the intervention, we detected *Bifidobacterium animalis* (the active ingredient of the probiotic yogurt product) in 67% of post-baseline specimens in patients in the probiotic supplemented group, as compared to 0.023% of post-baseline specimens in the probiotic restricted group[12]. Supplementation led to an increase in *Bifidobacterium* levels in post-baseline specimens. Of note was the presence of *Akkermansia muciniphila*, *Bacteroides caccae* and *Faecalibacterum prausnitzii* in the majority of samples from patients achieving clinical benefit. *Barnesiella intestinihominis*, a member of the family Porphyromondaceae, was almost exclusively present in those who achieved clinical benefit.

Linear discriminant effect size (LEfSe) identified 25 species that discriminated between the two study outcomes with an LDA score greater than three. *B. intestinihominis* was in this group and had the lowest p value (7.4 x 10-6) of all organisms in the clinical benefit category. Similarly, *Akkermansia muciniphila* was more abundant in the clinical benefit group (p=5.6 x 10-3).

2.3 CBM588

CBM588 is a strain of *Clostridium butyricum* isolated from the soil in Nagano, Japan in the 1960s. It has been widely used commercially in Japan as a live biotherapeutic in humans and a feed additive in animals. CBM588 was authorized by the European Union as a novel food ingredient in 2014, and as a feed additive for turkeys, chickens, and related minor avian species. [13] Previous studies have assessed the safety and tolerability of CBM588 in rats or dogs, and no toxicity was observed at dose levels of up to 5000 mg/kg for 12 months.[13] *In vivo* studies of CBM-588 have not identified adverse effects on reproduction or development. Furthermore, these studies showed that the agent promotes restoration of the GI microbiome by stimulating the growth of beneficial intestinal bacteria, including *Lactobacilli* and *Bifidobacteria*. Butyric acid is known to have immunomodulatory and anti-inflammatory effects on the intestinal epithelium. CBM588 has been demonstrated to significantly reduce ulceration and inflammation in a rat dextran sodium sulfate-induced colitis model. In a pediatric study including 110 children with upper respiratory tract infection or gastroenteritis, CBM588 was safe and well-tolerated. Furthermore, the incidence of antibiotic-related diarrhea was markedly reduced in patients who received CBM588 (59% vs 5%).[14] In a study of ulcerative colitis, CBM588 was administered at a dose of 60 mg oral tid. [15,16]

We have confidential data for the combination of nivolumab with ipilimumab with or without CBM-588. Although this study was done to assess biologic effect, we did see an appreciable difference in clinical outcome with CBM-588. In fact, the PFS for the nivolumab/ipilimumab plus CBM-588 arm was about 32 weeks longer than the arm for nivolumab/ipilimumab alone. While the nivolumab/ipilimumab plus CBM-588 arm hasn't reached a median OS, the nivolumab/ipilimumab arm has an OS of about 27 weeks. Additionally, CBM-588 supplementation led to an increase in *Bifidobacterium* levels in post-baseline specimen, compared to post-baseline specimens without CBM-588 supplementation. Clinically, *Bifidobacterium* is associated with some level of clinical benefit. [12]

Although the agent is commercially available in Japan, there is limited data to document the safety and tolerability of the combination of CBM588 with immunotherapy in patients with advanced cancer. We propose assessing CBM588 as an adjunct to cabozantinib/nivolumab in patients with mRCC in a phase I protocol. The OBED will represent the dose that results in the greatest increase in *Bifidobacterium* from baseline to week 12 of therapy. *Bifidobacterium* is selected amongst other putative bacteria that are associated with response to immunotherapy, since it is hypothesized that CBM588 will specifically increase levels of this genus.

3.0 ELIGIBILITY CRITERIA

Participants must meet all of the following criteria on screening examination to be eligible to participate in the study.

3.1 Inclusion Criteria

1. Histological confirmation of RCC with a clear-cell, papillary or sarcomatoid component.
2. Advanced (not amenable to curative surgery or radiation therapy) or metastatic (AJCC Stage IV) RCC.
3. No prior systemic therapy for RCC with the following exception:
   a. One prior adjuvant or neoadjuvant therapy for completely resectable RCC if recurrence occurred at least 6 months after the last dose of adjuvant or neoadjuvant therapy.
4. Measurable disease as per RECIST 1.1.
5. Recovery to baseline or ≤ Grade 1 CTCAE v5 from toxicities related to any prior treatments unless AE(s) are clinically nonsignificant and/or stable on supportive therapy.
6. Karnofsky performance status ≥ 70%
7. Males and females, ages ≥18.
8. Any ethnicity or race.
9. Adequate organ and marrow function, based upon meeting all of the following laboratory criteria within 14 days before first dose of study treatment:
   a. Absolute neutrophil count (ANC) ≥ 1500/µL without granulocyte colony-stimulating factor support.
   b. White blood cell count ≥ 2500/µL.
   c. Platelets ≥ 100,000/µL without transfusion.
   d. Hemoglobin ≥ 9 g/dL (≥ 90 g/L).
   e. Alanine aminotransferase (ALT), aspartate aminotransferase (AST), and alkaline phosphatase (ALP) ≤ 3 × upper limit of normal (ULN). ALP ≤ 5x ULN with documented bone metastases.
   f. Total bilirubin ≤ 1.5 × ULN (for subjects with Gilbert's disease ≤ 3x ULN).
   g. Serum albumin ≥ 2.8 g/dl.
   h. (PT)/INR or partial thromboplastin time (PTT) test < 1.3x the laboratory ULN
   i. Serum creatinine ≤ 1.5x ULN or calculated creatinine clearance ≥ 40mL/min (≥ 0.675mL/sec) using the Cockcroft-Gault equation:

Males: (140 – age) x weight (kg)/(serum creatinine [mg/dL] × 72)

Females: [(140 – age) x weight (kg)/(serum creatinine [mg/dL] × 72)] × 0.85

j. Urine protein/creatinine ratio (UPCR) ≤ 1 mg/mg (≤ 113.2 mg/mmol), or 24-h urine protein ≤ 1 g.

10. Capable of understanding and complying with the protocol requirements and must have signed the informed consent document.
11. Sexually active fertile subjects and their partners must agree to use medically accepted methods of contraception (e.g., barrier methods, including male condom, female condom, or diaphragm with spermicidal gel) during the course of the study and for 4 months after the last dose of cabozantinib, 5 months after the last dose of nivolumab for women with childbearing potential, and 7 months after the last dose of nivolumab for men.
12. Female subjects of childbearing potential must not be pregnant at screening. Female subjects are considered to be of childbearing potential unless one of the following criteria is met: documented permanent sterilization (hysterectomy, bilateral salpingectomy, or bilateral oophorectomy) or documented postmenopausal status (defined as 12 months of amenorrhea in a woman > 45 years-of-age in the absence of other biological or physiological causes. In addition, females < 55 years-of-age must have a serum follicle stimulating (FSH) level > 40 mIU/mL to confirm menopause). Note: Documentation may include review of medical records, medical examinations, or medical history interview by study site.

3.2 Exclusion Criteria:

1. Prior treatment with cabozantinib.
2. Current use, or intent to use, probiotics, yogurt, or bacterial fortified foods during the period of treatment.
3. Active interstitial lung disease (ILD)/pneumonitis or history of ILD/pneumonitis requiring treatment with systemic steroids.
4. Known medical condition (e.g., a condition associated with diarrhea or acute diverticulitis) that, in the investigator's opinion, would increase the risk associated with study participation or study drug administration or interfere with the interpretation of safety results.
5. Receipt of any type of small molecule kinase inhibitor (including investigational kinase inhibitor) within 2 weeks before first dose of study treatment.

6. Receipt of any type of cytotoxic, biologic, or other systemic anticancer therapy (including investigational) within 4 weeks before first dose of study treatment.
7. Radiation therapy for bone metastasis within 2 weeks or any other radiation therapy within 4 weeks before first dose of study treatment. Systemic treatment with radionuclides within 6 weeks before first dose of study treatment. Subjects with clinically relevant ongoing complications from prior radiation therapy are not eligible.
8. Known brain metastases or cranial epidural disease unless adequately treated with radiotherapy and/or surgery (including radiosurgery) and stable for at least 4 weeks prior to first dose of study treatment after radiotherapy or at least 4 weeks prior to first dose of study treatment after major surgery (e.g., removal or biopsy of brain metastasis). Subjects must have complete wound healing from major surgery or minor surgery before first dose of study treatment. Eligible subjects must be neurologically asymptomatic and without corticosteroid treatment at the time of first dose of study treatment.
9. Concomitant anticoagulation with coumarin agents (e.g., warfarin), direct thrombin inhibitors (e.g., dabigatran), direct factor Xa inhibitor betrixaban, or platelet inhibitors (e.g., clopidogrel). Allowed anticoagulants are the following:
    a. Prophylactic use of low-dose aspirin for cardio-protection (per local applicable guidelines) and low-dose low molecular weight heparins (LMWH).
    b. Therapeutic doses of LMWH or anticoagulation with direct factor Xa inhibitors rivaroxaban, edoxaban, or apixaban in subjects without known brain metastases who are on a stable dose of the anticoagulant for at least 1 week before first dose of study treatment without clinically significant hemorrhagic complications from the anticoagulation regimen or the tumor.
10. Administration of a live, attenuated vaccine within 30 days before first dose of study treatment.
11. The subject has uncontrolled, significant intercurrent or recent illness including, but not limited to, the following conditions:
    a. Cardiovascular disorders:
        i. Congestive heart failure New York Heart Association Class 3 or 4, unstable angina pectoris, serious cardiac arrhythmias.
        ii. Uncontrolled hypertension defined as sustained blood pressure (BP) > 140 mm Hg systolic or > 90 mm Hg diastolic despite optimal antihypertensive treatment.

iii. Stroke (including transient ischemic attack [TIA]), myocardial infarction (MI), or other ischemic event, or thromboembolic event (e.g., deep venous thrombosis, pulmonary embolism) within 6 months before first dose of study treatment.
    a. Subjects with a diagnosis of incidental, subsegmental PE or DVT within 6 months are allowed if stable, asymptomatic, and treated with a stable dose of permitted anticoagulation (see exclusion criterion #6) for at least 1 week before first dose of study treatment.

b. Gastrointestinal (GI) disorders including those associated with a high risk of perforation or fistula formation:
    i. The subject has evidence of tumor invading the GI tract, active peptic ulcer disease, inflammatory bowel disease (e.g., Crohn's disease), diverticulitis, cholecystitis, symptomatic cholangitis or appendicitis, acute pancreatitis, acute obstruction of the pancreatic duct or common bile duct, or gastric outlet obstruction.
    ii. Abdominal fistula, GI perforation, bowel obstruction, or intra-abdominal abscess within 6 months before first dose of study treatment. Note: Complete healing of an intra-abdominal abscess must be confirmed before first dose of study treatment.

12. Clinically significant hematuria, hematemesis, or hemoptysis of > 0.5 teaspoon (2.5 ml) of red blood, or other history of significant bleeding (e.g., pulmonary hemorrhage) within 12 weeks before first dose of study treatment.
13. Cavitating pulmonary lesion(s) or known endotracheal or endobronchial disease manifestation.
14. Lesions invading or encasing any major blood vessels.
15. Other clinically significant disorders that would preclude safe study participation.
    a. Any active, known, or suspected autoimmune disease will be excluded, with the following exceptions:
        i. Type 1 diabetes mellitus.
        ii. Hypothyroidism only requiring hormone replacement.
        iii. Skin disorders (e.g., vitiligo, psoriasis, or alopecia) not requiring systemic treatment.
        iv. Conditions not expected to recur in the absence of an external trigger.

b. Any condition requiring systemic treatment with either corticosteroids (> 10 mg daily prednisone equivalent) or other immunosuppressive medications within 14 days before first dose of study treatment.
    i. Note: Inhaled, intranasal, intra-articular, or topical steroids are permitted. Adrenal replacement steroid doses > 10 mg daily prednisone equivalent are permitted. Transient short-term use of systemic corticosteroids for allergic conditions (e.g., contrast allergy) is also allowed.
c. Active infection requiring systemic treatment. Acute or chronic hepatitis B or C infection, known human immunodeficiency virus (HIV) or acquired immunodeficiency syndrome (AIDS)-related illness, or known positive test for tuberculosis infection where there is clinical or radiographic evidence of active mycobacterial infection.
d. History of idiopathic pulmonary fibrosis, organizing pneumonia, drug-induced pneumonitis, idiopathic pneumonitis, or evidence of active pneumonitis on screening chest CT scan.
e. Serious non-healing wound/ulcer/bone fracture.
c. Malabsorption syndrome.
d. Uncompensated/symptomatic hypothyroidism.
e. Moderate to severe hepatic impairment (Child-Pugh B or C).
f. Requirement for hemodialysis or peritoneal dialysis.
g. History of solid organ or allogenic stem cell transplant.

16. Major surgery (e.g., laparoscopic nephrectomy, GI surgery, removal, or biopsy of brain metastasis) within 2 weeks before first dose of study treatment. Minor surgeries within 10 days before first dose of study treatment. Subjects must have complete wound healing from major surgery or minor surgery before first dose of study treatment. Subjects with clinically relevant ongoing complications from prior surgery are not eligible.
17. Corrected QT interval calculated by the Fridericia formula (QTcF) > 500 ms per electrocardiogram (ECG) within 14 days before first dose of study treatment. Furthermore, subjects with a history of additional risk factors for torsades de pointes (e.g., long QT syndrome) are also excluded.[17]
    Note: If a single ECG shows a QTcF with an absolute value > 500 ms, two additional ECGs at intervals of approximately 3 min must be performed within 30 min after the initial ECG, and the average of these three consecutive results for QTcF will be used to determine eligibility.

18. Pregnant or lactating females.
19. Inability to swallow tablets or unwillingness or inability to receive IV administration.
20. Previously identified allergy or hypersensitivity to components of the study treatment formulations or history of severe infusion-related reactions to monoclonal antibodies. Subjects with rare hereditary problems of galactose intolerance, the Lapp lactase deficiency or glucose-galactose malabsorption are also excluded.
21. Any other active malignancy at time of first dose of study treatment or diagnosis of another malignancy within 3 years prior to first dose of study treatment that requires active treatment, except for locally curable cancers that have been apparently cured, such as basal or squamous cell skin cancer, superficial bladder cancer, or carcinoma in situ of the prostate, cervix, or breast.

4.0 PARTICIPANT RECRUITMENT AND ENROLLMENT

This trial will be conducted as a single-center site in the City of Hope Comprehensive Cancer Center.

Eligible patients will be enrolled on study at the City of Hope Comprehensive Cancer Center by the Study Coordinator.

To register a patient, the eligibility packet should be completed by the research nurse/coordinator and sent to the data coordinating center (DCC) at DL-dcc@coh.org for review. DCC requires a 24-hour window for review of complete eligibility packets. Once complete, DCC will confirm eligibility and provide randomization to study arm, along with a confirmation of registration form and DCC signed checklist.

5.0 STUDY ASSESSMENTS AND PROCEDURES

5.1 Pre-treatment Period

During the Pre-Treatment Period, subjects are consented and qualified (screened) for the study. Informed consent must be obtained before initiation of any clinical screening procedure that is performed solely for the purpose of determining eligibility for this study. Evaluations performed as part of routine care before informed consent can be considered as screening evaluations if done within the defined screening period.

Study eligibility is based on meeting all of the study inclusion criteria and none of the exclusion criteria at screening and on Study Day 1 before study treatment administration. The following assessments will be conducted before subjects receive their first dose of treatment on this protocol:

1. CT Chest/Abdomen/Pelvis

2. Bone scan
3. MRI or CT of brain
4. Physical examination
5. Safety laboratory assessments
6. Participant orientation
7. Planned stool specimens

For each subject, the Pre-Treatment Period ends upon receipt of the first dose of study treatment or final determination that the subject is ineligible for the study.

5.2 Treatment Program Overview

This is a Phase I study and will consist of a randomization to one of two treatment arms to ascertain the biologic effect of cabozantinib/nivolumab with CBM588. Please refer to Statistical Analysis for details of randomization. Dose levels for each treatment arm are noted below:

Treatment Arms

Dose Level	Sample Size	Dose		
		CBM588 All Cycles	Cabozantinib All Cycles	Nivolumab All Cycles
**Arm 1**	**10**	None	40 mg oral daily	480 mg IV every 4 weeks
**Arm 2**	**20**	80 mg bid	40 mg oral daily	480 mg IV every 4 weeks

5.3 Cycle Definition

One cycle of therapy will constitute an every-4-week regimen of cabozantinib/nivolumab.

5.4 Treatment Plan

CBM588:

CBM588 will be administered orally on a daily basis, according to the allocated treatment arm per Section 5.2. Each dose to be taken twice daily (BID; am/pm), and at the same time each day or within a few hours of the regular time. Missed doses are allowed and should be documented on the study drug log. Emergency medications are per standard of care.

Nivolumab:

Nivolumab will be administered at a monthly dose of 480 mg. Emergency medications are per standard of care.

Cabozantinib:

Cabozantinib will be administered at a daily dose of 40 mg. Emergency medications are per standard of care. Missed doses should be documented in the medication diary.

5.5 Agent Administration

CBM588:

CBM588 will be supplied by Osel, Inc. CBM588 Fine Granules are manufactured by Mirayasan as an orally available live biotherapeutic comprised of *Clostridium butyricum* and packaged in 1 g sachets. Each sachet contains 40 mg of CBM588. CBM588 will be administered orally at a dose of 80 mg twice daily in 100 ml of water (the contents of two sachets) and should be given indefinitely while on protocol. Subjects can take CBM588 with or without food. CBM588 should be taken at home, not in clinic. The patients will be requested to maintain a study log, which includes a diet and stool frequency log, as well as a medication diary of each dose of medication. Patients who vomit CBM588 should skip the dosing for the day (they should not take another tablet/satchel in its place) and record the missed dose in the study medication diary. The study log will be returned to clinic staff at the end of each course.

Nivolumab

Nivolumab will be supplied by City of Hope Comprehensive Cancer Center and billed to patients and/or their third-party payer. Nivolumab injection is a clear opalescent, colorless to pale yellow, sterile, non-pyrogenic, single-use, isotonic aqueous solution formulated in sodium citrate, sodium chloride, mannitol, diethylenetriamine pentacetic acid (pentetic acid) and polysorbate 80 (Tween® 80), pH 6.0. Each vial is 100 mg (10 mg/mL) with a 0.7 mL overfill in 10 mL type I flint glass vials, with butyl rubber stoppers and aluminum seals. Vials of Nivolumab injection must be stored at 2°-8° C (36°F-46°F) and protected from light, freezing and shaking. If a storage temperature excursion is identified, promptly return Nivolumab to 2°C to 8°C and quarantine the supplies.

Nivolumab can be infused undiluted (10 mg/mL) or diluted with 0.9% Sodium Chloride injection, USP or 5% dextrose, USP to drug concentrations no less than 0.35 mg/mL. Note: mix gently and do not shake. Do **NOT** administer as IV push or bolus injection. Nivolumab injection is to be administered as a 30 minute IV infusion through a 0.2 micron to 1.2 micron pore size, low protein binding polyethersulfone membrane in-line filter. No compatibilities between nivolumab and polyvinyl chloride (PVC), nonPVC DHEP (di(2-ethylhexyl)phthalate) IV components, or glass bottles have been observed.

The administration of undiluted and diluted solutions of nivolumab must be completed within 24 hours preparation. If not used immediately, the infusion solution may be stored up to 24 hours in a refrigerator at 2°C-8°C (36°F-46°F) and a maximum of 4 hours of the total 24 hours can be at room temperature (20°C-25°C (68°F-77°F)) and room light. The maximum 4-hour period under room temperature and room light conditions includes the product administration period.

CAUTION: The single-use dosage form contains no antibacterial preservative or bacteriostatic agent. Therefore, it is advised that the product be discarded 8 hours after initial entry.

Cabozantinib

Cabozantinib will supplied by City of Hope Comprehensive Cancer Center and billed to patients and/or their third-party payer. The tablets are supplied as film coated tablets containing cabozantinib malate equivalent to 40 mg of cabozantinib and contain microcrystalline cellulose, lactose anhydrous, hydoxypropyl cellulose, croscarmellose sodium, colloidal silicon dioxide, magnesium stearate and Opadry® yellow. Cabozantinib must be taken orally and on an empty stomach, at a dose of 40 mg daily. Patients must fast for 2 hours before and 1 hour following each dose of cabozantinib. Any missed doses may not be taken within 12 hours of the next dose. The patient will be requested to maintain a medication diary of each dose of medication. Patients who vomit cabozantinib should skip the dosing for the day (they should not take another tablet/satchel in its place) and record the missed dose in the study medication diary. The medication diary will be returned to clinic staff at the end of each course. Instead of the medication diary, patients may use another preferred form (e.g. excel spreadsheet) to document that medication was taken.

The bottle of tablets must be stored at controlled room temperature, 20° to 25°C (68°F TO 77°F); temperature excursions are permitted between 15°C and 30°C (59°F to 86°F). Cabozantinib should be dispensed in its original container; however, cabozantinib tablets are stable up to 24 hours when dispensed in an open container, such as in a pill cup, and are stable for up to 7 days when dispensed in a closed container, such as a pharmacy dispensing bottle.

All oral self-administered investigational agents will be properly accounted for, handled, and disposed in accordance with existing federal regulations and principles of Good Clinical Practice.

**Regimen Description**					
**Agent**	**Premedications; Precautions**	**Dose**	**Route**	**Schedule**	**Cycle Length**
CBM588*	No prophylactic medication should be given.	80 mg	Oral	Twice per day indefinitely while on protocol.	28 days (4 weeks) for all cycles
Nivolumab	No prophylactic medication should be given unless indicated by previous indication.	480 mg	30 minutes IV infusion	Every four weeks indefinitely while on protocol	28 days (4 weeks) for all cycles
Cabozantinib*	No prophylactic medication should be given unless indicated by previous indication.	40 mg	Oral	Once per day indefinitely while on protocol.	28 days (4 weeks) for all cycles

- ***No sequence of administration is preferred for Cabozantinib and CBM588. However, these should be taken as specified in section 5.5 of the protocol.**

5.6 Assessments and Special Monitoring

For a detailed list of all study procedures including timing and windows, see Section 10.0.

Patients will be seen on day 1 of each cycle for toxicity assessment. For the purpose of this study, patients should be re-evaluated for response every 12 weeks. Response assessments include CT scan of the chest, abdomen, and pelvis and (if baseline bone disease is documented) bone scan. PET-CT or MRI may be substituted for CT if necessary, but evaluation with the imaging modality used at baseline is preferred.

Participants will be oriented to complete the daily study log, found in **Appendix C**. The log will be collected and reviewed during visits to the clinic at the end of each visit. The following information from the diary will be entered into the study specific database for analysis:

- Diarrhea categorized per CTCAE 5.0 diarrhea grading. Please note that the patient will be instructed to consider diarrhea to be an increase in stool frequency of < 4 over baseline. This definition is consistent with the CTCAE 5.0 terminology for grade 1 diarrhea.
- Stool specimen collection dates
- Yogurt intake, or intake of yogurt-containing foods
- Medication diary

5.7 Duration of Therapy and Criteria for Removal from Protocol Therapy

Participants may discontinue study treatment or withdraw their consent to participate in the study at any time without prejudice. The Investigator may withdraw a subject from study treatment or from the study if, in his or her clinical judgment, it is in the best interest of the subject or if the subject cannot comply with the protocol.

In addition, any of the following conditions requires discontinuation of the subject from study treatment:

- Completion of the protocol
- An AE or intercurrent illness that in the opinion of the Investigator warrants the subject's withdrawal from study treatment.
- The Investigator believes it is not in the best interest of the subject to continue on study.
- Specific conditions described in the Adverse Events and Unanticipated Problems Section 7.0.
- Necessity for treatment with other anticancer treatment prohibited by protocol.
- Sexually active subjects who refuse to use medically accepted barrier methods of contraception (e.g., male condom, female condom) during the study and for 4 months after the last dose of cabozantinib, 5 months after the last dose of nivolumab for women with childbearing potential, and 7 months after the last dose of nivolumab for men.
- Women who become pregnant or are breastfeeding.
    - If the subject does not recover from his or her toxicities to tolerable Grade ≤ 2 within 6 weeks, the subject will have study treatment discontinued unless there is unequivocal evidence that the subject is benefitting. In this situation, a subject may be able to restart therapy with a dose reduction upon resolution of the toxicity and with agreement of the Principal Investigator.
    - **Note:** If one agent is discontinued due to toxicity, then the participant may continue to receive the other study agents

- Necessity for interrupting all study treatment for greater than 12 weeks for study-treatment-related AEs unless approved by the Principal Investigator.
- Request by regulatory agencies for termination of treatment of an individual subject or all subjects under the protocol.
- Significant noncompliance with the protocol schedule in the opinion of the Investigator.
- Subjects who cannot tolerate the minimum protocol-specified dose of study treatment will have study treatment discontinued.
- Progressive disease (PD) or the subject no longer experiences clinical benefit as determined by the Investigator. Patients who continue study treatment beyond progression must be reconsented into the study.
- Subject participation in another clinical study using an investigational agent, investigational medical device, or other intervention.
- Subject request to discontinue all study treatments (with or without concurrent withdrawal of informed consent).
- Once participants meet criteria for removal from protocol therapy, the participant should then proceed to End of Treatment assessments, and then to follow-up.

Documentation of the reason for discontinuing protocol therapy and the date effective should be made in the Electronic Health Record/medical record and appropriate eCRF. The COH DCC and the Study PI should be promptly notified of the change in participant status.

5.8 Follow-Up

All participants will enter follow-up after completing End of Treatment assessments. This is comprised of:

- **Safety Follow-up**- 30 days post-last dose of protocol therapy.
    - **Note** the period for safety follow-up will be extended until stabilization or resolution for all reportable AEs (per the agreement of the Study PI) and accompanying follow-up safety report.
- **Response Follow-up**- for those who have yet to have disease progression.
- **Survival Follow-up**- for all participants who have progressed OR completed Active Response Follow-Up.

Assessment time points and windows are detailed in Section 10.0.

5.9 Duration of Study Participation

Study participation may conclude when any of the following occur:

- Completion of study activities.
- Withdrawal of consent.
- Participant is lost to follow-up. All attempts to contact the participant must be documented.
- At the discretion of the investigator for safety, behavioral, study termination or administrative reasons.

Documentation of the reason for discontinuing study participation and the date effective should be made in the Electronic Health Record/medical record and appropriate eCRF. The COH DCC should be promptly notified of the change in participant status.

5.10 Supportive Care, Prohibited and Concomitant Therapies/Medications

If concomitant therapy must be added or changed, including over-the-counter medications or alternative therapies, the reason and name of the agent/therapy should be recorded in the eCRF and documented in the Electronic Health Record/medical record.

5.10.1 Prohibited or restricted therapies

- Patients are asked not to take any other probiotic agents while on the current protocol. Steroid therapy refers to continuous use rather than tapered treatment.
- Given use of cabozantinib/nivolumab for augmentation of antitumor immunity, the following medications are prohibited during the study:
  - Immunosuppressive agents (except to treat a drug-related adverse event)
  - Systemic corticosteroids > 10 mg daily prednisone equivalent (except to treat a drug-related adverse event).

The following therapies are prohibited until study treatment has been permanently discontinued:

- Any investigational agent or investigational medical device.
- Oral anticoagulation with coumarin agents (e.g., warfarin), direct thrombin inhibitors (e.g., dabigatran), direct factor Xa inhibitor betrixaban, platelet inhibitors (e.g., clopidogrel), and chronic use of aspirin above low dose levels for cardio-protection per local applicable guidelines), until 4 weeks after cabozantinib has been permanently discontinued.
- Any non-protocol systemic anticancer treatment (e.g., chemotherapy, immunotherapy, radionuclides, drugs or herbal products used specifically for the treatment of the cancer under investigation).
- Immunosuppressive agents including immunosuppressive doses of systemic corticosteroids with exceptions as stated in Section 6.0.
- Live vaccines are prohibited while on study and until 5 months after last dose of nivolumab (e.g., intranasal influenza, measles, mumps, rubella, oral polio, BCG, yellow fever, varicella, and TY21a typhoid vaccines). The use of inactivated (killed) vaccines for the prevention of infectious disease is permitted.
- Metamizole (dipyrone) because of its potential for causing agranulocytosis.

The following therapies should be avoided until study treatment has been permanently discontinued or until otherwise specified:

- Local anticancer treatment including palliative radiation, ablation, embolization, or surgery with impact on tumor lesions should not be performed until radiographic progression per RECIST 1.1 has been established.
- Erythropoietic stimulating agents (e.g., epoetin alfa and darbepoetin alfa) should not be used based on a report of increased risk of tumor recurrence/progression associated with erythropoietin. [18]
- Concomitant medications that are known to prolong the QTc interval should be avoided in subjects who receive cabozantinib until they have permanently discontinued cabozantinib treatment (refer to http://www.qtdrugs.org for a list of drugs which have the potential to prolong the QTc interval).
- Chronic co-administration of cabozantinib with strong inducers of the CYP3A4 family (e.g., phenytoin, carbamazepine, rifampin, rifabutin, rifapentine, phenobarbital, and St. John's Wort) may significantly decrease cabozantinib concentrations and should be avoided. Selection of alternate concomitant medications with no or minimal CYP3A4 enzyme induction potential is recommended.
- Caution must be used when discontinuing treatment with a strong CYP3A4 inducer in a subject who has been concurrently receiving a stable dose of cabozantinib, as this could significantly increase the exposure to cabozantinib.
- Co-administration of cabozantinib with strong inhibitors of the CYP3A4 family (e.g., boceprevir, conivaptan, posaconazole, ketoconazole, itraconazole, clarithromycin, atazanavir, indinavir, nefazodone, nelfinavir, saquinavir, ritonavir, lopinavir, telaprevir, telithromycin, and voriconazole) may increase cabozantinib concentrations and should be avoided. Grapefruit, star fruit, and Seville oranges may also increase plasma concentrations of cabozantinib and should be avoided.

5.10.2 Supportive care

With the exception of prohibited therapies, participants should receive prophylactic or supportive as clinically indicated per institutional policies.

6.0 DOSE MODIFICATION/ DELAY GUIDELINES

6.1 CBM588

If treatment with cabozantinib and/or nivolumab is deferred, CBM588 should continue irrespective of dose delays or holds to cabozantinib/nivolumab. Dose modification or delays will not be permitted. If clinically significant non-hematologic adverse events occur (grade 3 or above, or persistent grade 2 toxicity at the investigators discretion) that are attributed to CBM588, then the agent should be discontinued.

6.2 Nivolumab in combination with cabozantinib

The allowed treatment window for administration of nivolumab and cabozantinib is +/- 7 days from Day 1 of the current cycle. Dose modifications for nivolumab in combination with cabozantinib by adverse event type are consistent with nivolumab and cabozantinib package inserts. General considerations for dose modifications are as follows.

For the initial induction period, patients with Grade 2 or 3 events requiring discontinuation of treatment with the combination may consider continuing treatment with single agent nivolumab or cabozantinib when the event resolves to baseline. However, patients with renal, CNS, or pulmonary toxicity must be removed from study.

- In addition to the adverse events identified in the table below, cabozantinib and nivolumab dose should be delayed for any adverse event, laboratory abnormality or inter-current illness which, in the judgment of the treating investigator, warrants delaying the dose of study medication
- Patients requiring a delay of >12 weeks or who experience immune-related toxicity with inability to decrease prednisone ≤10mg per oral daily must go off protocol therapy entirely.
- Patients who received systemic corticosteroids for continuous management of any drug-related immunologic toxicity must be off protocol.
- If a patient experiences several adverse events and there are conflicting recommendations, the investigator should use the recommended dose adjustment that reduces the dose to the lowest level.

6.2.1 Management of AEs Associated with Cabozantinib

The following should be taken into consideration in decisions regarding dose modifications (reductions or interruption):

- As a general approach all AEs should be managed with supportive care at the earliest signs of toxicity considered related to the study treatment. Should this be ineffective, dose interruptions and/or reductions should be considered to prevent worsening of toxicity.
- The assigned starting dose for cabozantinib is 40 mg/day. 20 mg/day dose reduction levels of cabozantinib are permitted (see "Dose Reductions of Cabozantinib" table).

- Dose modification criteria for cabozantinib are shown in the “Dose Reductions of Cabozantinib” table. Dose interruptions and/or reductions should be implemented for unacceptable toxicity. Doses may be modified at any time while a subject is on treatment.
- Dose reductions or interruptions may also occur in the setting of lower grade toxicity than defined in the “Dose Modifications of Cabozantinib for Treatment-Related AEs” table , if the Investigator feels it is in the interest of a subject’s safety and will optimize drug tolerability.
- Interruption of cabozantinib treatment for cabozantinib-related AEs may occur at any time per Investigator discretion. If treatment is interrupted due to related AEs for more than 12 weeks, cabozantinib should be discontinued unless there is unequivocal evidence that the subject is benefitting. In this situation, a subject may be able to restart therapy with a dose reduction upon resolution of the toxicity, with approval from the Principal Investigator.
- Dose interruptions for reason(s) other than related AEs (e.g., surgical procedures) can be longer than 12 weeks per the discretion of the Investigator.
- Guidelines for the management of specific AEs of cabozantinib such as GI disorders, non-GI fistula formation, hemorrhage, thromboembolic events, hypertension, stomatitis and mucositis, skin disorders, osteonecrosis, proteinuria, nervous system disorders, infections and infestations, blood system disorders, fatigue, weight loss, QTc prolongation, electrolyte disorders, endocrine disorders, and respiratory disorders are provided. Guidelines for the management of diarrhea/colitis and guidelines for the management of hepatic events are also included.

Dose Reductions of Cabozantinib

Assigned Dose	First Dose Level Reduction	Second Dose Level Reduction	Third Dose Level Reduction
40 mg daily (qd)	20 mg daily (qd)	20 mg every other day (qod)	No dose reduction permitted
Cabozantinib will be discontinued if a dose of 20-mg cabozantinib every other day (minimum dose) is not tolerated			

Dose Modifications of Cabozantinib for Treatment-Related AEs

Event	Management
Grade 1 AEs	Add supportive care as indicated. Continue cabozantinib treatment at the current dose level if AE is manageable and tolerable.
Grade 2 AEs which are tolerable and are easily managed	Continue cabozantinib treatment at the current dose level with supportive care.
Grade 2 AEs which are **intolerable** and **cannot be adequately managed**	At the discretion of the Investigator, cabozantinib should be dose reduced or interrupted.<br><br>Note: It is recommended that dose holds be as brief as possible.
Grade 3 AEs (except clinically non-relevant laboratory abnormalities)	Cabozantinib should be interrupted unless the toxicity can be easily managed with a dose reduction and optimal medical care.<br><br>Note: It is recommended that dose holds be as brief as possible.
Grade 4 AEs (except clinically non-relevant laboratory abnormalities)	Subjects should have cabozantinib interrupted immediately. Discontinue cabozantinib unless the following criteria are met:<br>• Subject is deriving clear clinical benefit as determined by the Investigator and approved by the Principal Investigator.<br>• Toxicity can be managed with a dose reduction following recovery to Grade 1 (or baseline) and optimal medical care.
Note: The dose delay and modification criteria for specific medical conditions are provided.	

6.2.1.1 Cabozantinib Dose Reinstitution and Reescalation

- If the subject recovers from his or her toxicities to CTCAE v.5.0 Grade ≤ 1 or to the baseline value (or lower) and the toxicity was unrelated to cabozantinib, then cabozantinib may be restarted with no change in dose.
- If the subject recovers from his or her toxicities to Grade ≤ 1 or to the baseline value (or lower) the toxicity was deemed possibly related to cabozantinib, then cabozantinib may be restarted at a reduced dose.
- Subjects who initiated treatment with cabozantinib at 40 mg and experience a possibly related AE of Grade 1 or 2 severity may be restarted with no dose change after recovery of the toxicities to ≤ Grade 1 or to the baseline value (or lower) if appropriate supportive care can prevent or minimize the risk of the AE.

Re-escalation to the previous dose, (but not higher than 40 mg/day) may be allowed at the discretion of the Investigator for AEs which have resolved or recovered to Grade 1 (or baseline value) and deemed tolerable and easily managed by optimized supportive treatment. A minimum two-week interval is needed between resuming study treatment and the escalation to the next higher dose level. Dose

re-escalation is not allowed for a drug-related dose reduction triggered by Grade 4 hematologic toxicities or by Grade 4 AEs affecting major organs (e.g., central nervous system, cardiac, hepatic, renal).

6.2.1.2 Guidelines for Management of Potential Adverse Events Associated with Cabozantinib Treatment

Subjects will be monitored for AEs from the time of signing informed consent through their last follow-up visit (30 days after the date of the last dose of cabozantinib treatment.) Subjects will be instructed to notify their physician immediately at the onset of any AE. Seriousness, severity grade, and relationship to study treatment of AEs will be determined by the Investigator. AE severity will be graded by the Investigator in accordance with CTCAE v.5.0.

Management of severe or intolerable adverse reactions may require temporary dose reduction and/or interruption for cabozantinib.

The most frequent AEs experienced by ≥ 20% of subjects treated with cabozantinib in descending order of frequency were diarrhea, fatigue, decreased appetite, nausea, palmar-plantar erythodysesthesia syndrome (PPES), vomiting, weight decreased, hypertension, PPES, vomiting, constipation, hypertension, dysgeusia, dysphonia, and asthenia. For a full description of the safety profile of cabozantinib, refer to the cabozantinib Investigator's Brochure.

Subjects may also experience other medically important but less frequent AEs including arterial and venous thrombotic AEs (e.g., deep vein thrombosis [DVT], pulmonary embolism, transient ischemic attack [TIA], and myocardial infarction [MI]), severe hemorrhagic events, proteinuria, wound healing complications, GI perforation, abscesses including intra-abdominal and pelvic abscess, GI and non-GI fistulae formation, osteonecrosis, and RPLS).

Adverse events associated with laboratory abnormalities experienced by ≥ 5% of subjects treated with cabozantinib in descending order of frequency were anemia, AST increased, ALT increased, hypothyroidism, hypokalemia, hypomagnesemia, thrombocytopenia, hypocalcemia, hypophosphatemia, LDH increased, lipase increased, neutropenia, ALP increased, hyponatremia, and leukopenia.

Many AEs can occur early (within the first few weeks) in the course of treatment with cabozantinib, as cabozantinib is expected to reach steady state exposure at approximately 2 weeks following first dose. Events that generally have an early onset include hypocalcemia, hypokalemia, thrombocytopenia, hypertension, PPES, abdominal pain, mucosal inflammation, constipation, diarrhea and vomiting. In

addition, earlier onset for events of dehydration was observed in subjects with castrate-resistant prostate cancer (CRPC) when compared with subjects with other tumor types.

Adverse events should be managed with supportive care at the earliest signs of toxicity. Dose reductions and treatment interruptions should be considered. Dose reductions are recommended for events that, if persistent, could become serious or intolerable. Cabozantinib should be discontinued for the following AEs: visceral perforation or fistula formation, severe hemorrhage, serious arterial thromboembolic events, nephrotic syndrome, hypertensive emergency, persistent uncontrolled hypertension despite optimal medical management, and reversible posterior leukoencephalopathy syndrome (RPLS).

A population pharmacokinetics analysis of cancer subjects with RCC, HCC, MTC, glioblastoma, and other solid tumors predicted a terminal half-life of approximately 99 h for cabozantinib when administered as repeated once-daily doses. Thus, as described above, it will take most subjects 2 to 3 weeks (5 half-lives) to reach steady state with daily dosing after initiating therapy with cabozantinib. AEs attributable to cabozantinib may be expected to occur by the time maximum plasma concentrations are reached, and therefore early intervention with dose modifications within the first 15-21 days of daily dosing may be justified.

Gastrointestinal Events

<u>Diarrhea</u>: See Section 6.2.3 for guidance regarding both cabozantinib and nivolumab.

<u>Gastrointestinal perforation, GI fistula, and intra-abdominal and pelvic abscess</u>:

Prior to initiation of treatment with cabozantinib, subjects should be carefully evaluated for potential risk factors including (but not limited to) the following:

- Tumors invading GI or respiratory tracts.
- Active peptic ulcer disease, inflammatory bowel disease (e.g., ulcerative colitis, Crohn's disease), diverticulitis, cholecystitis or symptomatic cholangitis, or appendicitis.
- History of abdominal fistula, GI perforation, bowel obstruction, or intra-abdominal abscess.
- Ongoing visceral complications from prior radiation therapy.
- Prior GI surgery (particularly when associated with delayed or incomplete healing).

Complete healing following abdominal surgery and radiation therapy and/or resolution of intra-abdominal abscess must be confirmed prior to initiating treatment with cabozantinib.

After starting cabozantinib, subjects should be monitored for early signs of GI perforation such as abdominal pain, nausea, emesis, constipation, and fever especially if known risk factors for developing GI perforation or fistula (Turnage et al. 2008) are present.

Discontinue cabozantinib and initiate appropriate management in subjects who have been diagnosed with GI perforation or fistula.
Nausea and vomiting: Antiemetic agents are recommended as clinically appropriate for treatment or prophylaxis of nausea and vomiting, along with supportive care. Dehydration and electrolyte abnormalities may be associated with vomiting and monitoring for and correction of fluid and electrolyte disturbances should be implemented. Antiemetic medications should be assessed for potential drug interactions.

Hepatic Events

See section 6.2.4 for guidance regarding both cabozantinib and nivolumab.

Non-Gastrointestinal Fistula

Radiation therapy (in certain areas of the body) has been identified as a possible predisposing risk factor for fistula formation in subjects undergoing treatment with cabozantinib.

Subjects with any clinically relevant ongoing complications from prior radiation therapy (e.g., radiation esophagitis or other inflammation of the viscera) should not be treated with cabozantinib until these complications have resolved.

Radiation therapy to the thoracic cavity (including mediastinum) should be avoided within 4 weeks of starting treatment with cabozantinib (excluding local radiation for bone metastases). Fistula should be ruled out as appropriate in cases of onset of severe mucositis or difficulty swallowing after start of therapy. Discontinue cabozantinib and initiate appropriate management in subjects who have been diagnosed with a non-GI fistula.

Hemorrhage

Hemorrhagic events, including serious and sometimes fatal events, have been reported with cabozantinib. Subjects should be evaluated for potential bleeding risk factors prior to initiating

cabozantinib treatment and should be monitored for bleeding events with serial complete blood counts and physical examination while on study. Risk factors for hemorrhagic events may include (but may not be limited to) the following:

- Tumor of the lung with cavitary lesions or tumor lesions which invade or encase major blood vessels. NSCLC with squamous cell differentiation is known for significant lung cavitations and centrally located tumors that may invade major blood vessels. Thus, the anatomic location and characteristics of tumor as well as the medical history should be carefully reviewed in the selection of subjects for treatment with cabozantinib.
- Recent or concurrent radiation to the thoracic cavity.
- Active peptic ulcer disease, inflammatory GI diseases including Crohn's disease and ulcerative colitis.
- Underlying medical conditions which affect normal hemostasis (e.g., deficiencies in clotting factors and/or platelet function, or thrombocytopenia).
- Concomitant medication with anticoagulants or other drugs which affect normal hemostasis.
- History of clinically significant hemoptysis, hematemesis, or hematuria.

The risk of hemorrhage in cabozantinib-treated subjects with brain metastases has not been thoroughly analyzed. Though the incidence of CNS hemorrhage events in a study of subjects with glioblastoma was higher than observed in general population of subjects with cancer treated with cabozantinib, it is not clear how the risk of hemorrhage in glioblastoma translates to a risk of hemorrhage for subjects with brain metastases. Currently, brain metastases of carcinomas are not contraindications to the use of cabozantinib, but subjects with brain metastases should be monitored with a high index of suspicion if symptoms that could be due to a CNS hemorrhage occur.

Complete healing from radiation-induced side effects should have occurred before initiating cabozantinib treatment, and cabozantinib should be discontinued in subjects with serious and life-threatening bleeding events or recent hemoptysis (≥ 2.5 mL of red blood).

Vascular Disorders

Thromboembolic Events

Thromboembolic events are frequent in cancer subjects due to procoagulant changes induced by the malignancy or anticancer therapy. DVT and pulmonary embolism have been observed in clinical studies with cabozantinib, including fatal events. Subjects who develop a pulmonary embolism and/or DVT should have study treatment interrupted until therapeutic anticoagulation is established. Treatment with cabozantinib may be resumed in subjects with pulmonary embolism or DVT if it is determined that the event is uncomplicated and that the subject is deriving clinical benefit from cabozantinib treatment and that anticoagulation does not place them at a significant risk that outweighs the benefit of resuming treatment per discretion of the Investigator and according to individual protocols. Therapeutic doses of LMWH or the direct factor Xa oral inhibitors rivaroxaban, edoxaban, or apixaban are allowed for management of thrombotic events. Other oral anticoagulants including coumarin agents (e.g., warfarin), direct thrombin inhibitors (e.g., dabigatran), direct factor Xa inhibitor betrixaban, platelet inhibitors (e.g., clopidogrel), and chronic use of aspirin above low dose levels for cardioprotection per local applicable guidelines are not allowed, until 4 weeks after cabozantinib has been permanently discontinued.

Arterial thrombotic events (e.g., TIA, MI) have been observed in studies with cabozantinib. Subjects should be evaluated for pre-existing risk factors for arterial thrombotic events such as diabetes mellitus, hyperlipidemia, hypertension, coronary artery disease, history of tobacco use, and cardiac and/or thromboembolic events that occurred prior to initiation of study treatment. Further treatment with cabozantinib should be discontinued in subjects who develop an acute MI, cerebral infarction, or any other clinically significant arterial thromboembolic complication.

Hypertension

The below table provides treatment guidelines for hypertension deemed related to cabozantinib. Subjects with known hypertension should be optimally managed prior to entry into clinical trials with cabozantinib according to entry criteria of specific protocols. Blood pressure should be monitored in a constant position visit to visit, either sitting or supine in a relaxed setting. Decisions to reduce or interrupt the dose of study treatment must be based on BP readings taken by a medical professional and must be confirmed with a second measurement at least 5 minutes following the first measurement.

Cabozantinib should be discontinued in subjects with hypertensive emergency.

Management of Hypertension Associated with Cabozantinib

Event	Management
**Subjects NOT receiving optimized anti-hypertensive therapy**	
> 140 mm Hg (systolic)[a] and < 160 mm Hg<br><br>OR<br><br>> 90 mm Hg (diastolic) and < 110 mm Hg	• Optimize antihypertensive medications by adding new or additional antihypertensive medications and/or increase dose of existing medications.<br>• Reduce cabozantinib treatment by one dose level if optimal antihypertensive therapy (usually to include 3 agents) does not result in BP <140 mm Hg systolic or <90 mm Hg diastolic.<br>• If subject is symptomatic interrupt cabozantinib treatment
≥ 160 mm Hg (systolic)<br><br>OR<br><br>≥ 110 mm Hg (diastolic)	• Reduce cabozantinib by one dose level[b] or interrupt cabozantinib treatment per Investigator discretion.<br>• Add new or additional anti-hypertensive medications and/or increase dose of existing medications and monitor subject closely for hypotension. If optimized antihypertensive therapy (usually to include 3 agents) does not result in BP < 140 mm Hg systolic or < 90 mm Hg diastolic, cabozantinib treatment should be dose reduced further or interrupted.<br>• Cabozantinib treatment should be dose interrupted if upper limits of systolic BP (≥ 160 mm Hg) are sustained and not adequately manageable or if systolic BP is > 180 mm Hg or diastolic BP > 110 mm Hg, or if subject is symptomatic.<br>• Re-start cabozantinib treatment at the most tolerable dose and re-escalate only if BP falls to and is sustained at < 140 mm Hg systolic and < 90 mm Hg diastolic.
Hypertensive emergency[c]	• Discontinue cabozantinib treatment.
[a] Permitted dose levels are defined by individual protocols.<br>[b] Hypertensive emergency is defined as uncontrolled elevated BP with clinical evidence of progressive or impending end-organ damage (e.g., myocardial infarction/ischemia, intracranial hemorrhage, cerebral ischemia, pulmonary edema, encephalopathy, kidney damage).	

Stomatitis and Mucositis

Preventive measures may include a comprehensive oral examination to identify and treat any potential risk for complications before study treatment is initiated. Appropriate correction of local factors should be instituted as indicated, such as modification of ill-fitting dentures and appropriate care of gingivitis. During treatment with cabozantinib, good oral hygiene and standard local treatments such as non-traumatic and non-irritating cleansing, and oral rinses (e.g., with a weak solution of salt and baking

soda) should be maintained. Lips should be kept moisturized with lip balm. The use of lipstick, lip-gloss, and Vaseline should be avoided.
Local treatment should be instituted at the earliest onset of symptoms. Obtain bacterial/viral culture if oral infection is suspected and treat infection as clinically indicated.

Skin and Subcutaneous Tissue Disorders

Wound healing and surgery: Cabozantinib has the potential to cause wound healing complications and wound dehiscence which may even occur long after a wound has been considered healed. Therefore, surgical and traumatic wounds must not only be completely healed prior to starting cabozantinib treatment but must also be monitored for wound dehiscence, wound infection and other signs of impaired wound healing while the subject is being treated with cabozantinib. If dehiscence occurs, cabozantinib treatment should not be restarted until complete healing has taken place.

Treatment with cabozantinib should be stopped at least 3 weeks prior to elective surgery. Do not administer cabozantinib for at least 2 weeks after major surgery and until complete wound healing.

Palmar-plantar erythrodysesthesia syndrome (PPES; also known as hand-foot syndrome), skin rash (including blister, erythematous rash, macular rash, skin exfoliation, dermatitis acneiform, and papular rash), pruritus, dry skin, erythema, pigmentary changes, and alopecia have been reported with cabozantinib. All subjects on study should be advised on prophylactic measures including the use of emollients, removal of calluses, avoidance of exposure of hands and feet to hot water leading to vasodilatation, protection of pressure-sensitive areas of hands and feet, and use of cotton gloves and socks to prevent injury and keep the palms and soles dry.

Early manifestations include tingling, numbness, mild hyperkeratosis, and symmetrical red and swollen areas on the palms and soles. The lateral sides of the fingers or periungual zones may also be affected. Adequate interventions are required to prevent worsening of skin symptoms such as blisters, desquamations, ulcerations, or necrosis of affected areas. Analgesics may be required for pain control.

Aggressive management of symptoms is recommended, including early dermatology referral.

Management of Hand-Foot Syndrome (PPES) Associated with Cabozantinib

Event	Management
Grade 1	Cabozantinib treatment may be continued at the current dose if PPES is clinically insignificant and tolerable. Otherwise, cabozantinib should be reduced to the next lower dose level.[a] Start urea 20% cream twice daily AND clobetasol 0.05% cream once daily. Reassess at least weekly; if PPES worsens at any time or does not improve after 2 weeks, proceed to the intervention guidelines for Grade 2.
Grade 2	Cabozantinib treatment may be continued if PPES is tolerated. Cabozantinib should be dose reduced or interrupted if PPES is intolerable. Continue urea 20% cream twice daily AND high potency steroid cream (e.g., clobetasol 0.05%) once daily and add analgesics (e.g., NSAIDs/gamma-aminobutyric acid agonists) for pain control if needed. Reassess at least weekly; if PPES worsens or affects self-care, proceed to the intervention guidelines for Grade 3.
Grade 3	Interrupt cabozantinib treatment until severity decreases to Grade 1 or 0. Continue treatment of skin reaction with high potency steroid cream (e.g., clobetasol 0.05%) twice daily AND analgesics. Resume cabozantinib at a reduced dose if PPES recovers to Grade ≤ 1. Discontinue subject from cabozantinib if PPES does not improve within 6 weeks.
[a] Permitted dose levels are defined by individual protocols.	

Angioedema

Angioedema should be managed according to standard practice. The subject should be observed until symptoms resolve, with particular attention to maintaining an open airway.

Osteonecrosis

Osteonecrosis has been reported in subjects treated with cabozantinib. Additional risk factors include use of bisphosphonates and denosumab, chemotherapy and anti-angiogenic drugs, use of corticosteroids, local radiotherapy, and dental or orofacial surgery procedures.

Osteonecrosis of the jaw (ONJ) can manifest as jaw pain, osteomyelitis, osteitis, bone erosion, tooth or periodontal infection, toothache, gingival ulceration, or gingival erosion. Persistent pain or slow healing of the mouth or jaw after dental surgery may also be manifestations of osteonecrosis.

Perform an oral examination prior to initiation of cabozantinib and periodically during cabozantinib treatment. Advise subjects regarding oral hygiene practice and to quickly report symptoms to Investigator. Caution should be used in subjects receiving bisphosphonates.

Invasive dental procedures should be avoided. In cases where dental procedures are unavoidable, treatment with cabozantinib should be interrupted for at least 3 weeks prior to the procedure and resumed after complete wound healing has occurred. Bone healing may often require a protracted time.

Withhold cabozantinib for development of ONJ until complete resolution.

Proteinuria

Proteinuria has been reported with cabozantinib. Proteinuria should be monitored by measuring UPCR. The below table provides treatment guidelines for proteinuria deemed related to cabozantinib. Cabozantinib should be discontinued in subjects who develop nephrotic syndrome (proteinuria > 3.5 grams per day in combination with low blood protein levels, high cholesterol levels, high triglyceride levels, and edema).

Management of Proteinuria Associated with Cabozantinib

Event	Management
UPCR ≤ 1 mg/mg<br>(≤ 113.1 mg/mmol)	• No change in cabozantinib treatment or monitoring.
UPCR > 1 and < 3.5 mg/mg<br>(> 113.1 and < 395.9 mg/mmol)	• Consider confirming with a 24-h protein assessment within 7 days.<br>• No change in cabozantinib treatment required if UPCR ≤ 2 mg/mg or urine protein ≤ 2 g/24 h on 24-h urine collection.<br>• Dose reduce or interrupt cabozantinib treatment if UPCR > 2 mg/mg on repeat UPCR testing or urine protein > 2 g/24 h on 24-h urine collection. Continue cabozantinib on a reduced dose if UPCR decreases to < 2 mg/mg. Consider interrupting cabozantinib treatment if UPCR remains > 2 mg/mg despite a dose reduction until UPCR decreases to < 2 mg/mg. Restart cabozantinib treatment at a reduced dose after a dose interruption.<br>• Repeat UPCR within 7 days and once per week. If UPCR < 1 mg/mg on 2 consecutive readings, UPCR monitoring can revert to protocol-specific times. (Second reading is confirmatory and can be done within 1 week of first reading.) If UPCR remains > 1 mg/mg and < 2 mg/mg for 1 month or is determined to be stable (< 20% change) for 1 month, check urine protein/creatinine per protocol or as clinically indicated.
UPCR ≥ 3.5 mg/mg<br>(≥ 395.9 mg/mmol)	• Interrupt cabozantinib treatment pending repeat UPCR within 7 days and/or 24-h urine protein.<br>• If ≥ 3.5 mg/mg on repeat UPCR, continue to hold cabozantinib treatment and check UPCR every 7 days. If UPCR decreases to < 2 mg/mg, restart cabozantinib treatment at a reduced dose and monitoring of urine protein/creatinine should continue weekly until the UPCR decreases to < 1 mg/mg. If UPCR remains > 1 mg/mg and < 2 mg/mg for 1 month or is determined to be stable (< 20% change) for 1 month, check urine protein/creatinine per protocol or as clinically indicated.
Nephrotic syndrome	• Discontinue cabozantinib treatment.

Nervous System Disorders

Cabozantinib appears to represent minimal risk of adverse neurological effects based on nonclinical Good Laboratory Practice (GLP)-compliant toxicology studies. Dysphonia, dysgeusia, headache, dizziness, confusional state, convulsion, depression, memory impairment, hypoesthesia, peripheral neuropathy, insomnia, ataxia, and encephalopathy have been observed in clinical studies with cabozantinib. The development of any new or progressive, unexplained neurological symptoms should be assessed for underlying causes. RPLS has been reported.

RPLS should be considered in any subject presenting with seizures, headache, visual disturbances, confusion or altered mental function. Cabozantinib treatment should be discontinued in subjects with RPLS.

Infections and Infestations

Infections are commonly observed in cancer subjects. Predisposing risk factors include a decreased immune status (e.g., after myelosuppressive anticancer therapies, splenectomy), destructive growth of the underlying malignancy including bone marrow infiltration with suppression of normal hematopoiesis, as well as the presence of IV devices.

Infections and abscesses should be treated with appropriate local care and systemic therapy. Cabozantinib should be interrupted until complete healing has taken place.

Blood and Lymphatic System Disorders

Hematological toxicities (i.e., neutropenia, thrombocytopenia, and anemia) and associated complications have been observed after administration of cabozantinib and may be managed with dose interruptions and/or dose reductions. Subjects with hematologic toxicities may require additional or more frequent laboratory tests according to institutional guidelines.

Dose reductions or dose interruptions for hematological toxicities are not mandated but can be applied as clinically indicated. Supportive care for thrombocytopenia or anemia, such as transfusions, may be managed according to institutional guidelines. The use of colony-stimulating growth factors should be considered unless inhibited by protocol. Febrile neutropenia or evidence of infection associated with neutropenia must be assessed immediately and treated appropriately and in a timely manner according to institutional guidelines.

Fatigue

Common causes of fatigue, such as anemia, deconditioning, emotional distress (depression and/or anxiety), poor nutrition, dehydration, sleep disturbance, and hypothyroidism should be ruled out and treated according to standard of care. Pharmacological management should be considered after disease specific morbidities have been excluded when not prohibited.

Weight Loss

Anorexia and weight loss should be managed according to local standard of care including nutritional support. Pharmacologic therapy should be considered for appetite enhancement when not prohibited by a particular protocol.

Corrected QT Prolongation

The effect of orally administered cabozantinib 140 mg qd on QTc interval was evaluated in a placebo-controlled study in subjects with MTC. A mean increase in QTcF of 10-15 ms was observed after 4 weeks after initiating cabozantinib treatment. A concentration-QTc relationship could not be definitively established. Changes in cardiac wave form morphology or new rhythms were not observed. No cabozantinib-treated subjects in this study had a QTcF > 500 ms. Review of the larger safety database (including post marketing data) confirmed the absence of safety concerns associated with QT prolongation. There were no events of torsades de pointes reported.

Unless otherwise specified, only subjects with a baseline QTcF ≤ 500 msec are eligible for cabozantinib research studies. Cabozantinib should be used with caution in subjects with QT prolongation risk, a history of QT interval prolongation, or who are taking antiarrhythmics or drugs known to prolong the QT interval. Concomitant treatment with strong CYP3A4 inhibitors, which may increase cabozantinib plasma concentrations, should be avoided.

If at any time on study there is an increase in QTcF to an absolute value > 500 ms or an increase of > 60 ms above baseline, two additional ECGs must be performed with intervals not less than 3 min apart within 30 min after the initial ECG.

If the average QTcF from the three ECGs is > 500 ms or increased by > 60 ms above baseline, the following actions must be taken:

- Interrupt cabozantinib treatment.
- Hospitalize symptomatic subjects (e.g., with palpitations, dizziness, syncope, orthostatic hypotension, a significant ventricular arrhythmia on ECG) for a thorough cardiology evaluation and management.
- Consider cardiology consultation for asymptomatic subjects for evaluation and management

- Check electrolytes, especially magnesium, potassium and calcium; correct abnormalities as clinically indicated.
- Check concomitant medications for any medication that may have contributed to QT prolongation, and if possible, discontinue these medications (http://www.qtdrugs.org).
- Repeat ECG triplicates hourly until the average QTcF is ≤ 500 ms and the average increase is ≤ 60 ms above baseline, or otherwise determined by consultation with a cardiologist or appropriate expert.

Subjects with QTc prolongation and symptoms must be monitored closely until the QTc elevation and symptoms have resolved. Cabozantinib treatment may be restarted at a reduced dose level if all of the following conditions are met:

- Symptoms are determined to be unrelated to the QT interval prolongation.
- Cabozantinib treatment has been interrupted through a minimum of 1 week following the return of the QTcF to ≤ 500 ms or return to ≤ 60 ms above baseline.
- QT prolongation can be unequivocally associated with an event other than cabozantinib administration and is treatable/has been resolved.
- Principal Investigator has reviewed all available information and has agreed to the continuation of study treatment

Following reinitiation of study treatment, ECGs must be repeated weekly for 2 weeks, then every 2 weeks for 1 month, then according to the protocol-defined time points.
Cabozantinib treatment must be permanently discontinued if either of the following applies:

- Cardiac evaluation confirms that symptoms are the consequence of QT interval prolongation.
- Recurrence of QTcF prolongation after reinitiation of study treatment at a reduced dose.

Endocrine Disorders

Treatment-emergent elevation of thyroid-stimulating hormone (TSH) has been observed with cabozantinib treatment. Currently available data are insufficient to determine the mechanism of thyroid function test alterations and its clinical relevance. Routine monitoring of thyroid function and assessments for signs and symptoms associated with thyroid dysfunction is recommended before initiation and during treatment with cabozantinib. Management of thyroid dysfunction (e.g., symptomatic hypothyroidism) should follow accepted clinical practice guidelines.

Musculoskeletal and Connective Tissue Disorders

Cabozantinib appears to represent minimal risk of adverse musculoskeletal effects based on nonclinical GLP-compliant toxicology studies. The development of new or progressive, unexplained musculoskeletal symptoms such as pain or weakness should be assessed for underlying causes.

Rhabdomyolysis has been reported. Cabozantinib should be discontinued in subjects with serious and life-threatening rhabdomyolysis and interrupted if less severe forms occur when there are no other clear causes. Reinitiation of cabozantinib treatment must be discussed with and approved by the Principal Investigator. Therapy of rhabdomyolysis should include supportive care and standard medical intervention.

Respiratory, Thoracic and Mediastinal Disorders

Dyspnea has been reported in clinical studies with cabozantinib. Symptoms should be managed according to locally accepted clinical practice including an assessment for underlying causes. Pulmonary embolism should be considered as possible cause of new onset dyspnea given the risk of thrombosis associated with inhibition of VEGF signaling. Furthermore, fistula formation and pneumonia have been reported in subjects treated with cabozantinib and should be considered as clinically indicated in subjects presenting with pulmonary symptoms.

Electrolyte Abnormalities

Electrolyte abnormalities, including hypocalcemia, hypokalemia, hypomagnesemia, and hypophosphatemia have been noted in subjects treated with cabozantinib. In some cases, these have been Grade 3 or 4 and/or serious. These laboratory values should be evaluated routinely. Deficits should be corrected when an electrolyte abnormality is noted in order to avoid worsening. Correction of electrolyte abnormalities should be accompanied by increased frequency of monitoring.

6.2.2 Management of AEs Associated with Nivolumab

The following should be taken into consideration in decisions regarding dose interruptions:

- The assigned dose for the Nivolumab nivolumab is 480 mg IV every 4 weeks.
- Dose interruptions are allowed but dose reductions are not allowed.
- Dose modification criteria for irAEs and for guidance on reinstituting nivolumab are described below.
- If corticosteroids are initiated for treatment of irAEs, they must be tapered over ≥ 1 month to ≤ 10 mg/day oral prednisone or equivalent before nivolumab can be resumed.
- Guidelines for the management of irAEs of nivolumab (i.e., pneumonitis, myocarditis) are provided in Sections 6.2.2.1, 6.2.3 (diarrhea/colitis), and 6.2.4 (hepatic events).

Delay treatment with nivolumab for the following reasons. Treatment may be resumed in subjects following recovery to Grade 0-1:

- Grade 2 myocarditis
- Grade 2 pneumonitis
- Grade 2 nephritis
- Hepatic events:
    - ALT/AST increases to > 3 – ≤ 5x ULN, or
    - Total bilirubin increases to > 1.5 – ≤ 3x ULN
- Grade 2 or 3 diarrhea or colitis
- Grade 2 or 3 myositis
- Symptomatic adrenal insufficiency, hypothyroidism, or hyperthyroidism; Grade 2 or 3 hypophysitis; or Grade 3 or 4 hyperglycemia

- Grade 2 ocular inflammatory toxicity
- Amylase/lipase > 1 – ≤ 5x ULN with symptoms or > 5x ULN without symptoms
- Grade 3 or 4 infection
- Grade 2 infusion-related reactions
- Grade 3 rash
- Other Grade 2 or 3 AEs associated with Nivolumab

Permanently discontinue nivolumab for the below reasons.

- Grade 3 or 4 myocarditis and/or Grade 2 myocarditis unresolved while withholding nivolumab
- Grade 4 myositis and/or recurrent Grade 3 myositis
- Grade 3 or 4 pneumonitis
- Grade 3 or 4 nephritis
- Hepatic events:
  - AST/ALT increases to > 5x ULN, or
  - Total bilirubin increases to > 3x ULN, or
  - AST/ALT increases to > 3 – ≤ 5x ULN or total bilirubin increases to > 1.5 – ≤ 3x ULN that do not resolve to Grade 1 or better within 12 weeks, or
  - Drug-related ALT or AST > 3x ULN in combination with total bilirubin > 2x ULN without other reasonable explanation, consistent with drug-induced liver injury (DILI)
- Grade 4 diarrhea or colitis
- Grade 4 hypophysitis and/or recurrent hypophysitis
- Myasthenic syndrome/myasthenia gravis, Guillain-Barré or meningoencephalitis (all grades)
- Grade 3 or 4 ocular inflammatory toxicity
- Amylase/lipase > 5x ULN with symptoms or the below events that do not resolve to grade 1 or better within 12 weeks:
  - Amylase/lipase >1 – ≤ 5x ULN with symptoms
  - Amylase/lipase > 5x ULN without symptoms
- Grade 3 or 4 infusion-related reactions
- Grade 4 rash
- Other Grade 4 or recurrent Grade 3 AEs associated with nivolumab

6.2.2.1 Guidelines for Management of Potential Adverse Events Associated Nivolumab

Diarrhea/Colitis

See section 6.2.3 for guidance regarding both cabozantinib and nivolumab.

Hepatic Events

See section 6.2.4 for guidance regarding both cabozantinib and nivolumab.

Immune-Related Pulmonary Events

Dyspnea, cough, fatigue, hypoxia, pneumonitis, and pulmonary infiltrates have been associated with the administration of nivolumab. Subjects should be assessed for pulmonary signs and symptoms throughout the study.

All pulmonary events should be thoroughly evaluated for other commonly reported etiologies such as pneumonia or other infections, lymphangitic carcinomatosis, PE, heart failure, chronic obstructive pulmonary disease, or pulmonary hypertension. Management guidelines for pulmonary events are provided below.

Management Guidelines for Immune-Related Pulmonary Events, Including Pneumonitis

Severity of Event	Management
Grade 1	• Continue Nivolumab and monitor closely<br>• Re-evaluate on serial imaging<br>• Consider subject referral to pulmonary specialist<br>• For recurrent pneumonitis, treat as a Grade 3 or 4 event
Grade 2	• Withhold Nivolumab<br>• Refer subject to pulmonary and infectious disease specialists and consider bronchoscopy or BAL<br>• Initiate treatment with 1–2 mg/kg/day oral prednisone or equivalent.<br>• Resume Nivolumab if event resolves to Grade 1 or better within 12 weeks[a,b]<br>• Permanently discontinue Nivolumab and contact the Principal Investigator if event does not resolve to Grade 1 or better within 12 weeks[a,b,c]<br>• For recurrent events, treat as a Grade 3 or 4 event
Grade 3 or 4	• Permanently discontinue Nivolumab and contact the Principal Investigator[c]<br>• Bronchoscopy or BAL is recommended.<br>• Initiate treatment with 1–2 mg/kg/day oral prednisone or equivalent.<br>• If event does not improve within 48 hours after initiating corticosteroids, consider adding an immunosuppressive agent<br>• If event resolves to Grade 1 or better, taper corticosteroids over ≥ 1 month

BAL, bronchoscopic alveolar lavage.

[a] If corticosteroids have been initiated, they must be tapered over ≥ 1 month to ≤ 10 mg/day oral prednisone or equivalent before Nivolumab can be resumed.

[b] Nivolumab may be withheld for a period of time beyond 12 weeks to allow for corticosteroids to be reduced to ≤ 10 mg/day oral prednisone or equivalent. The acceptable length of the extended period of time must be agreed upon by the investigator and the Principal Investigator.

[c] Resumption of Nivolumab may be considered in subjects who are deriving benefit and have fully recovered from the immune-related event. Subjects can be rechallenged with Nivolumab only after approval has been documented by both the Investigator (or an appropriate delegate) and the Principal Investigator.

Immune-related Endocrinopathies

Thyroid disorders, adrenal insufficiency, and hypophysitis have been associated with the administration of Nivolumab. Management guidelines for endocrine events are provided below.

Monitor for signs and symptoms of hypophysitis. Subjects with unexplained symptoms such as fatigue, myalgias, impotence, mental status changes, or constipation should be investigated for the presence of thyroid, pituitary, or adrenal endocrinopathies. The subject should be referred to an endocrinologist if an endocrinopathy is suspected. Thyroid-stimulating hormone (TSH) and free triiodothyronine (T3) and thyroxine (T4) levels should be measured to determine whether thyroid abnormalities are present. TSH,

prolactin, and a morning cortisol level will help to differentiate primary adrenal insufficiency from primary pituitary insufficiency.

Management Guidelines for Endocrine Events

Event	Management
Hypophysitis (pan-hypopituitarism) Grade 2-3	• Withhold Nivolumab for up to 12 weeks after event onset[a]<br>• Refer patient to endocrinologist.<br>• Perform brain MRI (pituitary protocol).<br>• Initiate treatment with 1-2 mg/kg/day IV methylprednisolone or equivalent and convert to 1-2 mg/kg/day oral prednisone or equivalent upon improvement.<br>• Initiate hormone replacement therapy if clinically indicated.<br>• If event resolves to Grade 1 or better, resume Nivolumab.[a]<br>• If event does not resolve to Grade 1 or better while withholding Nivolumab, permanently discontinue Nivolumab and contact the Principal Investigator.[b]<br>• For recurrent hypophysitis, treat as a Grade 4 event.
Hypophysitis (pan-hypopituitarism) Grade 4	• Permanently discontinue Nivolumab and contact the Principal Investigator.<br>• Refer patient to endocrinologist.<br>• Perform brain MRI (pituitary protocol).<br>• Initiate treatment with 1-2 mg/kg/day IV methylprednisolone or equivalent and convert to 1-2 mg/kg/day oral prednisone or equivalent upon improvement.[c]<br>• Initiate hormone replacement therapy if clinically indicated.
Asymptomatic hypothyroidism	• Continue Nivolumab<br>• Initiate treatment with thyroid replacement hormone<br>• Monitor TSH every 3 weeks with a close monitoring for any sign and symptom
Symptomatic hypothyroidism	• Withhold Nivolumab<br>• Initiate treatment with thyroid replacement hormone<br>• Monitor TSH weekly<br>• Consider subject referral to endocrinologist.<br>• Resume Nivolumab when symptoms are controlled and thyroid function is improving
Asymptomatic hyperthyroidism	**TSH ≥ 0.1 mU/L and < 0.5 mU/L**:<br>• Continue Nivolumab<br>• Monitor TSH every 4 weeks<br>**TSH < 0.1 mU/L**:<br>• Follow guidelines for symptomatic hyperthyroidism
Symptomatic hyperthyroidism	• Withhold Nivolumab

	• Initiate treatment with anti-thyroid drug such as methimazole or carbimazole as needed<br>• Consider subject referral to endocrinologist<br>• Resume Nivolumab when symptoms are controlled and thyroid function is improving<br>• Permanently discontinue Nivolumab and contact the Principal Investigator for life-threatening immune-related hyperthyroidism[b]
Symptomatic adrenal insufficiency Grade 2–4	• Withhold Nivolumab[c]<br>• Refer subject to endocrinologist<br>• Perform appropriate imaging<br>• Initiate treatment with 1–2 mg/kg/day IV methylprednisolone or equivalent and convert to 1–2 mg/kg/day oral prednisone or equivalent upon improvement.<br>• Resume Nivolumab if event resolves to Grade 1 or better and subject is stable on replacement therapy (if required) within 12 weeks[a,c]<br>• Permanently discontinue Nivolumab and contact the Principal Investigator if event does not resolve to Grade 1 or better or subject is not stable on replacement therapy within 12 weeks[a,b,c]
Hyperglycemia Grade 1 or 2	• Continue Nivolumab<br>• Initiate treatment with insulin if needed<br>• Monitor for glucose control
Hyperglycemia Grade 3 or 4	• Withhold Nivolumab.<br>• Initiate treatment with insulin.<br>• Monitor for glucose control.<br>• Resume Nivolumab when symptoms resolve and glucose levels are stable.

IV, intravenous; MRI, magnetic resonance imaging; TSH, thyroid-stimulating hormone.

[a] Nivolumab may be withheld for a period of time beyond 12 weeks to allow for corticosteroids to be reduced to ≤ 10 mg/day oral prednisone or equivalent. The acceptable length of the extended period of time must be agreed upon by the investigator and the Principal Investigator.

[b] Resumption of Nivolumab may be considered in subjects who are deriving benefit and have fully recovered from the immune-related event. Subjects can be rechallenged with Nivolumab only after approval has been documented by both the investigator (or an appropriate delegate) and the Principal Investigator.

[c] If corticosteroids have been initiated, they must be tapered over ≥ 1 month to ≤ 10 mg/day oral prednisone or equivalent before Nivolumab can be resumed.

Immune-Related Dermatologic Events

Treatment-emergent rash has been associated with Nivolumab. The majority of cases of rash were mild in severity and self-limited, with or without pruritus. A dermatologist should evaluate persistent and/or severe rash or pruritus. A biopsy should be considered unless contraindicated. Management guidelines for dermatologic events are provided in the table below.

Nivolumab Management Guidance of Immune-Related Dermatologic Events

Severity of Event	Management of Skin Disorder
Grade 1	• Continue Nivolumab.<br>• Consider treatment with topical corticosteroids and/or other symptomatic therapy (e.g., antihistamines).
Grade 2	• Continue Nivolumab.<br>• Consider subject referral to dermatologist.<br>• Initiate treatment with topical corticosteroids.<br>• Consider treatment with higher-potency topical corticosteroids if event does not improve
Grade 3	• Delay Nivolumab.<br>• Refer subject to dermatologist.<br>• Initiate treatment with 10 mg/day oral prednisone or equivalent, increasing dose to 1− 2 mg/kg/day if event does not improve within 48− 72 hours.<br>• Resume Nivolumab if event resolves to Grade 1 or better within 12 weeks.[a,c]<br>• Permanently discontinue Nivolumab and contact Principal Investigator if event does not resolve to Grade 1 or better within 12 weeks.[a,b,c]
Grade 4	• Permanently discontinue Nivolumab and contact Principal Investigator.

[a] If corticosteroids have been initiated, they must be tapered over ≥ 1 month to ≤ 10 mg/day oral prednisone or equivalent before Nivolumab can be resumed.

[b] Nivolumab may be withheld for a period of time beyond 12 weeks to allow for corticosteroids to be reduced to ≤ 10 mg/day oral prednisone or equivalent. The acceptable length of the extended period of time must be agreed upon by the investigator and the Principal Investigator.

[c] Resumption of Nivolumab may be considered in subjects who are deriving benefit and have fully recovered from the immune-related event. Subjects can be rechallenged with Nivolumab only after approval has been documented by both the investigator (or an appropriate delegate) and the Principal Investigator.

Immune-Related Ocular Events

Treatment-emergent ocular events have been associated with Nivolumab. Management guidelines for ocular events are provided in the table below.

Nivolumab Management Guidance of Immune-Related Ocular Events

Severity of Event	Management of Ocular Event
Grade 1	• Continue Nivolumab.<br>• Subject referral to ophthalmologist is strongly recommended.<br>• Initiate treatment with topical corticosteroid eye drops and topical immunosuppressive therapy.<br>• If symptoms persist, treat as a Grade 2 event.
Grade 2	• Delay Nivolumab.<br>• Subject referral to ophthalmologist is strongly recommended.<br>• Initiate treatment with topical corticosteroid eye drops and topical immunosuppressive therapy.<br>• Resume Nivolumab if event resolves to Grade 1 or better within 12 weeks.[a,b]<br>• Permanently discontinue Nivolumab and contact the Principal Investigator if event does not resolve to Grade 1 or better within 12 weeks.[a,b,c]
Grade 3 or 4	• Permanently discontinue Nivolumab and contact the Principal Investigator.[c]<br>• Refer subject to ophthalmologist.<br>• Initiate treatment with 1− 2 mg/kg/day oral prednisone or equivalent.<br>• If event resolves to Grade 1 or better, taper corticosteroids over ≥ 1 month.

[a] If corticosteroids have been initiated, they must be tapered over ≥ 1 month to ≤ 10 mg/day oral prednisone or equivalent before Nivolumab can be resumed.

[b] Nivolumab may be withheld for a period of time beyond 12 weeks to allow for corticosteroids to be reduced to ≤ 10 mg/day oral prednisone or equivalent. The acceptable length of the extended period of time must be agreed upon by the investigator and the Principal Investigator.

[c] Resumption of Nivolumab may be considered in subjects who are deriving benefit and have fully recovered from the immune-related event. Subjects can be rechallenged with Nivolumab only after approval has been documented by both the investigator (or designee) and the Principal Investigator.

Immune-Related Meningoencephalitis

Immune-related meningoencephalitis is an identified risk associated with the administration of Nivolumab. Immune-related meningoencephalitis should be suspected in any subject presenting with signs or symptoms suggestive of meningitis or encephalitis, including, but not limited to, headache, neck pain, confusion, seizure, motor or sensory dysfunction, and altered or depressed level of consciousness. Encephalopathy from metabolic or electrolyte imbalances needs to be distinguished from potential meningoencephalitis resulting from infection (bacterial, viral, or fungal) or progression of malignancy, or secondary to a paraneoplastic process.

All subjects being considered for meningoencephalitis should be urgently evaluated with a CT scan and/or MRI scan of the brain to evaluate for metastasis, inflammation, or edema. If deemed safe by the treating physician, a lumbar puncture should be performed and a neurologist should be consulted.

Subjects with signs and symptoms of meningoencephalitis, in the absence of an identified alternate etiology, should be treated according to the guidelines in the table below.

Management Guidelines for Immune-Related Meningoencephalitis

Severity of Event	Management
All grades	• Permanently discontinue Nivolumab and contact the Principal Investigator[a]<br>• Refer subject to neurologist<br>• Initiate treatment with 1–2 mg/kg/day IV methylprednisolone or equivalent and convert to 1–2 mg/kg/day oral prednisone or equivalent upon improvement<br>• If event does not improve within 48 hours after initiating corticosteroids, consider adding an immunosuppressive agent<br>• If event resolves to Grade 1 or better, taper corticosteroids over ≥ 1 month

IV, intravenous.

[a] Resumption of Nivolumab may be considered in subjects who are deriving benefit and have fully recovered from the immune-related event. Subjects can be rechallenged with Nivolumab only after approval has been documented by both the investigator (or an appropriate delegate) and the Principal Investigator.

Immune-Related Motor and Sensory Neuropathy

Myasthenia gravis and Guillain-Barré syndrome have been observed with single-agent Nivolumab. Patients may present with signs and symptoms of sensory and/or motor neuropathy. Diagnostic work-up is essential for an accurate characterization to differentiate between alternative etiologies. Management guidelines for neurologic disorders are provided in the table below.

Management Guidelines for Immune-Related Neurologic Disorders

Event	Management
Immune-related neuropathy Grade 1	• Continue Nivolumab<br>• Investigate etiology
Immune-related neuropathy Grade 2	• Withhold Nivolumab<br>• Investigate etiology<br>• Initiate treatment as per institutional guidelines<br>• Resume Nivolumab if event resolves to Grade 1 or better within 12 weeks[a,b]<br>• Permanently discontinue Nivolumab and contact the Principal Investigator if event does not resolve to Grade 1 or better within 12 weeks[a,b,c]
Immune-related neuropathy Grade 3 or 4	• Permanently discontinue Nivolumab and contact the Principal Investigator[c]<br>• Initiate treatment as per institutional guidelines.
Myasthenia gravis and Guillain-Barré syndrome, any grade	• Permanently discontinue Nivolumab and contact the Principal Investigator[c]<br>• Refer subject to neurologist.<br>• Initiate treatment as per institutional guidelines.<br>• Consider initiation of 1–2 mg/kg/day oral or IV prednisone or equivalent.

IV, intravenous.

[a] If corticosteroids have been initiated, they must be tapered over ≥ 1 month to ≤ 10 mg/day oral prednisone or equivalent before Nivolumab can be resumed.

[b] Nivolumab may be withheld for a period of time beyond 12 weeks to allow for corticosteroids to be reduced to ≤ 10 mg/day oral prednisone or equivalent. The acceptable length of the extended period of time must be agreed upon by the investigator and the Principal Investigator.

[c] Resumption of Nivolumab may be considered in subjects who are deriving benefit and have fully recovered from the immune-related event. Subjects can be rechallenged with Nivolumab only after approval has been documented by both the investigator (or an appropriate delegate) and the Principal Investigator.

Immune-Related Myocarditis

Non-fatal myocarditis has been associated with the administration of Nivolumab. Guidelines for management of immune-related myocarditis are presented in the table below.

Management Guidelines for Immune-Related Myocarditis

Event	Management
Immune-related myocarditis, Grade 1	• Refer patient to cardiologist<br>• Initiate treatment as per institutional guidelines.
Immune-related myocarditis, Grade 2	• Withhold Nivolumab for up to 12 weeks after event onset and contact Principal Investigator.<br>• Refer patient to cardiologist<br>• Initiate treatment as per institutional guidelines and consider antiarrhythmic drugs, temporary pacemaker, ECMO, or VAD as appropriate.<br>• Consider treatment with 1-2 mg/kg/day IV methylprednisolone or equivalent and convert to 1-2 mg/kg/day oral prednisone or equivalent upon improvement.[a]<br>• If event resolves to Grade 1 or better, resume Nivolumab.[b]<br>• If event does not resolve to Grade 1 or better while withholding Nivolumab, permanently discontinue Nivolumab and contact the Principal Investigator. [c]
Immune-related myocarditis, Grade 3-4	• Permanently discontinue Nivolumab and contact the Principal Investigator.[c]<br>• Refer patient to cardiologist<br>• Initiate treatment as per institutional guidelines and consider antiarrhythmic drugs, temporary pacemaker, ECMO, or VAD as appropriate.<br>• Initiate treatment with 1-2 mg/kg/day IV methylprednisolone or equivalent and convert to 1-2 mg/kg/day oral prednisone or equivalent upon improvement.[a,b]<br>• If event does not improve within 48 hours after initiating corticosteroids, consider adding an immunosuppressive agent.<br>• If event resolves to Grade 1 or better, taper corticosteroids over ≥ 1 month.

ECMO, extracorporeal membrane oxygenation; IV, intravenous; VAD, ventricular assist device.

[a] If corticosteroids have been initiated, they must be tapered over ≥ 1 month to ≤ 10 mg/day oral prednisone or equivalent before Nivolumab can be resumed.

[b] Nivolumab may be withheld for a period of time beyond 12 weeks to allow for corticosteroids to be reduced to ≤ 10 mg/day oral prednisone or equivalent. The acceptable length of the extended period of time must be agreed upon by the investigator and the Principal Investigator.

[c] Resumption of Nivolumab may be considered in subjects who are deriving benefit and have fully recovered from the immune-related event. Subjects can be rechallenged with Nivolumab only after approval has been documented by both the investigator (or an appropriate delegate) and the Principal Investigator.

Immune-Related Nephritis

Immune-related nephritis is a relatively rare complication of Nivolumab with the most common reported underlying pathology being acute tubulo-interstitial nephritis. The most common presentation is an asymptomatic increase in creatinine levels. In the absence of alternative etiologies (e.g., prerenal and postrenal causes, and concomitant medications), immune-related nephritis is defined as renal dysfunction requiring steroid treatment and/or confirmed by biopsy.

Nivolumab should be withheld for moderate (Grade 2) immune-related nephritis and permanently discontinued for severe nephritis (Grade 3 or 4). Refer subjects to a renal specialist and consider renal biopsy and supportive measures as indicated. Corticosteroids and/or additional immunosuppressive agents should be administered as clinically indicated. Refer to the current Nivolumab Investigator's Brochure or Prescribing Information for further guidance on the management of immune-related nephritis.

Guidelines for management of immune-related nephritis are presented in the table below.

Management Guidelines for Immune-Related Nephritis

Event	Management
Renal event, Grade 1	• Continue Nivolumab.<br>• Monitor kidney function, including creatinine, closely until values resolve to within normal limits or to baseline values.
Renal event, Grade 2	• Withhold Nivolumab for up to 12 weeks after event onset. [a]<br>• Refer patient to renal specialist.<br>• Initiate treatment with corticosteroids equivalent to 1−2 mg/kg/day oral prednisone.<br>• If event resolves to Grade 1 or better, resume Nivolumab. [b]<br>• If event does not resolve to Grade 1 or better while withholding Nivolumab, permanently discontinue Nivolumab and contact the Principal Investigator. [c]
Renal event, Grade 3 or 4	• Permanently discontinue Nivolumab and contact the Principal Investigator.<br>• Refer patient to renal specialist and consider renal biopsy.<br>• Initiate treatment with corticosteroids equivalent to 1−2 mg/kg/day oral prednisone.<br>• If event does not improve within 48 hours after initiating corticosteroids, consider adding an immunosuppressive agent.<br>• If event resolves to Grade 1 or better, taper corticosteroids over ≥ 1 month.

[a] Nivolumab may be withheld for a longer period of time (i.e., > 12 weeks after event onset) to allow for corticosteroids (if initiated) to be reduced to the equivalent of ≤ 10 mg/day oral prednisone. The acceptable length of the extended period of time must be agreed upon by the Investigator and the Principal Investigator.

[b] If corticosteroids have been initiated, they must be tapered over ≥ 1 month to the equivalent of ≤ 10 mg/day oral prednisone before Nivolumab can be resumed.

[c] Resumption of Nivolumab may be considered in patients who are deriving benefit and have fully recovered from the immune-related event. Subjects can be rechallenged with Nivolumab only after approval has been documented by both the Investigator (or an appropriate delegate) and the Principal Investigator.

Immune-Related Myositis

Immune-related myositis has been associated with the administration of Nivolumab. Dermatomyositis and polymyositis are amongst the most common disorders. Initial diagnosis is based on clinical (muscle weakness, muscle pain, skin rash in dermatomyositis), biochemical (serum creatinine-kinase increase), and imaging (electromyography/MRI) features and is confirmed with a muscle biopsy. Nivolumab should be withheld for moderate or severe (Grade 2 or 3) immune-related myositis and permanently discontinued for recurrent severe or life-threatening myositis (recurrent Grade 3 or Grade 4). Please refer the subject to rheumatologist and/or neurologist and consider muscle biopsy and supportive measures as clinically indicated. Corticosteroids treatment with 1-2 mg/kg/day IV methylprednisolone or higher-dose bolus if severely compromised (weakness severely limiting mobility, cardiac function, respiratory function, dysphagia) and/or additional immunosuppressive agents should be administered for ≥ Grade 2 events or if the event does not improve after initial corticosteroids.

Detailed guidelines for management of immune-related myositis are presented in the table below.

Management Guidelines for Immune-Related Myositis

Event	Management
Immune-related myositis, Grade 1	• Continue Nivolumab<br>• Refer subject to rheumatologist or neurologist<br>• Initiate treatment as per institutional guidelines
Immune-related myositis, Grade 2	• Withhold Nivolumab for up to 12 weeks after event onset[a] and contact Principal Investigator.<br>• Refer subject to rheumatologist or neurologist<br>• Initiate treatment as per institutional guidelines<br>• Consider treatment with corticosteroid equivalent to 1-2 mg/kg/day IV methylprednisolone and convert to 1-2 mg/kg/day oral prednisone or equivalent upon improvement.<br>• If corticosteroids are initiated and event does not improve within 48 hours after initiating corticosteroids, consider adding an immunosuppressive agent<br>• If event resolves to Grade 1 or better, resume Nivolumab.[b]<br>• If event does not resolve to Grade 1 or better while withholding Nivolumab,

Immune-related myositis, Grade 3	• Withhold Nivolumab for up to 12 weeks after event onset[a] and contact the Principal Investigator<br>• Refer subject to rheumatologist or neurologist<br>• Initiate treatment as per institutional guidelines<br>• Respiratory support may be required in more severe cases<br>• Initiate treatment with corticosteroid equivalent to 1-2 mg/kg/day IV methylprednisolone or higher dose bolus if subject is severely compromised (e.g., cardiac or respiratory symptoms, dysphagia, or weakness that severely limits mobility); convert to 1-2 mg/kg/day oral prednisone or equivalent upon improvement.<br>• If event does not improve within 48 hours after initiating corticosteroids, consider adding an immunosuppressive agent.<br>• If event resolves to Grade 1 or better, resume Nivolumab.[b]<br>• If event does not resolve to Grade 1 or better while withholding Nivolumab, permanently discontinue Nivolumab and contact the Principal Investigator. [c]<br>• For recurrent events, treat as a Grade 4 event
Immune-related myositis, Grade 4	• Permanently discontinue Nivolumab and contact the Principal Investigator.[c]<br>• Refer subject to rheumatologist or neurologist<br>• Initiate treatment as per institutional guidelines. Respiratory support may be required in more severe cases<br>• Initiate treatment with corticosteroid equivalent to 1-2 mg/kg/day IV methylprednisolone or higher dose bolus if subject is severely compromised (e.g., cardiac or respiratory symptoms, dysphagia, or weakness that severely limits mobility); convert to 1-2 mg/kg/day oral prednisone or equivalent upon improvement.<br>• If event does not improve within 48 hours after initiating corticosteroids, consider adding an immunosuppressive agent.<br>• If event resolves to Grade 1 or better, taper corticosteroids over ≥ 1 month.

IV, intravenous

[a] Nivolumab may be withheld for a period of time (i.e., > 12 weeks after event onset) to allow for corticosteroids (if initiated) to be reduced to ≤ 10 mg/day oral prednisone or equivalent. The acceptable length of the extended period of time must be agreed upon by the investigator and the Principal Investigator.

[b] If corticosteroids have been initiated, they must be tapered over ≥ 1 month to ≤ 10 mg/day oral prednisone or equivalent before Nivolumab can be resumed.

[c] Resumption of Nivolumab may be considered in subjects who are deriving benefit and have fully recovered from the immune-related event. Subjects can be rechallenged with Nivolumab only after approval has been documented by both the investigator (or an appropriate delegate) and the Principal Investigator.

Immune-Related Pancreatitis

Symptoms of abdominal pain associated with elevations of amylase and lipase, suggestive of pancreatitis, have been associated with the administration of Nivolumab. The differential diagnosis of acute abdominal pain should include pancreatitis. Appropriate work-up should include an evaluation for ductal obstruction, as well as serum amylase and lipase tests.

Management of Pancreatic Events, including Pancreatitis, Associated with Nivolumab

<table>
<tr><th>Amylase/Lipase</th><th>WITHOUT Symptoms</th><th>WITH Symptoms</th></tr>
<tr><td>Amylase/lipase
> 1 – ≤ 1.5x ULN</td><td>• Continue Nivolumab
• Monitor amylase and lipase prior to dosing</td><td rowspan="3">• Withhold Nivolumab
• Refer subject to GI specialist
• Initiate treatment with 1–2 mg/kg/day IV methylprednisolone or equivalent and convert to 1–2 mg/kg/day oral prednisone or equivalent upon improvement
• Resume Nivolumab if event resolves to Grade 1 or better within 12 weeks[a,b]
• Permanently discontinue Nivolumab and contact the Principal Investigator if event does not resolve to Grade 1 or better within 12 weeks[a,b,c]
• For recurrent events, permanently discontinue Nivolumab and contact the Principal Investigator[c]</td></tr>
<tr><td>Amylase/lipase
>1.5 – ≤ 2x ULN</td><td rowspan="2">• Continue Nivolumab
• Monitor amylase and lipase weekly
• For prolonged elevation (e.g., > 3 weeks), consider treatment with 10 mg/day oral prednisone or equivalent</td></tr>
<tr><td>Amylase/lipase
> 2 – ≤ 5x ULN</td></tr>
<tr><td>Amylase/lipase
> 5x ULN</td><td>• Withhold Nivolumab
• Refer subject to GI specialist
• Monitor amylase and lipase every other day
• If no improvement, consider treatment with 1–2 mg/kg/day oral prednisone or equivalent
• Resume Nivolumab if event resolves to Grade 1 or better within 12 weeks[a,b]
• Permanently discontinue Nivolumab and contact the Principal Investigator if event does not resolve to Grade 1 or better within 12 weeks[a,b,c]
• For recurrent events, permanently discontinue Nivolumab and contact the Principal Investigator[c]</td><td>• Permanently discontinue Nivolumab and contact the Principal Investigator[c]
• Refer subject to GI specialist.
• Initiate treatment with 1–2 mg/kg/day IV methylprednisolone or equivalent and convert to 1–2 mg/kg/day oral prednisone or equivalent upon improvement.
• If event does not improve within 48 hours after initiating corticosteroids, consider adding an immunosuppressive agent.
• If event resolves to Grade 1 or better, taper corticosteroids over ≥ 1 month.</td></tr>
</table>

GI, gastrointestinal; IV, intravenous.
[a] If corticosteroids have been initiated, they must be tapered over ≥ 1 month to ≤ 10 mg/day oral prednisone or equivalent before Nivolumab can be resumed.
[b] Nivolumab may be withheld for a period of time beyond 12 weeks to allow for corticosteroids to be reduced to ≤ 10 mg/day oral prednisone or equivalent. The acceptable length of the extended period of time must be agreed upon by the investigator and the Principal Investigator.
[c] Resumption of Nivolumab may be considered in subjects who are deriving benefit and have fully recovered from the immune-related event. Subjects can be rechallenged with Nivolumab only after approval has been documented by both the investigator (or an appropriate delegate) and the Principal Investigator.

Infusion-Related Reactions

Infusion-related reactions (IRRs) are known to occur with the administration of monoclonal antibodies and have been reported with IO therapies. These reactions, which are thought to be due to release of cytokines and/or other chemical mediators, occur within 24 h of administration and are generally mild to moderate in severity.

Infusion reactions may manifest with fever, chills, rigors, headache, rash, pruritus, arthralgias, hypotension, hypertension, bronchospasm, back pain, or other allergic-like reactions. Regardless of whether the event is attributed to these study drugs, all Grade 3 or 4 infusion reactions should be reported within 24 hours to the Principal Investigator and reported as an SAE if it meets the criteria.

Administer premedication if indicated for Cycle 1 of Nivolumab. Subjects who experience an IRR with Nivolumab may receive premedication with antihistamines, antipyretics, and/or analgesics (e.g., acetaminophen) for subsequent infusions.

High-level treatment recommendations are provided below and may be modified based on local treatment standards and guidelines, as appropriate:

Management of Infusion Reactions

Event	Management
Grade 1	• Consider reducing the infusion rate.<br>• Monitor vital signs as clinically indicated.<br>• Consider premedication prior to the next infusion.
Grade 2	• Interrupt infusion.<br>• Provide supportive therapy.<br>• Monitor vital signs as clinically indicated.<br>• Restart infusion at a lower rate if symptoms resolve.<br>• Premedicate prior to the next infusion.
Grade 3- 4	• Interrupt infusion and permanently discontinue Nivolumab.<br>• Provide supportive therapy.<br>• Monitor vital signs as clinically indicated.<br>• Hospitalize as indicated for clinical sequelae.

Cytokine Release Syndrome (CRS)

Cytokine-release syndrome (CRS) is defined as a supraphysiologic response following administration of any immune therapy that results in activation or engagement of endogenous or infused T cells and/or other immune effector cells. Symptoms can be progressive, always include fever at the onset, and may include hypotension, capillary leak (hypoxia), and end-organ dysfunction.[19] CRS has been well documented with chimeric antigen receptor T cell therapies and bispecific T-cell engager antibody therapies but has also been reported with immunotherapies that target PD-1 or PD-L1.[20,21]

Administer premedication if indicated for Cycle 1 of Nivolumab. Subjects who experience a CRS with Nivolumab may receive premedication with antihistamines, antipyretics, and/or analgesics (e.g., acetaminophen) for subsequent infusions.

Event	Management
**Grade 1**[a]<br>Fever[b] with or without constitutional symptoms	• Immediately interrupt infusion.<br>• Upon symptom resolution, wait for 30 minutes and then restart infusion at half the rate being given at the time of event onset.<br>• If the infusion is tolerated at the reduced rate for 30 minutes, the infusion rate may be increased to the original rate.<br>• If symptoms recur, discontinue infusion of this dose.<br>• Administer symptomatic treatment[c], including maintenance of IV fluids for hydration.<br>• In case of rapid decline or prolonged CRS (> 2 days) or in subjects with significant symptoms and/or comorbidities, consider managing as per Grade 2.<br>• For subsequent infusions, consider administration of oral premedication with antihistamines, antipyretics, and/or analgesics, and monitor closely for IRRs and/or CRS.
**Grade 2**[a]<br>Fever[b] with hypotension not requiring vasopressors<br>and/or<br>Hypoxia requiring low-flow oxygen[d] by nasal cannula or blow-by	• Immediately interrupt infusion.<br>• Upon symptom resolution, wait for 30 minutes and then restart infusion at half the rate being given at the time of event onset.<br>• If symptoms recur, discontinue infusion of this dose.<br>• Administer symptomatic treatment.[c]<br>• For hypotension, administer IV fluid bolus as needed.<br>• Monitor cardiopulmonary and other organ function closely (in the ICU, if appropriate). Administer IV fluids as clinically indicated, and manage constitutional symptoms and organ toxicities as per institutional practice.<br>• Rule out other inflammatory conditions that can mimic CRS (e.g., sepsis). If no improvement within 24 hours, initiate workup and assess for signs and symptoms of HLH or MAS.<br>• Consider IV corticosteroids (suggest: methylprednisolone 2 mg/kg/day or dexamethasone 10 mg every 6 hours).<br>• Consider anti-cytokine therapy.[e]<br>• Consider hospitalization until complete resolution of symptoms. If no improvement within 24 hours, manage as per Grade 3, i.e., hospitalize subject (monitoring in the ICU is recommended), permanently discontinue Nivolumab, and contact the Principal Investigator.<br>• If symptoms resolve to Grade 1 or better for 3 consecutive days, the next dose of Nivolumab may be administered. For subsequent infusions, consider administration of oral premedication with antihistamines, antipyretics, and/or analgesics and monitor closely for IRRs and/or CRS.<br>• If symptoms do not resolve to Grade 1 or better for 3 consecutive days, contact the Principal Investigator.

**Grade 3**[a]<br>Fever[b] with hypotension requiring a vasopressor (with or without vasopressin) and/or<br>Hypoxia requiring high-flow oxygen[d] by nasal cannula, face mask, non-rebreather mask, or venturi mask	• Permanently discontinue Nivolumab and contact the Principal Investigator.<br>• Administer symptomatic treatment.[c]<br>• For hypotension, administer IV fluid bolus and vasopressor as needed.<br>• Monitor cardiopulmonary and other organ function closely; monitoring in the ICU is recommended. Administer IV fluids as clinically indicated, and manage constitutional symptoms and organ toxicities as per institutional practice.<br>• Rule out other inflammatory conditions that can mimic CRS (e.g., sepsis). If no improvement within 24 hours, initiate workup and assess for signs and symptoms of HLH or MAS.<br>• Administer IV corticosteroids (suggest: methylprednisolone 2 mg/kg/day or dexamethasone 10 mg every 6 hours).<br>• Consider anti-cytokine therapy.[e]<br>• Hospitalize subject until complete resolution of symptoms. If no improvement within 24 hours, manage as per Grade 4, i.e., admit subject to ICU and initiate hemodynamic monitoring, mechanical ventilation, and/or IV fluids and vasopressors as needed; for subjects who are refractory to anti-cytokine therapy, experimental treatments may be considered at the discretion of the investigator and in consultation with the Principal Investigator.
**Grade 4**[a]<br>Fever[b] with hypotension requiring multiple vasopressors (excluding vasopressin) and/or<br>Hypoxia requiring oxygen by positive pressure (e.g., CPAP, BiPAP, intubation and mechanical ventilation)	• Permanently discontinue Nivolumab and contact the Principal Investigator.<br>• Administer symptomatic treatment.[c]<br>• Admit subject to ICU and initiate hemodynamic monitoring, mechanical ventilation, and/or IV fluids and vasopressors as needed. Monitor other organ function closely. Manage constitutional symptoms and organ toxicities as per institutional practice.<br>• Rule out other inflammatory conditions that can mimic CRS (e.g., sepsis). If no improvement within 24 hours, initiate workup and assess for signs and symptoms of HLH or MAS.<br>• Administer IV corticosteroids (suggest: methylprednisolone 2 mg/kg/day or dexamethasone 10 mg every 6 hours).<br>• Consider anti-cytokine therapy[e]. For subjects who are refractory to anticytokine therapy, experimental treatments[f] may be considered at the discretion of the investigator and in consultation with the Principal Investigator.<br>• Hospitalize subject until complete resolution of symptoms.

ASTCT, American Society for Transplantation and Cellular Therapy; BiPAP, bi-level positive airway pressure; CAR, chimeric antigen receptor; CPAP, continuous positive airway pressure; CRS, cytokine-release syndrome; CTCAE, Common Terminology Criteria for Adverse Events; eCRF, electronic Case Report Form; HLH, hemophagocytic lymphohistiocytosis; ICU, intensive care unit; IRR, infusion-related reaction; MAS, macrophage activation syndrome; NCCN, National Cancer Comprehensive Network; NCI, National Cancer Institute.

Note: The management guidelines have been adapted from NCCN guidelines for management of CAR T-cell-related toxicities (Version 2.2019).

a Grading system for management guidelines is based on ASTCT consensus grading for CRS. NCI CTCAE (version as specified in the protocol) should be used when reporting severity of IRRs, CRS, or organ toxicities associated with CRS on the Adverse Event eCRF. Organ toxicities associated with CRS should not influence overall CRS grading.

b Fever is defined as temperature > 38°C not attributable to any other cause. In subjects who develop CRS and then receive antipyretic, anti-cytokine, or corticosteroid therapy, fever is no longer required when subsequently determining event severity (grade). In this case, the grade is driven by the presence of hypotension and/or hypoxia.

c Symptomatic treatment may include oral or IV antihistamines, antipyretics, analgesics, bronchodilators, and/or oxygen. For bronchospasm, urticaria, or dyspnea, additional treatment may be administered as per institutional practice.

[d] Low flow is defined as oxygen delivered at ≤ 6 L/min, and high flow is defined as oxygen delivered at > 6 L/min.
[e] There are case reports where anti-cytokine therapy has been used for treatment of CRS with immune checkpoint inhibitors (Rotz et al 2017; Adashek and Feldman 2019), but data are limited, and the role of such treatment in the setting of antibody-associated CRS has not been established.
[f] Refer to Riegler et al (2019) for information on experimental treatments for CRS.[22]

Hemophagocytic Lymphohistiocytosis and Macrophage Activation Syndrome

Immune-mediated reactions may involve any organ system and may lead to hemophagocytic lymphohistiocytosis (HLH) and macrophage activation syndrome (MAS).

Subjects with suspected HLH should be diagnosed according to published criteria by McClain and Eckstein (2014). A subject should be classified as having HLH if five of the following eight criteria are met:

- Fever ≥ 38.5°C
- Splenomegaly
- Peripheral blood cytopenia consisting of at least two of the following:
    - Hemoglobin < 90 g/L (9 g/dL) (< 100 g/L [10 g/dL] for infants < 4 weeks old)
    - Platelet count < 100 × 10^9/L (100,000/μL)
    - ANC < 1.0 × 10^9/L (1000/μL)
- Fasting triglycerides > 2.992 mmol/L (265 mg/dL) and/or fibrinogen < 1.5 g/L (150 mg/dL)
- Hemophagocytosis in bone marrow, spleen, lymph node, or liver
- Low or absent natural killer cell activity
- Ferritin > 500 mg/L (500 ng/mL)
- Soluble interleukin 2 (IL-2) receptor (soluble CD25) elevated ≥ 2 standard deviations above age-adjusted laboratory-specific norms

Subjects with suspected MAS should be diagnosed according to published criteria for systemic juvenile idiopathic arthritis by Ravelli et al (2016). A febrile patient should be classified as having MAS if the following criteria are met:

- Ferritin > 684 mg/L (684 ng/mL)
- At least two of the following:
    - Platelet count ≤ 181 × 10^9/L (181,000/μL)

- AST ≥ 48 U/L
- Triglycerides > 1.761 mmol/L (156 mg/dL)
- Fibrinogen ≤ 3.6 g/L (360 mg/dL)

Patients with suspected HLH or MAS should be treated according to the guidelines the table below.

Management Guidelines for Suspected HLH or MAS

Event	Management
Suspected HLH or MAS	• Permanently discontinue Nivolumab and contact Principal Investigator.<br>• Consider patient referral to hematologist.<br>• Initiate supportive care, including intensive care monitoring if indicated per institutional guidelines.<br>• Consider initiation of IV corticosteroids and/or an immunosuppressive agent.<br>• If event does not improve within 48 hours after initiating corticosteroids, consider adding an immunosuppressive agent.<br>• If event resolves to Grade 1 or better, taper corticosteroids over ≥ 1 month.
HLH, hemophagocytic lymphohistiocytosis; MAS, macrophage activation syndrome	

Other Immune-Related Adverse Events

For management of other irAEs not included in this protocol the following general management guidance should be applied:

- Grade 2 or 3: delay Nivolumab dosing up to 12 weeks until irAE recovers to Grade 0-1 and corticosteroids have been reduced to ≤ 10 mg prednisone or equivalent per day

- Grade 4 or recurrent Grade 3: permanently discontinue Nivolumab

6.2.3 Management of Diarrhea/Colitis – Cabozantinib and Nivolumab

Cabozantinib

Subjects should be instructed to notify their physician immediately at the first signs of poorly formed or loose stools or an increased frequency of bowel movements. Guidelines for the evaluation and management of diarrhea are shown below.

Administration of antidiarrheal/antimotility agents is recommended at the first sign of diarrhea as initial management. Some subjects may require concomitant treatment with more than one antidiarrheal agent. When therapy with antidiarrheal agents does not control the diarrhea to tolerable levels, cabozantinib should be temporarily interrupted or dose reduced. When the diarrhea is controlled, retreatment with cabozantinib may be acceptable per Investigator decision.

In addition, general supportive measures should be implemented such as continuous oral isotonic hydration, correction of fluid and electrolyte abnormalities, small frequent meals, and stopping lactose-containing products, high-fat meals, and alcohol.

Recurrent or prolonged diarrhea can be associated with anal or perianal skin erosions which increase the risk for anal abscesses, fistulas, or proctitis. Good personal hygiene should be emphasized. Regular examinations of the perianal region should be performed whenever diarrhea has occurred during treatment with cabozantinib. Infections of the perianal region should be treated per local guidelines.

Management of Diarrhea Associated with Cabozantinib

Event	Management
Tolerable Grade 1-2<br>(duration < 48 h)	• Continue with study treatment and consider dose reduction.<br>• Initiate treatment with an antidiarrheal agent (e.g., loperamide 4 mg followed by 2 mg after each episode of diarrhea [maximum: 16 mg loperamide per day]).<br>• Dietary modifications (e.g., small lactose-free meals, bananas and rice).<br>• Intake of isotonic fluids (1-1.5 L/day).<br>• Re-assess after 24 hours:<br>o Diarrhea resolving to baseline bowel habits: gradually add solid foods and discontinue or decrease antidiarrheal treatment after 12 h diarrhea-free interval.<br>o Diarrhea not resolving: Continue/resume antidiarrheal treatment.
Intolerable Grade 2,<br>Grade 2 > 48 h,<br>or ≥ Grade 3	• Interrupt study treatment.<br>• Ask subject to attend clinic.<br>• Rule out infection (e.g., stool sample for culture).<br>o Administer antibiotics as needed (e.g., if fever or Grade 3-4 neutropenia persists > 24 h).<br>• Administer fluids (1-1.5 L/day orally or IV, as appropriate) for hydration or to correct electrolyte abnormalities.<br>• For Grade 3-4 or complicated lower grade diarrhea consider hospitalization and IV hydration.<br>• Re-assess after 24 h.<br>o Diarrhea resolving to baseline bowel habits or Grade ≤ 1: consider restarting study treatment at reduced dose.<br>o Diarrhea not resolving: Start and or continue antidiarrheal treatment (e.g., loperamide 4 mg followed by 2 mg after each episode of diarrhea [maximum: 16 mg loperamide per day]). Consider starting second line antidiarrheal or referral to gastroenterologist.

Nivolumab

Immune-related colitis has been associated with Nivolumab. Management guidelines for diarrhea or colitis are provided below. All events of diarrhea or colitis should be thoroughly evaluated for other more common etiologies. Note, opiate and narcotic medications may mask symptoms of perforation.

Management of Diarrhea or Colitis Associated with Nivolumab

Severity of Event	Management
Grade 1	• Continue Nivolumab<br>• Initiate symptomatic treatment<br>• Endoscopy is recommended if symptoms persist for > 7 days<br>• Monitor closely
Grade 2	• Withhold Nivolumab<br>• Initiate symptomatic treatment<br>• Subject referral to GI specialist is recommended<br>• For recurrent events or events that persist > 5 days, initiate treatment with 1–2 mg/kg/day oral prednisone or equivalent<br>• Resume Nivolumab if event resolves to Grade 1 or better within 12 weeks[a,b]<br>• Permanently discontinue Nivolumab and contact the Principal Investigator if event does not resolve to Grade 1 or better within 12 weeks[a,b,c]
Grade 3	• Withhold Nivolumab<br>• Refer subject to GI specialist for evaluation and confirmatory biopsy<br>• Initiate treatment with 1–2 mg/kg/day IV methylprednisolone or equivalent and convert to 1–2 mg/kg/day oral prednisone or equivalent upon improvement<br>• Resume Nivolumab if event resolves to Grade 1 or better within 12 weeks[a,b]<br>• Permanently discontinue Nivolumab and contact the Principal Investigator if event does not resolve to Grade 1 or better within 12 weeks[a,b,c]
Grade 4	• Permanently discontinue Nivolumab and contact the Principal Investigator[c]<br>• Refer subject to GI specialist for evaluation and confirmation biopsy.<br>• Initiate treatment with 1–2 mg/kg/day IV methylprednisolone or equivalent and convert to 1–2 mg/kg/day oral prednisone or equivalent upon improvement.<br>• If event does not improve within 48 hours after initiating corticosteroids, consider adding an immunosuppressive agent.<br>• If event resolves to Grade 1 or better, taper corticosteroids over ≥ 1 month.

GI, gastrointestinal; IV, intravenous

[a] If corticosteroids have been initiated, they must be tapered over ≥ 1 month to ≤ 10 mg/day oral prednisone or equivalent before Nivolumab can be resumed.

[b] Nivolumab may be withheld for a period of time beyond 12 weeks to allow for corticosteroids to be reduced to ≤ 10 mg/day oral prednisone or equivalent. The acceptable length of the extended period of time must be agreed upon by the investigator and the Principal Investigator.

[c] Resumption of Nivolumab may be considered in subjects who are deriving benefit and have fully recovered from the immune-related event. Subjects can be rechallenged with Nivolumab only after approval has been documented by both the Investigator (or an appropriate delegate) and the Principal Investigator.

6.2.4 Management of Hepatic Events – Cabozantinib and Nivolumab

Elevations of aminotransferases (ALT and AST) and bilirubin have been observed during treatment with cabozantinib and Nivolumab. It is recommended that subjects with elevation of ALT, AST, or bilirubin have more frequent laboratory monitoring of these parameters. If possible, hepatotoxic concomitant medications should be discontinued in subjects who develop increased values of ALT, AST, or bilirubin, and other causes (e.g., cancer related, infection) should be evaluated. Subjects with right upper-quadrant abdominal pain and/or unexplained nausea or vomiting should have liver function tests (LFTs) performed immediately and reviewed before administration of the next dose of study drug.

For subjects with elevated LFTs, concurrent medication, viral hepatitis, and toxic or neoplastic etiologies should be considered and addressed, as appropriate.

Management of Hepatic Events Associated with Cabozantinib and Nivolumab

<table>
<tr><th>Event</th><th>Cabozantinib</th><th>Nivolumab</th></tr>
<tr><td>ALT or AST
> 1 – ≤ 3x ULN[a,b]
OR
Total bilirubin
> 1 – ≤ 1.5x ULN[a]</td><td>• Dose adjustment is usually not required.
• Consider discontinuing concomitant hepatotoxic medications and adding supportive care as indicated.</td><td>• Continue Nivolumab.
• Monitor LFTs until values resolve to within normal limits.</td></tr>
<tr><td>ALT or AST
> 3 – ≤ 5x ULN[a,b]
OR
Total bilirubin
> 1.5 – ≤ 3x ULN[a]</td><td>• Delay cabozantinib if lasting longer than 1 week.
• Restart cabozantinib after lab abnormalities have resolved to CTCAE Grade ≤ 1 or baseline grade at the same dose level prior to dose interruption or one dose level lower at the discretion of the Principal Investigator.</td><td>• Monitor LFTs more frequently until return to baseline values.
• Delay Nivolumab.
• Initiate treatment with 1-2 mg/kg/day oral prednisone or equivalent
• Resume Nivolumab if event resolves to Grade 1 or better within 12 weeks[c,d]
• Discontinue Nivolumab if event does not resolve to Grade 1 or better within 12 weeks[c,d]</td></tr>
<tr><td>ALT or AST
> 5 – ≤ 8x ULN[a,b]
OR
Total bilirubin
> 3 – ≤ 10x ULN[a]</td><td>• Delay cabozantinib and consider more frequent monitoring of ALT, AST, and bilirubin.
• Restart cabozantinib at a reduced dose after lab abnormalities have resolved to CTCAE Grade ≤ 1 or baseline grade.
• Discontinue if lab abnormalities cannot be reversed despite interruption of cabozantinib.</td><td rowspan="2">• Discontinue Nivolumab.
• Consider subject referral to GI specialist for evaluation and liver biopsy to establish etiology of hepatic injury.
• Initiate treatment with 1-2 mg/kg/day oral prednisone or equivalent.
• If event does not improve within 48 hours after initiating corticosteroids, consider adding an immunosuppressive agent.
• If event resolves to Grade 1 or better, taper corticosteroids over ≥ 1 month</td></tr>
<tr><td>ALT or AST
> 8x ULN[a]
OR
Total Bilirubin
> 10x ULN[a]</td><td>• Discontinue cabozantinib unless these laboratory abnormalities have recovered to Grade 1 or baseline level after an interruption and the Principal Investigator has approved reinstitution of cabozantinib at a reduced dose</td></tr>
<tr><td colspan="3">[a]DILI: The following condition requires PERMANENT discontinuation of both cabozantinib and Nivolumab: Drug-related ALT or AST >3x ULN in combination with total bilirubin >2x ULN without other reasonable explanation, consistent with drug-induced liver injury (DILI).
[b]Elevations of aminotransferases when hepatic metastases are present may not require dose modifications of cabozantinib if there are no progressive changes in the aminotransferases (less than a doubling) and if there are no progressive elevations in serum bilirubin concentration or coagulation factors.
[c]Nivolumab may be withheld for a longer period of time (i.e., > 12 weeks after event onset) to allow for corticosteroids (if initiated) to be reduced to ≤ 10 mg/day oral prednisone or equivalent. The acceptable length of the extended period of time must be agreed upon by the Principal Investigator.
[d]If corticosteroids have been initiated, they must be tapered over ≥ 1 month to ≤ 10 mg/day oral prednisone or equivalent before Nivolumab can be resumed.</td></tr>
</table>

7.0 ADVERSE EVENTS AND UNANTICIPATED PROBLEMS

7.1 Definitions

7.1.1 Adverse Event (AE)

An AE is any untoward medical occurrence in a patient or clinical trial subject who has been enrolled in a clinical study and who may have been administered an investigational product, regardless of whether or not the event is assessed as related to the study treatment. An adverse event can therefore be any unfavorable and unintended sign (including an abnormal laboratory finding), symptom, or disease temporarily associated with the use of an investigational product, regardless of whether or not the event is assessed as related to the investigational product. This requirement includes specific events or symptoms associated with cancer progression or general clinical deterioration to ensure potential toxicities are not overlooked. Radiographic progression without associated clinical sequalae is not considered an AE: terms such as ‘disease progression’ should be avoided. An adverse event can arise from any use of the drug (e.g. off-label use, use in combination with another drug) and with any route of administration, formulation, or dose, including an overdose. This definition also includes AEs associated with medication errors and uses of the investigational product outside what is in the protocol, including misuse and abuse. Pre-existing medical conditions that worsen during the study should be recorded as AEs. Abnormal laboratory values, ECG findings, or vital signs are to be recorded as AEs if they meet the criteria described in this protocol.

All untoward events that occur after informed consent through 30 days (100 days for SAEs and certain other events [See AEs of Special Interest Table]) after the date of the decision to permanently discontinue study treatment are to be recorded by the investigational site.

At each scheduled and unscheduled visit, AEs are to be identified and assessed based upon study procedures, routine and symptom-directed clinical investigations, and subject query/report. Seriousness, severity grade, and relationship to study treatment will be assessed by the Investigator. Severity grade will be defined by the current version of the National Cancer Institute (NCI)-Common Terminology Criteria for Adverse Events (CTCAE).

Assessment of the relationship of the AEs to individual study treatment by the Investigator is based on the following two definitions:

- Not Related: An event is assessed as not related to study drug if it is attributable to another cause and/or there is no evidence to support a causal relationship to the study treatment.
- Related: A related AE is defined as an AE where a causal relationship between the event and the study treatment is a reasonable possibility. A reasonable causal relationship is meant to convey that there are facts (e.g., evidence such as dechallenge/rechallenge) or other clinical arguments to suggest a causal relationship between the AE and study treatment. Possibly and probably related AEs should be documented as related.

7.1.2 Serious Adverse Event (SAE)

The SAE definition and reporting requirements are in accordance with the International Conference of Harmonisation (ICH) Guideline for Clinical Safety Data Management: Definitions and Standards for Expedited Reporting, Topic E2A.

An SAE is defined as any untoward medical occurrence that at any dose:

- Result in death.
- Is immediately life-threatening (i.e., in the opinion of the Investigator, the AE places the subject at immediate risk of death; it does not include an event that, had it occurred in a more severe form, might have caused death).
- Requires inpatient hospitalization or results in prolongation of an existing hospitalization.
    - Note: While most hospitalizations necessitate reporting of an SAE, some hospitalizations do not require SAE reporting, as follows: elective or previously scheduled surgeries or procedures for pre-existing conditions that have not worsened after initiation of treatment (e.g., a previously scheduled ventral hernia repair); pre-specified study hospitalizations for observation; or events that result in hospital stays of fewer than 24 hours and that do not require admission (e.g., an ER visit for hematuria that results in a diagnosis of cystitis and discharge home on oral antibiotics). SAEs must, however, be reported for any surgical complication resulting in prolongation of the hospitalization.
- Results in significant incapacity or substantial disruption of the ability to conduct normal life functions.
- Is a congenital anomaly or birth defect.
- Is an important medical event (IME).

- Note: The term “important medical event” refers to an event that, based upon appropriate medical judgment, may not be immediately life-threatening or result in death or hospitalization, but may jeopardize the subject or require medical or surgical intervention to prevent one of the outcomes listed above. Examples of IMEs include intensive treatment in an emergency room or at home for allergic bronchospasm; blood dyscrasias, or convulsions that do not result in hospitalization; or development of product dependency or product abuse.

7.1.3 Reporting SAEs to Exelixis

As soon as an Investigator becomes aware of an AE that meets the criteria for an SAE, the Investigator will document the SAE on an SAE Report Form or in an electronic database. SAEs, regardless of causal relationship, must be reported to Exelixis within one (1) business day of the Investigator’s knowledge of the event by submitting a completed SAE report form. The reports must be emailed to **drugsafety@exelixis.com** or faxed to **650-837-7392** with the below:

- Required Information:
  - Identity of Investigator
  - Site name
  - Subject identifiers
  - Drug name and dosage
  - Event description
  - Event terms (i.e., as recorded in the electronic database)
  - Investigator’s assessment of the relationship of the event to study treatment
  - The reason why the event is considered to be serious (i.e., the seriousness criteria)

- Recommended Information:
  - Medications or therapeutic measures used to treat the event
  - Action taken with the study treatment because of the event
  - Outcome/resolution of the event
  - Any additional SAE information

Note – Medical records should **not** be sent to Exelixis unless requested.

The Investigator will perform adequate due diligence with regard to obtaining follow-up information on incomplete reports. All follow-up information must be sent to Exelixis within one (1) business day of the Investigator's receipt of the new information.

SAEs that must be recorded on an SAE Reporting form include the following:

- SAEs that occur after informed consent or study initiation and through 100 days after the date of the decision to permanently discontinue study treatment
- SAEs assessed as related to study treatment or study procedures, even if the SAE occurs more than 100 days after the date of the decision to permanently discontinue study treatment.

In all cases, the Investigator should continue to monitor the clinical situation and report all material facts relating to the progression or outcome of the SAE. Furthermore, the Investigator may be required to provide supplementary information as requested by Exelixis.

When reporting SAEs, the following additional points will be noted:

- When the diagnosis of an SAE is known or suspected, the Investigator will report the diagnosis or syndrome as the primary SAE term, rather than as signs or symptoms. Signs and symptoms may then be described in the event description.
- Death will not be reported as an SAE, but as an outcome of a specific SAE, unless the event preceding the death is unknown. Terms of "Unexplained Death" or "Death from unknown origin" may be used when the cause is unknown. In these circumstances the cause of death must be investigated, and the diagnosis amended when the etiology has been identified. If an autopsy was performed, the autopsy report should be provided.

7.1.4 Regulatory Reporting

The Investigator will assess the expectedness of each related SAE to the study treatment. The current cabozantinib Reference Safety Information (Appendix K of the most recent approved Investigator

Brochure) will be used as the reference document for assessing the expectedness of the event with regard to cabozantinib.

The Investigator is responsible for reporting relevant SAEs to the relevant regulatory authorities, and participating investigators, in accordance with FDA regulations (21 Code of Federal Regulations [CFR] 312.32), ICH guidelines, European Clinical Trials Directive (Directive 2001/20/EC), and/or local regulatory requirements.

- These reports are to be filed utilizing the Form FDA 3500A (MedWatch Form) or a CIOMS-1 form;
- Exelixis Drug Safety group (or designee) will process and evaluate all SAEs as the reports are received. For each SAE received, Exelixis will make a determination as to whether the criteria for expedited reporting to relevant regulatory authorities have been met. Exelixis reserves the right to upgrade the Investigator assessment of an SAE based on Exelixis assessment.
- Institutions and Investigators shall promptly provide all information requested by Exelixis regarding all adverse events occurring during the conduct of the study.
- The Investigator is responsible for complying with all regulatory authority reporting requirements for the study that are applicable to the Principal Investigator of a clinical trial.
- The Investigator shall provide a copy of all responses to regulatory agency requests, periodic reports, and final study reports to Exelixis within one (1) business day of the submission.
- Exelixis will provide relevant product safety updates and notifications, as necessary. In the case of multi-center studies, it is the responsibility of the Principal Investigator to disseminate these updates to participating Investigators.
- Reporting of SAEs by the Investigator to his or her Institutional Review Board (IRB)/Ethics Committees (ECs) will be done in accordance with the standard operating procedures and policies of the IRB/EC. Adequate documentation must be maintained showing that the IRB/EC was properly notified.
- In the case of blinded studies, the treatment blind will be broken by the Principal Investigator and/or other necessary personnel prior to reporting an SAE that meets the criteria for expediting reporting to the Regulatory Authorities and to some central ECs. All personnel that are not involved in the unblinding and submission processes will remain blinded to the treatment assignment.

7.2 Adverse Events of Special Interest

Adverse events of special interest (AESIs) consist of immune-mediated AEs associated with ICIs, cases of potential DILI, and suspected transmission of an infectious agent by the study treatment.

AESIs will be reported to the Principal Investigator or designee using the SAE reporting form irrespective of whether the event is serious or nonserious; all AESIs must be reported within one (1) business day using the SAE process as described in Sections 7.1.3. and 7.6.2.

Guidance for management of immune-mediated AEs is provided in the protocol (Section 6.2.2 – 6.2.4) and can also be found in the local prescribing information for Nivolumab.

Adverse Events of Special Interest

Event
Cases of potential DILI that include an elevated ALT or AST in combination with either an elevated bilirubin or clinical jaundice, as defined by Hy's Law and based on the following observations:<br>• Treatment-emergent ALT or AST > 3x ULN in combination with total bilirubin > 2x ULN<br>• Treatment-emergent ALT or AST > 3x ULN in combination with clinical jaundice
Suspected transmission of an infectious agent by the study treatment, as defined below:<br><br>Any organism, virus, or infectious particle (e.g., prion protein transmitting transmissible spongiform encephalopathy), pathogenic or non-pathogenic, is considered an infectious agent. A transmission of an infectious agent may be suspected from clinical symptoms or laboratory findings that indicate an infection in a patient exposed to a medicinal product. This term applies only when a contamination of study treatment is suspected.
Pneumonitis
Colitis
Endocrinopathies: diabetes mellitus, pancreatitis, adrenal insufficiency, hyperthyroidism, and hypophysitis
Hepatitis, including AST or ALT > 10x ULN
Systemic lupus erythematosus
Neurological disorders: Guillain-Barré syndrome, myasthenic syndrome or myasthenia gravis, and meningoencephalitis
Events suggestive of hypersensitivity, infusion-related reactions, cytokine release syndrome, influenza-like illness, and systemic inflammatory response syndrome
Nephritis
Ocular toxicities (e.g., uveitis, retinitis)
Myositis
Myopathies, including rhabdomyolysis
≥ Grade 2 cardiac disorders (e.g., atrial fibrillation, myocarditis, pericarditis)
Vasculitis

7.2.1 General Information on Immune-Related Adverse Events

The immune-modulating properties of Nivolumab are able to undermine immunologic tolerance and generate a subset of AEs (called irAEs) with an autoimmmune inflammatory pathomechanism. Immune-related AEs may involve any organ or tissue .[23] Most irAEs occur within the first 12 weeks of exposure to IOs but some may appear with a delayed onset. Diagnosis of irAEs should be based on exposure to an IO and a reasonable immune-based mechanism of the observed AE. Whenever possible, histologic examination or other immune-based diagnostic evaluations should be used to support the diagnosis. Other etiologic causes including AEs from tumor progression should be ruled out.

The spectrum of irAEs is wide and can be general or organ-specific. Examples of general irAEs in subjects treated with IOs are fatigue, fever, and chills. Organ-specific irAEs consist of dermatitis (rash, pruritus, vitiligo, oral mucositis, and gingivitis), enterocolitis (diarrhea with abdominal pain and clinical or radiological evidence of colonic inflammation), and endocrinopathies (pituitary, thyroid, adrenal, testes). Diagnosis of endocrine dysfunction is challenging with relatively unspecific symptoms. The following additional laboratory testing of the endocrine axes may be helpful: prolactin (pituitary-hypothalamic function), FT4 and TSH (pituitary-thyroid function), luteinizing hormone (LH) and follicle-stimulating hormone (FSH; pituitary-gonadal function), adrenocorticotropic hormone (ACTH) and cortisol (pituitary-adrenal function).

Additional organ-specific irAEs include hepatitis (AST/ALT increases, hepatomegaly, periportal edema, periportal lymphadenopathy, lymphocyte infiltration of periportal tissue and surrounding primary biliary ducts) and pneumonitis (acute interstitial pneumonia). Less frequent irAEs include neurologic syndromes (myasthenia gravis, Guillian-Barré syndrome, meningoencephalitis), ocular AEs (uveitis), renal AEs (interstitial nephritis), cardiac AEs (myocarditis), and pancreatic AEs (lipase increase).

7.3 Follow-up of Adverse Events

Nonserious AEs will be followed until 30 days after the date of the decision to discontinue study treatment. All SAEs and AESIs (regardless of seriousness) will be followed until 100 days after the date of decision to discontinue study treatment.

All AESIs (regardless of seriousness) and all related SAEs that are ongoing 100 days after the date of the decision to discontinue study, and AEs assessed as related that led to study treatment discontinuation that are ongoing 100 days after the date of the decision to discontinue study treatment, are to be followed until either:

- The AE has resolved.
- The AE has improved to Grade 2 or lower.
- The investigator determines that the event has become stable or irreversible.

7.3.1 Unanticipated Problems Involving Risks to Subjects or Others

An unanticipated problem is any incident, experience, or outcome that **meets all three** of the following criteria:

1. Unexpected (in terms of nature, severity, or frequency) given the following: a) the research procedures described in the protocol-related documents such as the IRB approved research protocol, informed consent document or Investigator Brochure (IB); and b) the characteristics of the subject population being studied; **AND**
2. Related or possibly related to participation in the research (possibly related means there is a reasonable possibility that the incident, experience, or outcomes may have been caused by the drugs, devices or procedures involved in the research); **AND**
3. Suggests that the research places subjects or others at greater risk of harm (including physical, psychological, economic, or social harm) than previously known or recognized.

7.3.2 Adverse Events of Special Interest (AESI)

Specific adverse events, or groups of adverse events, will be followed as part of standard safety monitoring activities. These events, regardless of seriousness, will be reported.

7.3.2.1 Study specific AESIs

- **CBM588**

To date, clinical studies of CBM588 have not reported any adverse events. The potential side effects with probiotic therapies include abdominal pain, constipation, diarrhea, and fatigue.

- **Cabozantinib with Nivolumab**

With TKI/immunotherapy combination therapy, it is challenging to ascribe toxicity to either agent alone – as such, the collective side effects potentially associated with this regimen include but are not limited to:

- Diarrhea
- Palmar–plantar erythrodysesthesia
- Hypertension
- Hypothyroidism
- Fatigue
- Increased ALT level
- Decreased appetite
- Nausea
- Increased AST level
- Dysgeusia
- Asthenia
- Rash

- Mucosal inflammation
- Pruritus
- Arthralgia
- Back pain
- Vomiting
- Cough
- Dysphonia
- Stomatitis
- Increased lipase level
- Hyponatremia
- Abdominal pain
- Headache

7.4 Other Safety Considerations

7.4.1 Pregnancy/Lactation Exposure

Use of highly effective methods of contraception is very important during the study and must continue for 4 months after the last dose of cabozantinib, 5 months after the last dose nivolumab for women with childbearing potential, and 7 months after the last dose of nivolumab for men. If a subject becomes pregnant during the study, she will be taken off study treatment and will be followed through the end of her pregnancy and the infant should have follow up for at least 6 months after birth. If a female partner of a male subject becomes pregnant during the study, the Investigator will ask the pregnant female to consent to be followed through the end of her pregnancy and for the infant to be followed for at least 6 months after birth. Furthermore, male subjects must refrain from donating sperm in order to avoid transmission of study treatment in semen for the duration of study treatment and through 7 months after their last dose of study treatment. Both male and female subjects should seek advice and consider fertility preservation before receiving study treatment.

The Investigator must inform Exelixis of the pregnancy. Forms will be provided to the study sites upon request. The outcome of a pregnancy (for a subject or for the partner of a subject) and the medical condition of any resultant offspring must be reported to Exelixis. Any birth defect or congenital anomaly

must be reported as an SAE, and any other untoward events occurring during the pregnancy must be reported as AEs or SAEs, as appropriate.

Females should not breastfeed while receiving study treatment and for up to 4 months from the last dose of cabozantinib and up to 5 months after the last dose of nivolumab.

7.4.2 Medication Errors/Overdose

Medication error is defined as the administration of each study drug medication outside or above the established dosing regimens per the specific protocol. Any study medication overdose, misuse, abuse, or medication error (excluding missed doses) that results in an AE, even if it does not meet the definition of serious, requires reporting within one (1) business day to Exelixis.

In case of overdose, the Principal Investigator should be contacted promptly to discuss how to proceed. Any AEs that occur as a result of an overdose have to be treated according to clinical standard practice. Please refer to the cabozantinib Investigator Brochure for additional management recommendations regarding overdoses of cabozantinib.

Expected (known) toxicities to cabozantinib with nivolumab

Comprehensive Adverse Events and Potential Risks list (CAEPR) for XL184 (Cabozantinib, NSC 761968)

**Adverse Events with Possible Relationship to XL184 (Cabozantinib) (CTCAE 5.0 Term) [n= 3219]**			**Specific Protocol Exceptions to Expedited Reporting (SPEER)**
Likely (>20%)	Less Likely (<=20%)	Rare but Serious (<3%)	
BLOOD AND LYMPHATIC SYSTEM DISORDERS			
	Anemia		
ENDOCRINE DISORDERS			
	Hypothyroidism		Hypothyroidism (Gr 2)
GASTROINTESTINAL DISORDERS			
	Abdominal pain<br>Constipation		Abdominal pain (Gr 3)<br>Constipation (Gr 2)
Diarrhea			Diarrhea (Gr 3)
	Dry mouth<br>Dyspepsia		Dry mouth (Gr 2)<br>Dyspepsia (Gr 2)
		Gastrointestinal fistula2<br>Gastrointestinal hemorrhage3	
Nausea	Mucositis oral	Gastrointestinal perforation4	Mucositis oral (Gr 3)<br>Nausea (Gr 3)
Vomiting	Oral pain		Oral pain (Gr 2)<br>Vomiting (Gr 3)
GENERAL DISORDERS AND ADMINISTRATION SITE CONDITIONS			
Fatigue	Edema limbs		***Fatigue (Gr 3)***

INFECTIONS AND INFESTATIONS			
	Infection		
INJURY, POISONING AND PROCEDURAL COMPLICATIONS			
		Wound complication	
INVESTIGATIONS			
	Alanine aminotransferase increased<br>Aspartate aminotransferase increased<br>Lipase increased		Alanine aminotransferase increased (Gr 3)<br>Aspartate aminotransferase increased (Gr 3)<br>Lipase increased (Gr 4)
Weight loss	Platelet count decreased		Platelet count decreased (Gr 3)<br>Weight loss (Gr 3)
METABOLISM AND NUTRITION DISORDERS			
Anorexia	Dehydration		***Anorexia (Gr 3)***
	Hypocalcemia<br>Hypokalemia		
	Hypomagnesemia		
	Hypophosphatemia		

MUSCULOSKELETAL AND CONNECTIVE TISSUE DISORDERS			
	Arthralgia		
	Generalized muscle weakness<br>Muscle cramp	Osteonecrosis of jaw	
	Pain in extremity		
NERVOUS SYSTEM DISORDERS			
	Dizziness		
Dysgeusia			***Dysgeusia (Gr 2)***
	Headache	Intracranial hemorrhage	
		Ischemia cerebrovascular<br>Reversible posterior leukoencephalopathy syndrome<br>Stroke	
		Transient ischemic attacks	
RENAL AND URINARY DISORDERS			
	Hematuria		

		Proteinuria	
RESPIRATORY, THORACIC AND MEDIASTINAL DISORDERS			
	Cough<br>Dyspnea		
		Pneumothorax6<br>Respiratory fistula7	
	Respiratory hemorrhage8<br>Voice alteration		***Voice alteration (Gr 3)***
SKIN AND SUBCUTANEOUS TISSUE DISORDERS			
	Alopecia<br>Dry skin<br>Hair color changes		***Dry skin (Gr 2)***<br>***Hair color changes (Gr 1)***
Palmar-plantar erythrodysesthesia syndrome			***Palmar-plantar erythrodysesthesia syndrome (Gr 3)***
	Rash maculo-papular		***Rash maculo-papular (Gr 3)***
VASCULAR DISORDERS			
Hypertension			***Hypertension (Gr 3)***
	Thromboembolic event		

Adverse Events with Possible Relationship to BMS-936558 (Nivolumab, MDX-1106) (CTCAE 5.0 Term) [n= 2069]			Specific Protocol Exceptions to Expedited Reporting (SPEER)
Likely (>20%)	Less Likely (<=20%)	Rare but Serious (<3%)	
		Hyperglycemia	***Hyperglycemia (Gr 2)***
		Metabolism and nutrition disorders - Other (diabetes mellitus with ketoacidosis)[3]	
MUSCULOSKELETAL AND CONNECTIVE TISSUE DISORDERS			
	Arthralgia		
		Musculoskeletal and connective tissue disorder - Other (polymyositis)	
		Myositis	
		Rhabdomyolysis	
NERVOUS SYSTEM DISORDERS			
		Encephalopathy	
		Facial nerve disorder	
		Guillain-Barre syndrome	
		Myasthenia gravis	
		Nervous system disorders - Other (demyelination myasthenic syndrome)	
		Nervous system disorders - Other (encephalitis)	
		Nervous system disorders - Other (meningoencephalitis)	
		Nervous system disorders - Other (meningoradiculitis)	
		Nervous system disorders - Other (myasthenic syndrome)	
		Peripheral motor neuropathy	
		Peripheral sensory neuropathy	
		Reversible posterior leukoencephalopathy syndrome[3]	
RENAL AND URINARY DISORDERS			
		Acute kidney injury	
RESPIRATORY, THORACIC AND MEDIASTINAL DISORDERS			
	Pleural effusion		
	Pneumonitis		

		Respiratory, thoracic and mediastinal disorders - Other (bronchiolitis obliterans with organizing pneumonia)[3]	
SKIN AND SUBCUTANEOUS TISSUE DISORDERS			
		Erythema multiforme[3]	
**Adverse Events with Possible Relationship to BMS-936558 (Nivolumab, MDX-1106) (CTCAE 5.0 Term) [n= 2069]**			**Specific Protocol Exceptions to Expedited Reporting (SPEER)**
**Likely (>20%)**	**Less Likely (<=20%)**	**Rare but Serious (<3%)**	
	Pruritus		***Pruritus (Gr 2)***
	Rash maculo-papular		***Rash maculo-papular (Gr 2)***
		Skin and subcutaneous disorders - Other (bullous pemphigoid)	
	Skin and subcutaneous disorders - Other (Sweet's Syndrome)		
	Skin hypopigmentation		
		Stevens-Johnson syndrome	
		Toxic epidermal necrolysis	

7.5 Adverse Events and Unanticipated Problems

The research team is responsible for classifying AEs and UPs as defined in the relevant regulations and reporting to all applicable parties, including but not limited to the COH IRB, DSMC, Food and Drug Administration (FDA), National Institutes of Health (NIH) and other collaborators, e.g., pharmaceutical companies. The research team is responsible for the continued monitoring and tracking of all AEs in order to ensure non-reportable events are reviewed and monitored and do not rise to a reporting level.

7.6 Assessment of Adverse Events

The site Investigator will be responsible for determining the event name, and assessing the severity (i.e. grade), expectedness, and attribution of all adverse events as applicable per the City of Hope Clinical Research Adverse Event and Unanticipated Problem policy. Adverse events will be characterized using the descriptions and grading scales found in version 5.0 of Common Terminology Criteria for Adverse Events (CTCAE). A copy of the scale can be found at https://ctep.cancer.gov/protocolDevelopment/electronic_applications/ctc.htm#ctc_50.

The following definitions will be used to determine the causality (attribution) of the event to the study agent or study procedure.

- **Unrelated –** The event is clearly NOT related to study treatment and is clearly related to other factors such as the participant's clinical state, other therapeutic interventions, or concomitant medications administered to the participant.

- **Unlikely –** The event is unlikely related to the study treatment and is most likely related to other factors such as the participant's clinical state, other therapeutic interventions, or concomitant drugs.
- **Possible –** The event may be related to study treatment, as it follows a reasonable temporal sequence from the time of drug administration, but could have been produced by other factors such as the participant's clinical state, other therapeutic interventions, or concomitant drugs.
- **Probable –** The event is most likely related to the study treatment, as it follows a reasonable temporal sequence from the time of drug administration and a known response pattern to the study drug, and is unlikely related to the participant's clinical state, other therapeutic interventions, or concomitant drugs.
- **Definite –** The event is clearly related to the study treatment, as it follows a reasonable temporal sequence from the time of drug administration and a known response pattern to the study drug, and is not reasonably explained by other factors such as the participant's condition, therapeutic interventions, or concomitant drugs.

7.7 Reporting of Adverse Events

7.7.1 Routine Recording of Non-Serious Adverse Events

Routine recording of all grade of adverse events will occur via data entry into the study eCRF. Collection of adverse events will begin once the patient is consented and will continue until the patient completes, discontinues, or withdraws from the study. Adverse events will be monitored by the Protocol Management Team (PMT). Adverse events that do not meet the criteria of serious OR are not unanticipated problems do not require expedited reporting. AEs reported through expedited processes (i.e. reported to the IRB, DSMC, FDA, etc.) must also be reported in routine study data submissions.

7.7.2 Expedited Reporting Requirements of SAEs and UPs to the COH Regulatory Committees

Adverse events that meet the criteria of serious OR are unanticipated problems will be reported according to the approved City of Hope Clinical Research Adverse Event and Unanticipated Problem policy. Reporting of SAEs will begin once the patient is consented, and must be followed until the event is resolved, stabilized, or determined to be irreversible by the investigator. Follow-up SAE reports must be submitted for all events that require expedited reporting when the status of the event changes and until the resolution or stabilization of the event.

7.7.3 Additional AE Reporting Requirements

7.7.3.1 Reporting to the FDA

The study PI (or designee) will be responsible for contacting the Office of IND Development and Regulatory Affairs (OIDRA) at COH to ensure prompt reporting of safety reports to the FDA. OIDRA will assist the PI with the preparation of the report and submit the report to the FDA in accordance with the City of Hope Clinical Research Adverse Event and Unanticipated Problem policy.

Serious Adverse Events meeting the requirements for expedited reporting to the Food and Drug Administration (FDA), as defined in 21 CFR 312.32, will be reported as an IND safety report using the MedWatch Form FDA 3500A for Mandatory Reporting.

The criteria that require reporting using the Medwatch 3500A are:

- Any unexpected fatal or life threatening adverse experience associated with use of the drug must be reported to the FDA no later than 7 calendar days after initial receipt of the information [21 CFR 312.32(c)(2)]
- Any adverse experience associated with use of the drug that is both serious and unexpected must be submitted no later than 15 calendar days after initial receipt of the information [21 CFR 312.32(c)(1)]
- Any follow-up information to a study report shall be reported as soon as the relevant information becomes available. [21 CFR 312.32(d)(3)]

In addition, the study PI will submit annually within 60 days (via COH OIDRA) of the anniversary date of when the IND went into effect, an annual report to the FDA which is to include a narrative summary and analysis of the information of all FDA reports within the reporting interval, a summary report of adverse drug experiences, and history of actions taken since the last report because of adverse drug experiences.

7.7.3.2 Reporting to Industry partner/ funding source

All serious adverse events and AESIs (initial and follow-up information) will be reported by the study PI to Osel, Inc within 24 hours. Similarly, all serious adverse events and AESIs, must be reported to Exelixis within one (1) business day and per section 7.1.3 of this protocol.

8.0 AGENT INFORMATION

8.1 Nivolumab (NSC#748726); Opdivo

Product description:

Nivolumab will be supplied by City of Hope Comprehensive Cancer Center and billed to patients and/or their third-party payer.

Nivolumab injection is a clear opalescent, colorless to pale yellow, sterile, non-pyrogenic, single-use, isotonic aqueous solution formulated in sodium citrate, sodium chloride, mannitol, diethylenetriamine pentacetic acid (pentetic acid) and polysorbate 80 (Tween® 80), pH 6.0

Each vial is 100 mg (10 mg/mL) with a 0.7 mL overfill in 10 mL type I flint glass vials, with butyl rubber stoppers and aluminum seals

Solution preparation

Nivolumab can be infused undiluted (10 mg/mL) or diluted with 0.9% Sodium Chloride injection, USP or 5% dextrose, USP to drug concentrations no less than 0.35 mg/mL. Note: mix gently and do not shake. DO NOT administer as IV push or bolus injection.

Nivolumab injection is to be administered as a 30 minute IV infusion through a 0.2 micron to 1.2 micron pore size, low protein binding polyethersulfone membrane in-line filter.

Storage requirements

Vials of nivolumab injection must be stored at 2○C-8○C (36○F-46○F) and protected from light, freezing and shaking. If a storage temperature excursion is identified, promptly return Nivolumab to 2°C to 8°C and quarantine the supplies.

Stability:

No compatibilities between nivolumab and polyvinyl chloride (PVC), nonPVC DHEP (di(2-ethylhexyl)phthalate) IV components, or glass bottles have been observed.

The administration of undiluted and diluted solutions of Nivolumab must be completed within 24 hours preparation. If not used immediately, the infusion solution may be stored up to 24 hours in a refrigerator at 2○C-8○C (36○F-46○F) and a maximum of 4 hours of the total 24 hours can be at room temperature (20○C-25○C (68○F-77○F)) and room light. The maximum 4-hour period under room temperature and room light conditions includes the product administration period.

Route of administration:

Nivolumab injection is to be administered as a 30 minute IV infusion through a 0.2 micron to 1.2 micron pore size, low protein binding polyethersulfone membrane in-line filter.

The single-use dosage form contains no antibacterial preservative or bacteriostatic agent. Therefore, it is advised that the product be discarded 8 hours after initial entry.

No prophylactic medication should be given unless indicated by previous indication.

Availability:

Nivolumab is a commercial agent supplied by City of Hope Comprehensive Cancer Center and billed to patients and/or their third-party payer.

8.2 Cabozantinib (NSC #761968)

Product description:

Cabozantinib will be supplied by City of Hope Comprehensive Cancer Center and billed to patients and/or their third-party payer.

Cabozantinib tablets are supplied as film coated tablets containing cabozantinib malate equivalent to 40 mg of cabozantinib and contain microcrystalline cellulose, lactose anhydrous, hydoxypropyl cellulose, croscarmellose sodium, colloidal silicon dioxide, magnesium stearate and Opadry® yellow.

Storage requirements:

Store intact bottles at controlled room temperature, 20° to 25°C (68° to 77°F); temperature excursions are permitted between 15°and 30°C (59° to 86°F).

If a storage temperature excursion is identified, provide a detailed report of the excursion (including documentation of temperature monitoring and duration of the excursion) to Clinical_Supplies@exelixis.com for determination of suitability.

Stability:

Stability testing of the intact bottles is on-going. Cabozantinib is stable for up to 24 hours when dispensed in an open container, such as in a pill cup, and are stable for up to 7 days when dispensed in a closed container, such as a pharmacy bottle other than the original container.

Route of administration:

Participants will receive cabozantinib orally at a dose of 40mg once daily.

Cabozantinib tablets should only be taken whole by mouth and should not be cut, chewed, or crushed for dissolving in liquid or administered through other routes including percutaneous endoscopic gastrostomy (PEG) tubes. Cabozantinib tablets should not be administered to subjects who do not have adequate swallowing capacity. Cabozantinib should not be taken with food (subjects should not eat for at least 2 h before and at least 1 h after taking cabozantinib) and should be taken with a full glass (at least 8 ounces or 240 mL) of water. If a dose is missed, the missed dose should not be taken less than 12 h before the next dose.

Availability:

Cabozantinib is a commercial agent supplied by City of Hope Comprehensive Cancer Center and billed to patients and/or their third-party payer.

8.3 CBM588

Product description:

CBM588 will be supplied by Osel, Inc via the City of Hope Comprehensive Cancer Center Investigational Drug Pharmacy free of charge.

CBM588 fine granules are manufactured under cGMP at Miyarisan Pharmaceutical Company, Ltd. in Nagano, Japan. The product contains the bacterium *Clostridium butyricum* MIYAIRI 588, lactose, calcium carbonate, and corn starch. The fine granule preparation is light white-gray in color and has a characteristic odor and sweet taste. Each gram of fine granules contains 40 mg of CBM588 Powder, the active pharmaceutical ingredient, and 2 x 10^8 CFU of *C. butyricum*. CBM588 is packaged in cellophane polyethylene sachets containing 1 g of fine granules per sachet.

Storage requirements:

It is recommended that CBM588 be stored at controlled room temperature: the temperature maintained thermostatically that encompasses at the usual and customary working environment of 20°-25° (68°-77 °F). Excursions between 15° and 30° (59° and 86 °F) that are experienced in pharmacies, hospitals, and warehouses, and during shipping are allowed.

Stability:

CBM588 fine granules are stable for at least 18 months at 25 ± 2° C (77 ± 4° F) and 60 ± 5 % relative humidity and tested for appearance, particle size, identification, purity, loss on drying, and potency. The stability of all clinical product lots will be monitored for the duration of the clinical trial.

Route of administration:

CBM588 should be administered orally 2 times per day. Two sachets of CBM588 fine granules (2g) will be mixed with 100 ml of water and administered orally twice daily (morning and evening) for the duration of the study. The granules immediately dissolve in water but form a cloudy solution due to the presence of precipitated calcium carbonate in the formulation.

Availability:

CBM588 is a commercial product in Japan manufactured by Miyarisan Pharmaceutical Co. Ltd. It is supplied as an investigational drug in the United States by Osel, Inc through the Investigational Drug Pharmacy at City of Hope Comprehensive Cancer Center.

9.0 CORRELATIVE/ SPECIAL STUDIES

9.1 Assessment of the Stool Microbiome

Samples will be collected at pre-specified time points as outlined in the study calendar, and specific instructions for stool collection will be supplied to the patient as per **Appendix B**. The following delineates methods of assessment.

9.1.1 Stool Collection

Fecal material will be collected using the OMNIgene Gut collection kit by patients at two time points, before starting treatment (baseline) and at week 12, or 7 days thereafter. . A standard operating procedure (SOP) has been generated for stool collection, as outlined in Appendix B. Stool collection kit contents are listed in Appendix B. A copy of this SOP will be provided to the patient and their understanding of the SOP will be documented by the PI.

All samples will be collected by participants at home and dropped to a FedEx location on the day of sample collection and prior to that day's final delivery. The sample will arrive at Dr. David Engelthaler's laboratory at TGen North, to the attention of Michael Valentine (3051 W. Shamrell Blvd, Suite 106, Flagstaff, AZ, 86005) the next day.

9.1.2 Laboratory Processing and Analysis

Bacterial microbiome analysis

Stool mailed to City of Hope/TGen North will be processed and analyzed in the laboratory of Dr. David Engelthaler. Genomic DNA will be extracted from 0.5 g of stool per subject. Lysis buffer (5 mL, Perkin Elmer 852) will be added to each stool sample and vortexed until homogenous. Homogenized sample (1.2 mL) and Proteinase K enzyme (15 µM, Sigma Aldrich, PN. P2308) enzyme will be aliquoted to a 2 mL tube with garnet beads (Mo Bio PN. 12830-50-BT). Bead tubes with 1.2 mL of specimen will then be incubated at 65 °C for 10 min and then at 95 °C for 10 min.

Tubes will then be placed on a Vortex Genie 2 to perform bead beating for 10 min and the sample will subsequently be spun in an Eppendorf Centrifuge 5424 at maximum speed. Supernatant (700 µL) will then be transferred to a deep well block. DNA extractions and purifications will be performed using a Chemagic MSM I (Perkin Elmer) following the manufacturer's protocol. Samples will then be further purified using the Onestep Inhibitor Removal kit following manufacturer's instructions (Zymo Research PN. D6035). DNA samples will be quantified using Quant-iT on an Eppendorf AF2200 plate reader. Samples will be assessed for DNA integrity by agarose gel electrophoresis. Only non-degraded samples with high molecular weight DNA will be used for sequencing.

Paired-end DNA libraries with insertion size of 350 bp will be prepared using the NexteraXT library preparation kit (Illumina, San Diego, CA). Paired-end sequencing will be conducted using an Illumina NextSeq 500 sequencer at JCVI with 150 bp read length. Reads will be pre-processed following the standard protocol in the JCVI Sequencing Core then further processed using Trimmomatic 0.33 with recommended parameters to remove short, low-quality, and adapter-contaminated reads. To eliminate human reads, data will then be mapped to the Human Reference Genome Release 107 (Genome Reference Consortium) using Bowtie2 v2.2.5 with default parameters. Mapped human reads will be discarded from the pool.

Processed paired-end WGS reads will be subject to de novo metagenome assembly using IDBA-UD 1.1.1. Contigs shorter than one kb will be dropped from the pool. The quality of assembly will be assessed using Quast 2.3 and a series of in-house Python scripts. Original reads will be mapped backed to the contigs using Bowtie2, and the read coverage of each contig will be calculated using the “genomecov” command implemented in BEDTools v2.24.0. Circularizable contigs will be identified based on the presence of repeated sequences on of both ends.[24]

Fungal microbiome analysis

DNA will be extracted from stool samples using the MagMax PowerMicrobiome extraction kit using the KingFisher Flex magnetic purification system (ThermoFisher, Waltham, MA). All DNAs will be validated for purity and integrity by agarose gel electrophoresis, and fungal load will be quantitated using the FungiQuant TaqMan assay before proceeding to sequencing. DNAs will be subjected to internally transcribed spacer gene sequencing using the)ITS4-Fun AGCCTCCGCTTATTGATATGCTTAART and 5.8S-Fun AACTTTYRRCAAYGGATCWCT primers with dual-indexed bar codes as described by Kozich et al.[25–27] A DNA mock community that contains two fungal species will be sequenced as a positive control (Zymo, D6306) as will an extraction blank as a negative control. Libraries will be quantitated using KAPA Library Quantification Kit (KAPA Biosystems), normalized and pooled then sequenced on the Illumina MiSeq instrument using the v2 kit (2 x250 bp) targeting 20,000 reads per sample.

Demultiplexed ITS reads will be processed and clustered into operational taxonomic units (OTUs) using QIIME2.[28] Demultiplexed reads will be denoised using dada2 then clustered into OTUs using q2-dbotu. Taxonomic classification will be performed using feature-classifier classify-sklearn. Various QIIME2 plugins will be used to build a phylogenetic tree (phylogeny), calculate alpha diversity within samples (diversity plugin) and beta diversity between samples (diversity plugin). We have found that simple alpha diversity calculations, such as inverse Simpson diversity, Shannon and Chao estimates, and examination of the dominant genera in each sample provides a good first impression of the composition of the sample. We will use several different types of distance methods (Bray Curtis, UniFrac) to examine and compare the community distribution of the samples. These data will be examined by multidimensional scaling or principal components analysis and heatmaps will be created using hclust2.

Whole metagenome sequencing provides a complete picture of the taxonomic composition of a microbiome than does 16S rRNA gene or ITS sequencing and also permits predictions of microbiome function, which we can tie to metabolic and proteomic results. Metagenomics also has been used for the discovery of clinical biomarkers and will allow us to identify antibiotic resistance genes. Metagenomic DNA will be sequenced on the Illumina NextSeq platform to a depth of 2 Gb/sample.[29,30] Human reads will be identified by mapping them to the human genome GRCh38.p7 using BowTie2 and they will be removed. Demultiplexed reads will be quality trimmed using Trimmomatic to remove adapters and low-quality bases and reads.[8,31] Trimmed metagenomic reads will be taxonomically profiled using MetaPhlAn 2.0. Functional profiling of the metagenomes will be performed using HUMAnN2, which annotates open reading frames (ORFs) and generates gene family abundances, metabolic pathway coverage and abundances. Antimicrobial resistance genes will be identified by mapping to a curated version of the Resfams database. Resistance genes of interest will be verified by targeted amplicon sequencing.[9,10,32,33]

9.2 Assessment of Serum Cytokines

9.2.1 Research Blood Specimen Collection and Transfer to Processing Laboratory

One 10 mL CPT tube will be collected within 7 days before start of nivolumab and cabozantinib, during weeks 9, 13, 17 and 25 (+/- 1 week). The pre-treatment sample may be collected on the morning of initiation of therapy, so long as the sample precedes cabozantinib/nivolumab administration. Efforts will be made to collect the sample at the time of routine blood sample collection. Blood will be collected into 10 mL CPT vacuum tube, inverted slowly about 8-10 times, maintained at room temperature.

The sample will be labeled with the date, time of collection, IRB # ADD, and the participant's unique research participant number, prior to the prompt transport to the laboratory of Dr. Marcin Kortylewski for correlative analyses.

The Kortylewski laboratory should be notified of planned samples via email (mkortylewski@coh.org) or other laboratory designee preferably at least a day in advance of sample collection.

9.2.2 Initial Sample Processing – Isolation of PBMCs

Processing of samples will occur at in the laboratory of Dr. Marcin Kortylewski. The 10 mL CPT tube samples will be processed ASAP, ideally within a window of 4-6 hours. CPT tubes will be centrifuged at 1800 x g (approximately 2800 rpm on a Sorvall RT6000 centrifuge) for 20 minutes at room temperature. After centrifugation, plasma in the CPT tubes will be gently pipetted against the gel plug to dislodge cells stuck to the top of the gel. The cell suspension will be transferred to a 50 mL conical polypropylene tube. cRPMI will be added to a total of 40 mL. A 10 mL aliquot of cell suspension for counting will be removed. The 50 mL tubes will then be centrifuged at 250 x g for seven minutes at room temperature. When centrifugation is complete, the supernatant will be aspirated. PMBCs will be either cryopreserved or used fresh.

9.2.3 Sample Analysis

Analysis of samples for T-cell subpopulations will occur in the laboratory of Dr. Marcin Kortylewski. Relevant WBC subsets will be conducted through previously reported techniques. PBMCs will be immersed in a mixture of PBS, 2% FCS and 0.1% (wt/vol) sodium azide with Fc III/IIR-specific antibody to block nonspecific binding and stained the cells with different combinations of fluorochrome-coupled antibodies to CD11c, I-Ab (MHC class II), CD86, CD11b, Gr1, CD49b, CD3, CD25 or Lag-3, or with annexin V (BD Biosciences). We will collect fluorescence data on FACSCalibur (Beckton Dickinson) and analyze them using FlowJo software (Tree Star). This method has been previously published by Chalmin et al. [16]

9.3 Correlative Banking

Specimens collected at screening and during treatment will be deidentified and banked for future studies. Blood samples will be stored in the laboratory of Dr. Marcin Kortylewski at City of Hope and stool/fecal DNA samples will be stored at TGen North at Shamrell Blvd, Suite 106, Flagstaff, AZ, 86005. The banked samples will be stored indefinitely.

10.0 STUDY CALENDAR

	Screening	Pre-tx	Nivolumab/ Cabozantinib/CBM588						
			Wk 1	Wk 5	Wk 9	Wk 13	Wk 17	Wk 21	Wk 25+*
Informed consent	X								
Eligibility review	X								
Nivolumab			X	X	X	X	X	X	X[b]
Cabozantinib			X ---Xb						
CBM588			X ---Xb						
Registration	X								
Participant orientation		X							
Research blood collection		X			X	X	X		X
Planned stool specimens		X				X***			
Study log review/collection			X	X	X	X	X	X	X[b]
Data collection					X	X	X		X[b]
CT Chest/Abdomen/Pelvis	X[a]					X**			X[b]
Bone scan	X[c]					X**			X[b]
MRI or CT of Brain	X[d]								
Physical examination	X[e]		X	X	X	X	X	X	X
Safety Laboratory Assessments	X[f]		X	X	X	X	X	X	X

[a] Screening imaging should be completed within 6 weeks of registration. [b] Treatment administration, data collection, and response assessment should continue on 12-weekly intervals unless the treatment regimen is discontinued. [c] If indicated by the presence of bony metastasis at baseline.
[d] Brain imaging should be performed if patients are symptomatic or have a prior history of CNS metastases. See Section 3.2 for criteria related to enrollment for patients with brain metastases. [e] Routine physical examination during clinic visit with the oncologist or nurse practitioner. [f] Screening phase safety labs are per standard of care (i.e. the following labs are due at baseline: CBC+diff, CMP, LDH, TSH, FT4, cortisol); treatment phase safety labs are per standard of care (i.e. the following labs are due on D1 of each cycle: CBC+diff, CMP, LDH, TSH, FT4, cortisol).

11.0 *VITAL STATUS SHOULD BE ASSESSED EVERY 12 WEEKS FOLLOWING COMPLETION OF PROTOCOL-BASED THERAPY UNTIL TIME OF DEATH OR FORMAL WITHDRAWAL FROM STUDY, WHICHEVER COMES FIRST.**IMAGING WILL BE PERFORMED AT WEEK 13 (+/- 7 DAYS)***STOOL SPECIMEN WILL BE COLLECTED AT WEEK 12, OR 7 DAYS THEREAFTER ENDPOINT DEFINITIONS/MEASUREMENT OF EFFECT

11.1 Change in *Bifidobacterium* composition of stool from baseline to week 12 of therapy

Methods of assessment for stool microbiome composition are noted in Section 9.1. We will assess the proportion *Bifidobacterium spp* at baseline (relative to the cumulative assessment of microbial species) and compare this to the proportion observed after completion of 12 weeks of therapy.

11.2 Best overall response, by RECIST criteria

Response is a secondary endpoint in this trial. For this purpose of this study, patients should be re-evaluated for response every 12 weeks. Response and progression will be evaluated in this study using the new international criteria proposed by the revised Response Evaluation Criteria in Solid Tumors (RECIST) guideline (version 1.1).[34] The published RECIST document is available at http://www.eortc.be/RECIST. Changes in the largest diameter (unidimensional measurement) of the tumor lesions and the shortest diameter in the case of malignant lymph nodes are used in the RECIST criteria.

11.2.1 Definitions

Evaluable for toxicity. All patients will be evaluable for toxicity from the time of their first treatment with nivolumab plus cabozantinib alone or in combination with CBM588

Evaluable for objective response. Only those patients who have measurable disease present at baseline, have received at least one cycle of therapy, and have had their disease re-evaluated will be considered evaluable for response. These patients will have their response classified according to the definitions stated below. (Note: Patients who exhibit objective disease progression prior to the end of cycle 1 will also be considered evaluable.)

Evaluable Non-Target Disease Response. Patients who have lesions present at baseline that are evaluable but do not meet the definitions of measurable disease, have received at least one cycle of therapy, and have had their disease re-evaluated will be considered evaluable for non-target disease. The response assessment is based on the presence, absence, or unequivocal progression of the lesions.

11.2.2 Disease Parameters

Measurable disease. Measurable lesions are defined as those that can be accurately measured in at least one dimension (longest diameter to be recorded) as ≥20 mm by chest x-ray or as ≥10 mm with CT scan, MRI, or calipers by clinical exam. All tumor measurements must be recorded in millimeters (or decimal fractions of centimeters).

Tumor lesions that are situated in a previously irradiated area are considered measurable.

Malignant lymph nodes. To be considered pathologically enlarged and measurable, a lymph node must be ≥15 mm in short axis when assessed by CT scan (CT scan slice thickness recommended to be no greater than 5 mm). At baseline and in follow-up, only the short axis will be measured and followed.

Non-measurable disease. All other lesions (or sites of disease), including small lesions (longest diameter <10 mm or pathological lymph nodes with ≥10 to <15 mm short axis), are considered non-measurable disease. Bone lesions, leptomeningeal disease, ascites, pleural/pericardial effusions, lymphangitis cutis/pulmonitis, inflammatory breast disease, and abdominal masses (not followed by CT or MRI), are considered as non-measurable.

Target lesions. All measurable lesions up to a maximum of 2 lesions per organ and 5 lesions in total, representative of all involved organs, should be identified as target lesions and recorded and measured at baseline. Target lesions should be selected on the basis of their size (lesions with the longest diameter), be representative of all involved organs, but in addition should be those that lend themselves to reproducible repeated measurements. It may be the case that, on occasion, the largest lesion does not lend itself to reproducible measurement in which

circumstance the next largest lesion, which can be measured reproducibly should be selected. A sum of the diameters (longest for non-nodal lesions, short axis for nodal lesions) for all target lesions will be calculated and reported as the baseline sum diameters. If lymph nodes are to be included in the sum, then only the short axis is added into the sum. The baseline sum diameters will be used as reference to further characterize any objective tumor regression in the measurable dimension of the disease.

Non-target lesions. All other lesions (or sites of disease) including any measurable lesions over and above the 5 target lesions should be identified as non-target lesions and should also be recorded at baseline. Measurements of these lesions are not required, but the presence, absence, or in rare cases unequivocal progression of each should be noted throughout follow-up.

11.2.3 Methods for Evaluation of Measurable Disease

All measurements should be taken and recorded in metric notation using a ruler or calipers. All baseline evaluations should be performed as closely as possible to the beginning of treatment and never more than 4 weeks before the beginning of the treatment.

The same method of assessment and the same technique should be used to characterize each identified and reported lesion at baseline and during follow-up. Imaging-based evaluation is preferred to evaluation by clinical examination unless the lesion(s) being followed cannot be imaged but are assessable by clinical exam.

Clinical lesions: Clinical lesions will only be considered measurable when they are superficial (e.g., skin nodules and palpable lymph nodes) and ≥10 mm diameter as assessed using calipers (e.g., skin nodules). In the case of skin lesions, documentation by color photography, including a ruler to estimate the size of the lesion, is recommended.

Chest x-ray: Lesions on chest x-ray are acceptable as measurable lesions when they are clearly defined and surrounded by aerated lung. However, CT is preferable.

Conventional CT and MRI: This guideline has defined measurability of lesions on CT scan based on the assumption that CT slice thickness is 5 mm or less. If CT scans have slice thickness greater than 5 mm, the minimum size for a measurable lesion should be twice the slice thickness. MRI is also acceptable in certain situations (e.g. for body scans).

Use of MRI remains a complex issue. MRI has excellent contrast, spatial, and temporal resolution; however, there are many image acquisition variables involved in MRI, which greatly impact image quality, lesion conspicuity, and measurement. Furthermore, the availability of MRI is variable globally. As with CT, if an MRI is performed, the technical specifications of the scanning sequences used should be optimized for the evaluation of the type and site of disease. Furthermore, as with CT, the modality used at follow-up should be the same as was used at baseline and the lesions should be measured/assessed on the same pulse sequence. It is beyond the scope of the RECIST guidelines to prescribe specific MRI pulse sequence parameters for all scanners, body parts, and diseases. Ideally, the same type of scanner should be used and the image acquisition protocol should be followed as closely as possible to prior scans. Body scans should be performed with breath-hold scanning techniques, if possible.

PET-CT: At present, the low dose or attenuation correction CT portion of a combined PET-CT is not always of optimal diagnostic CT quality for use with RECIST measurements. However, if the site can document that the CT performed as part of a PET-CT is of identical diagnostic quality to a diagnostic CT (with IV and oral contrast), then the CT portion of the PET-CT can be used for RECIST

measurements and can be used interchangeably with conventional CT in accurately measuring cancer lesions over time. Note, however, that the PET portion of the CT introduces additional data which may bias an investigator if it is not routinely or serially performed.

Ultrasound: Ultrasound is not useful in assessment of lesion size and should not be used as a method of measurement. Ultrasound examinations cannot be reproduced in their entirety for independent review at a later date and, because they are operator dependent, it cannot be guaranteed that the same technique and measurements will be taken from one assessment to the next. If new lesions are identified by ultrasound in the course of the study, confirmation by CT or MRI is advised. If there is concern about radiation exposure at CT, MRI may be used instead of CT in selected instances.

11.2.4 Response Criteria

11.2.4.1 Evaluation of Target Lesions

Complete Response (CR): Disappearance of all target lesions. Any pathological lymph nodes (whether target or non-target) must have reduction in short axis to <10 mm.

Partial Response (PR): At least a 30% decrease in the sum of the diameters of target lesions, taking as reference the baseline sum diameters.

Progressive Disease (PD): At least a 20% increase in the sum of the diameters of target lesions, taking as reference the smallest sum on study (this includes the baseline sum if that is the smallest on study). In addition to the relative increase of 20%, the sum must also demonstrate an absolute increase of at least 5 mm. (Note: the appearance of one or more new lesions is also considered progressions).

Stable Disease (SD): Neither sufficient shrinkage to qualify for PR nor sufficient increase to qualify for PD, taking as reference the smallest sum diameters while on study.

11.2.4.2 Evaluation of Non-Target Lesions

Complete Response (CR): Disappearance of all non-target lesions. All lymph nodes must be non-pathological in size (<10 mm short axis).

Non-CR/Non-PD: Persistence of one or more non-target lesion(s) and/or maintenance of tumor marker level above the normal limits.

Progressive Disease (PD): Appearance of one or more new lesions and/or unequivocal progression of existing non-target lesions. Unequivocal progression should not normally trump target lesion status. It must be representative of overall disease status change, not a single lesion increase.

Although a clear progression of “non-target” lesions only is exceptional, the opinion of the treating physician should prevail in such circumstances, and the progression status should be confirmed at a later time by the review panel (or Principal Investigator).

11.2.4.3 Evaluation of Best Overall Response

The best overall response is the best response recorded from the start of the treatment until disease progression/recurrence (taking as reference for progressive disease the smallest measurements recorded since the treatment started). The patient's best response assignment will depend on the achievement of both measurement and confirmation criteria.

11.3 Duration of time from enrollment to progression

PFS is defined as the duration of time from start of treatment to time of progression or death, whichever occurs first.

11.4 Comparison of the Shannon index (a measure of microbial diversity) from baseline to week 12 of therapy

Using translational methods described in Section 9.1, we will compute the Shannon index at baseline and at week 12 for a comparison of microbial diversity at these two timepoints.

11.5 Comparison of the proportion of circulating Tregs at baseline to levels of circulating Tregs on treatment

Using translational methods described in Section 9.2, we will estimate the proportion of Tregs in the blood. This will be assessed graphically across serial timepoints of blood collection (see Study Calendar) to ascertain any trends.

11.6 Comparison of the proportion of circulating MDSCs at baseline to levels of circulating MDSCs on treatment

Using translational methods described in Section 9.2, we will estimate the proportion of MDSCs in the blood. This will be assessed graphically across serial timepoints of blood collection (see Study Calendar) to ascertain any trends.

11.7 Comparison of IL-6, IL-8 and other cytokines at baseline to levels of the same cytokines on treatment

Using translational methods described in Section 9.2, we will estimate the proportion of serum cytokines in the blood. This will be assessed graphically across serial timepoints of blood collection (see Study Calendar) to ascertain any trends.

12.0 STATISTICAL CONSIDERATIONS

12.1 Study Design

This is a randomized study of cabozantinib/nivolumab alone or in combination with CBM588. The objective is to define the biologic effect of CBM588 when used in combination with cabozantinib/nivolumab. Our preclinical data suggests that *Bifidobacterium spp* are associated with responses to immunotherapy, and we hypothesize that CBM588 will increase levels of *Bifidobacterium spp*. We will compare the proportional increase in *Bifidobacterium spp* with the addition of CBM588 to cabozantinib/nivolumab to patients receiving cabozantinib/nivolumab alone.

12.2 Sample Size and Accrual Rate

We will randomize 30 patients in a 1:2 fashion to receive cabozantinib/nivolumab alone [Arm 1] or with CBM588 [Arm 2]. We anticipate accrual of 30 patients over a 2-year span (approximately 1.5 patients per month), with approximately 16 months of follow-up on average (based on PFS estimates for cabozantinib/nivolumab). Given an anticipated 80% rate of consent to this study based on existing studies in patients with newly diagnosed mRCC, we would have to approach approximately 2 patients per month. This is feasible with current rates of new patient volume at our institution.

12.3 Statistical Analysis Plan

Primary and secondary endpoint analysis will be based on the intent-to-treat (ITT) population; that is, all patients who are randomized and signed the informed consent. All subjects will be analyzed according to the therapy arm to which they have been randomized.

Primary Endpoint

- (1) Change in Bifidobacterium composition of stool from baseline to week 12 of therapy.
- Analysis plan: Change in the *Bifidobacterium* from baseline to week 12 will be assessed for patients on both arms. With 20 on the CBM588 containing arm, and 10 on the non-CBM588 containing arm, we will have 80% power to detect a 1 standard deviation (common standard deviation of the change in *Bifidobacterium)* difference between the mean change detected in the two groups using a two-group t-test with a one-sided type I error of 0.05.

Secondary Endpoints

- (1a) Comparison of the Shannon index (a measure of microbial diversity) from baseline to week 12 of therapy will be conducted in a similar fashion. As this is a secondary measure, any conclusions will discuss the multiple comparison issue inherent in this second analysis.
- (1b) Best overall response, by RECIST criteria, with cabozantinib/nivolumab alone versus cabozantinib/nivolumab with CBM588.

Analysis plan: The association between treatment arm and overall response as per RECIST criteria (response observed vs not observed) will be examined using Fisher's exact test

- (1c) Progression-free survival (PFS), assessed as the duration of time from enrollment to progression, with cabozantinib/nivolumab alone versus cabozantinib/nivolumab with CBM588

Analysis plan: The difference in progression free survival across the two groups will be explored graphically using Kaplan-Meier survival plots. Median progression-free survival time for each of the two arms will be reported and Cox Proportional Hazards model will be used to estimate the hazard ratio and its confidence interval.

We will also conduct an exploratory analysis of the following, using the Wilcoxon signed rank test with no adjustment for the multiple comparison issue although any conclusion will include a discussion of the limitations of any conclusions drawn due to the multiple comparisons concern:

- (2a) Comparison of the proportion of circulating Tregs at baseline to levels of circulating Tregs with cabozantinib/nivolumab alone versus cabozantinib/nivolumab with CBM588
- (2b) Comparison of the proportion of circulating MDSCs with cabozantinib/nivolumab alone versus cabozantinib/nivolumab with CBM588
- (2c) Comparison of IL-6, IL-8 and other cytokines/chemokines with cabozantinib/nivolumab alone versus cabozantinib/nivolumab with CBM588.
- (3) Comparison of toxicities such as diarrhea and nausea using CTCAE v5 criteria with cabozantinib/nivolumab alone versus cabozantinib/nivolumab with CBM588.

- (4) Comparison of metabolic pathways at baseline and week 12 of treatment, among patients receiving cabozantinib/nivolumab alone versus cabozantinib/nivolumab with CBM588.

Safety Endpoints/Early Stopping:

- In Checkmate 9ER, 3/261 patients died due to treatment-related complications (~1%). As a result, any treatment-related death on the combination arm with CBM588 will hold the study pending detailed review and an evaluation of the cause of the toxicity, with accrual allowed to continue pending approval of the COH DSMC. A second treatment-related death on the CBM588 arm closes the study to further accrual.

13.0 PROTOCOL DEVIATIONS AND SINGLE SUBJECT EXCEPTIONS

Deviations from the protocol should be avoided, except when necessary to eliminate immediate hazard(s) for the protection, safety, and well-being of a research participant. As a result of deviations, corrective actions are to be developed by the study staff and implemented promptly. All protocol deviations and planned protocol deviations will be reported in accordance with the Clinical Research Protocol Deviation policy.

13.1 Single Subject Exceptions (SSE)

An SSE is a planned deviation, meaning that it involves circumstances in which the specific procedures called for in a protocol are not in the best interests of a specific patient. It is a deviation that is anticipated and receives prior approval by the Principal Investigator and the COH IRB.

14.0 CONDITIONS FOR MODIFYING THE PROTOCOL

Protocol modifications (including protocol amendments) may be made and will be prepared, reviewed, and approved by representatives of the Principal Investigator. Protocol modifications or amendments must be reviewed and approved by Exelixis prior to implementation.

All protocol modifications must be submitted to the IRB/EC for information and approval in accordance with local requirements and to regulatory agencies if required. Approval must be obtained before any changes can be implemented, except for changes necessary to eliminate an immediate hazard to study subjects or those that involve only logistical or administrative aspects of the trial (e.g., change in monitor or change of telephone number).

In addition, if contractually obligated, the sponsor must also approve the deviation.

15.0 STUDY OVERSIGHT, QUALITY ASSURANCE, AND DATA & SAFETY MONITORING

15.1 All Investigator Responsibilities

An investigator is responsible for ensuring that an investigation is conducted according to the signed investigator statement, the investigational plan, and applicable regulations; for protecting the rights, safety, and welfare of subjects under the investigator's care; and for the control of drugs under investigation.

15.2 Study Principal Investigator Responsibilities

The Study Principal Investigator is responsible for the conduct of the clinical trial, including overseeing that sponsor responsibilities are executed in accordance with federal regulations.

15.3 Protocol Management Team (PMT)

The Protocol Management Team (PMT), minimally consisting of the study PI, collaborating investigators, research nurse, clinical research associate/coordinator, and the study biostatistician, is responsible for ongoing monitoring of the data and safety of this study, including implementation of the stopping rules for safety/toxicity.

The PMT is recommended to meet (in person or via teleconference) to review study status. The meeting is a forum to discuss study related issues including accrual, SAE/AE/UPs experienced, study response, deviations/violations, and study management issues. The appropriateness of further subject enrollment and the specific intervention for subsequent subject enrollment are addressed.

15.4 Quality Assurance

Clinical site monitoring is conducted to ensure that the rights of human subjects are protected, that the study is implemented in accordance with the protocol and regulatory requirements, and that the quality and integrity of study data and data collection methods are maintained. Monitoring for this study will be performed by the City of Hope Office of Clinical Trials Monitoring (OCTM), within City of Hope's Office for Safety and Data Quality.

Details of clinical site monitoring are documented in the OCTM SOP and the Risk Based Monitoring (RBM) plan. These documents specify the frequency of monitoring, monitoring procedures, the amount of subject data to be reviewed, and the distribution of monitoring reports to the study team and the COH DSMC.

15.5 Risk Determination

This is a high risk study, as defined in the City of Hope Institutional DSMP. This determination was made because the study involves a COH held IND.

15.6 City of Hope Data and Safety Monitoring Committee

The COH Data and Safety Monitoring Committee (DSMC) will review and monitor study progress, compliance, toxicity, safety, and accrual data from this trial via the PMT Progress Report (submitted by the Study Principal Investigator according to the frequency outlined in the City of Hope Institutional DSMP). The DSMC is composed of clinical specialists who have no direct relationship with the study. Information that raises any questions about participant safety will be addressed with the Protocol Management Team.

16.0 CONDITIONS FOR TERMINATING THE STUDY

At any time, the study may be terminated by the Principal Investigator, the Principal Investigator's institution (City of Hope National Medical Center), or by Exelixis. Should this be necessary, Exelixis and the Principal Investigator will arrange the procedures on an individual study basis after review and consultation. In terminating the study, Exelixis and the Principal Investigator will ensure that adequate consideration is given to the protection of the subjects' interests. Upon study termination, the Principal Investigator and all Investigator(s) shall cease enrolling subjects into the study and shall discontinue conduct of the study as soon as is medically practicable.

17.0 ETHICAL AND REGULATORY CONSIDERATIONS

17.1 Patient Protection

The responsible investigator will ensure that this study is conducted in agreement with either the Declaration of Helsinki (Tokyo, Venice, Hong Kong, Somerset West and Edinburgh amendments) or the laws and regulations of the country, whichever provides the greatest protection of the patient.
The protocol has been written, and the study will be conducted according to the principles of Guideline for Good Clinical Practice.

(ref:http://www.ich.org/fileadmin/Public_Web_Site/ICH_Products/Guidelines/Efficacy/E6_R1/Step4/E6_R1__Guideline.pdf)

The protocol will be approved by local, centralized, regional or national ethics committees / institutional review boards.

17.2 Subject Identification

This research will be conducted in compliance with federal and state of California requirements relating to protected health information (PHI).

All samples will be coded prior to submission to the research laboratories. The coded identifier will be the COH research patient number (RPN), provided by the OnCore system, which is devoid of direct participant identifiers. The key to the code is maintained in OnCore which is a secure environment.
All study related forms including consent documents and patient diaries will be stored in locked and secure locations.

Medical records of participants will be securely maintained in the strictest confidence, according to current legal requirements. All information will be treated confidentially. No identifiers will be used in any subsequent publication of these results.

17.3 Informed Consent

All participants will undergo standard written informed consent procedures as dictated by the City of Hope Human Research Protections Office prior to performing any screening procedures that are not part of standard-of-care. Informed consent will be obtained by the principal investigator, collaborating investigators, or other IRB designated personnel who will meet the training requirements established by the IRB.

In addition, they will review the experimental subject's bill of rights and the HIPAA research authorization form.

All patients will be informed of the aims of the study, the possible adverse events, the procedures and possible hazards to which he/she will be exposed, and the mechanism of treatment allocation. They will be informed as to the strict confidentiality of their patient data, but that their medical records may be reviewed for trial purposes by authorized individuals other than their treating physician. An example of a patient informed consent statement is given as an appendix to this protocol.

Prospective research participants will be afforded sufficient time to consider whether or not to participate in the research.

It is the responsibility of the individual investigator to translate the enclosed informed consent document. The translated version should be dated and version controlled.

The bold sections of the enclosed informed consent document are the sections that must appear in the translation.

The translated informed consent form is part of the documents to be submitted to the ethics committee for approval. The competent ethics committee for each institution must validate local informed consent documents before the center can join the study. It is the responsibility of the Local Ethical Committee to guarantee that the translation is conforming to the ICH-GCP guidelines.

It will be emphasized that the participation is voluntary and that the patient is allowed to refuse further participation in the protocol whenever he/she wants. This will not prejudice the patient's subsequent care. Documented informed consent must be obtained for all patients included in the study before they are registered or randomized in the study. This must be done in accordance with the national and local regulatory requirements.

18.0 PUBLICATIONS OF DATA AND PROTECTION OF TRADE SECRETS

The Principal Investigator holds the primary responsibility for publication of the study results; provided that the Principal Investigator will provide Exelixis with a copy of any proposed publication or release: (a)

for abstracts, slide presentations or posters, at least five (5) business day prior to submission (in the case of abstracts) or first public presentation (in the case of slide presentations and posters); and (b) at least thirty (30) days in advance of first submission and each subsequent submission in the case of manuscripts and also comply with any provisions regarding publication that are agreed to between the Principal Investigator's institution (City of Hope National Medical Center.) and Exelixis, Inc. in the Clinical Trial Agreement related to this study.

19.0 REFERENCES

1. Cancer of the Kidney and Renal Pelvis - Cancer Stat Facts. SEER. Accessed November 16, 2020. https://seer.cancer.gov/statfacts/html/kidrp.html

2. Choueiri TK, Motzer RJ. Systemic Therapy for Metastatic Renal-Cell Carcinoma. *N Engl J Med*. 2017;376(4):354-366. doi:10.1056/NEJMra1601333

3. T.K. Choueiri, T. Powles. Nivolumab + cabozantinib vs sunitinib in first-line treatment for advanced renal cell carcinoma: First results from the randomized phase III CheckMate 9ER trial. *Annals of Oncology (2020)*. 2020;31(Suppl4). doi:10.1016/annonc/annonc325

4. Vetizou M, Pitt JM, Daillere R, et al. Anticancer immunotherapy by CTLA-4 blockade relies on the gut microbiota. *Science*. 2015;350(6264):1079-1084. doi:10.1126/science.aad1329

5. Sivan A, Corrales L, Hubert N, et al. Commensal Bifidobacterium promotes antitumor immunity and facilitates anti-PD-L1 efficacy. *Science*. 2015;350(6264):1084-1089. doi:10.1126/science.aac4255

6. Gopalakrishnan V, Spencer CN, Nezi L, et al. Gut microbiome modulates response to anti–PD-1 immunotherapy in melanoma patients. *Science*. 2018;359(6371):97-103. doi:10.1126/science.aan4236

7. Derosa L, Hellmann MD, Spaziano M, et al. Negative association of antibiotics on clinical activity of immune checkpoint inhibitors in patients with advanced renal cell and non-small-cell lung cancer. *Annals of Oncology*. 2018;29(6):1437-1444. doi:10.1093/annonc/mdy103

8. Bolger AM, Lohse M, Usadel B. Trimmomatic: a flexible trimmer for Illumina sequence data. *Bioinformatics*. 2014;30(15):2114-2120. doi:10.1093/bioinformatics/btu170

9. Segata N, Waldron L, Ballarini A, Narasimhan V, Jousson O, Huttenhower C. Metagenomic microbial community profiling using unique clade-specific marker genes. *Nat Methods*. 2012;9(8):811-814. doi:10.1038/nmeth.2066

10. Truong DT, Franzosa EA, Tickle TL, et al. MetaPhlAn2 for enhanced metagenomic taxonomic profiling. *Nat Methods*. 2015;12(10):902-903. doi:10.1038/nmeth.3589

11. Salgia NJ, Bergerot PG, Maia MC, et al. Stool Microbiome Profiling of Patients with Metastatic Renal Cell Carcinoma Receiving Anti–PD-1 Immune Checkpoint Inhibitors. *European Urology*. 2020;78(4):498-502. doi:10.1016/j.eururo.2020.07.011

12. Dizman N, Hsu J, Bergerot PG, et al. Randomized trial assessing impact of probiotic supplementation on gut microbiome and clinical outcome from targeted therapy in metastatic renal cell carcinoma. *Cancer Med*. Published online November 2020:cam4.3569. doi:10.1002/cam4.3569

13. Isa K, Oka K, Beauchamp N, et al. Safety assessment of the *Clostridium butyricum* MIYAIRI 588 ® probiotic strain including evaluation of antimicrobial sensitivity and presence of *Clostridium* toxin genes in vitro and teratogenicity in vivo. *Hum Exp Toxicol*. 2016;35(8):818-832. doi:10.1177/0960327115607372

14. Seki H, Shiohara M, Matsumura T, et al. Prevention of antibiotic-associated diarrhea in children by Clostridium butyricum MIYAIRI. *Pediatr Int*. 2003;45(1):86-90. doi:10.1046/j.1442-200X.2003.01671.x

15. Sato S, Nagai H, Igarashi Y. Effect of Probiotics on Serum Bile Acids in Patients with Ulcerative Colitis. *HGE*. Published online December 22, 2011. doi:10.5754/hge11789

16. Chalmin F, Ladoire S, Mignot G, et al. Membrane-associated Hsp72 from tumor-derived exosomes mediates STAT3-dependent immunosuppressive function of mouse and human myeloid-derived suppressor cells. *J Clin Invest*. Published online January 19, 2010:JCI40483. doi:10.1172/JCI40483

17. Phan DQ, Silka MJ, Lan Y-T, Chang R-KR. Comparison of Formulas for Calculation of the Corrected QT Interval in Infants and Young Children. *J Pediatr*. 2015;166(4):960-964.e2. doi:10.1016/j.jpeds.2014.12.037

18. Wright JR, Ung YC, Julian JA, et al. Randomized, double-blind, placebo-controlled trial of erythropoietin in non-small-cell lung cancer with disease-related anemia. *J Clin Oncol*. 2007;25(9):1027-1032. doi:10.1200/JCO.2006.07.1514

19. Lee DW, Santomasso BD, Locke FL, et al. ASTCT Consensus Grading for Cytokine Release Syndrome and Neurologic Toxicity Associated with Immune Effector Cells. *Biol Blood Marrow Transplant*. 2019;25(4):625-638. doi:10.1016/j.bbmt.2018.12.758

20. Rotz SJ, Leino D, Szabo S, Mangino JL, Turpin BK, Pressey JG. Severe cytokine release syndrome in a patient receiving PD-1-directed therapy. *Pediatr Blood Cancer*. 2017;64(12). doi:10.1002/pbc.26642

21. Adashek ML, Feldman M. Cytokine Release Syndrome Resulting From Anti-Programmed Death-1 Antibody: Raising Awareness Among Community Oncologists. *J Oncol Pract*. 2019;15(9):502-504. doi:10.1200/JOP.19.00160

22. Riegler LL, Jones GP, Lee DW. Current approaches in the grading and management of cytokine release syndrome after chimeric antigen receptor T-cell therapy. *Ther Clin Risk Manag*. 2019;15:323-335. doi:10.2147/TCRM.S150524

23. Michot JM, Bigenwald C, Champiat S, et al. Immune-related adverse events with immune checkpoint blockade: a comprehensive review. *Eur J Cancer*. 2016;54:139-148. doi:10.1016/j.ejca.2015.11.016

24. Zhu Q, Dupont CL, Jones MB, et al. Visualization-assisted binning of metagenome assemblies reveals potential new pathogenic profiles in idiopathic travelers' diarrhea. *Microbiome*. 2018;6(1):201. doi:10.1186/s40168-018-0579-0

25. Liu CM, Kachur S, Dwan MG, et al. FungiQuant: A broad-coverage fungal quantitative real-time PCR assay. *BMC Microbiol*. 2012;12(1):255. doi:10.1186/1471-2180-12-255

26. Taylor DL, Walters WA, Lennon NJ, et al. Accurate Estimation of Fungal Diversity and Abundance through Improved Lineage-Specific Primers Optimized for Illumina Amplicon Sequencing. Cullen D, ed. *Appl Environ Microbiol*. 2016;82(24):7217-7226. doi:10.1128/AEM.02576-16

27. Kozich JJ, Westcott SL, Baxter NT, Highlander SK, Schloss PD. Development of a Dual-Index Sequencing Strategy and Curation Pipeline for Analyzing Amplicon Sequence Data on the MiSeq Illumina Sequencing Platform. *Appl Environ Microbiol*. 2013;79(17):5112-5120. doi:10.1128/AEM.01043-13

28. Bolyen E, Rideout JR, Dillon MR, et al. Reproducible, interactive, scalable and extensible microbiome data science using QIIME 2. *Nat Biotechnol*. 2019;37(8):852-857. doi:10.1038/s41587-019-0209-9

29. Wen C, Zheng Z, Shao T, et al. Correction to: Quantitative metagenomics reveals unique gut microbiome biomarkers in ankylosing spondylitis. *Genome Biology*. 2017;18(1):214. doi:10.1186/s13059-017-1352-6

30. Yu J, Feng Q, Wong SH, et al. Metagenomic analysis of faecal microbiome as a tool towards targeted non-invasive biomarkers for colorectal cancer. *Gut*. 2017;66(1):70-78. doi:10.1136/gutjnl-2015-309800

31. Langmead B, Salzberg SL. Fast gapped-read alignment with Bowtie 2. *Nature Methods*. 2012;9(4):357-359. doi:10.1038/nmeth.1923

32. Franzosa EA, McIver LJ, Rahnavard G, et al. Species-level functional profiling of metagenomes and metatranscriptomes. *Nat Methods*. 2018;15(11):962-968. doi:10.1038/s41592-018-0176-y

33. Gibson MK, Forsberg KJ, Dantas G. Improved annotation of antibiotic resistance determinants reveals microbial resistomes cluster by ecology. *ISME J*. 2015;9(1):207-216. doi:10.1038/ismej.2014.106

34. Eisenhauer EA, Therasse P, Bogaerts J, et al. New response evaluation criteria in solid tumours: Revised RECIST guideline (version 1.1). *European Journal of Cancer*. 2009;45(2):228-247. doi:10.1016/j.ejca.2008.10.026

APPENDIX A: PERFORMANCE STATUS CONVERSION CHART

ECOG PERFORMANCE STATUS	KARNOFSKY PERFORMANCE STATUS
0—Fully active, able to carry on all pre-disease performance without restriction	100—Normal, no complaints; no evidence of disease<br><br>90—Able to carry on normal activity; minor signs or symptoms of disease
1—Restricted in physically strenuous activity but ambulatory and able to carry out work of a light or sedentary nature, e.g., light house work, office work	80—Normal activity with effort, some signs or symptoms of disease<br><br>70—Cares for self but unable to carry on normal activity or to do active work
2—Ambulatory and capable of all selfcare but unable to carry out any work activities; up and about more than 50% of waking hours	60—Requires occasional assistance but is able to care for most of personal needs<br><br>50—Requires considerable assistance and frequent medical care
3—Capable of only limited selfcare; confined to bed or chair more than 50% of waking hours	40—Disabled; requires special care and assistance<br><br>30—Severely disabled; hospitalization is indicated although death not imminent
4—Completely disabled; cannot carry on any selfcare; totally confined to bed or chair	20—Very ill; hospitalization and active supportive care necessary<br><br>10—Moribund
5—Dead	0—Dead

APPENDIX B: STOOL COLLECTION PROCEDURE

STOOL COLLECTION KIT GENERAL INSTRUCTIONS

As a part of your participation in the current study, we have some specific instructions related to collection of stool. Please abide by these instructions, as they are essential for the proper conduct of the study.

TOOL SAMPLING USING THE OMNIgene GUT Kit

Read all instructions prior to sample collection.

Samples should ONLY be collected Sunday-Wednesday and shipped Monday-Wednesday.

If you have diarrhea, wait until the next bowel movement to collect the sample.

Your collection kit will be composed of the following:

☐	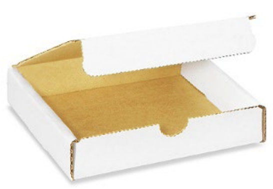	Shipment box – save to return the sample
☐	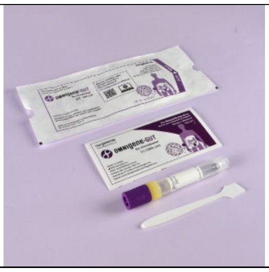	OMNIgene GUT Kit Collection Kit
☐	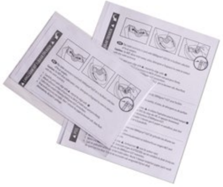	Toilet Accessory
☐	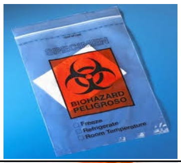	Plastic Biohazard Specimen Bag with Absorbent Paper
☐	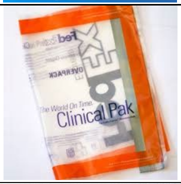	Plastic FedEx Clinical Pak
☐	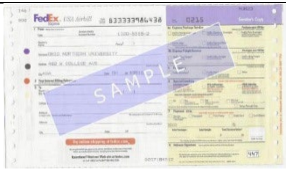	Preprinted FedEx Shipping Label

If any part of the kit is missing contact Luis Meza at 626-803-3354 immediately to obtain the missing materials.

Stool Collection Instructions:

1. Read the instructions provided in the OMNIgene□GUT Kit Collection Kit before beginning stool collection. You can watch the instructional video on YouTube.
2. Wash with water if the stabilizing liquid comes in contact with skin or eyes.
3. Wash your hands thoroughly.
4. Empty your bladder prior to collection.
5. Use the Toilet Accessory (Appendix A) to collect stool free from toilet water and urine. Follow the instructions provided with the Toilet Accessory. DO NOT use toilet paper for collection.
6. Collect your stool sample from the Toilet Accessory following the OMNIgene□GUT instructions (Appendix B) included with the kit. DO NOT push the stool into the tube. Only a small amount of sample is needed. Be sure that the purple cap is screwed on tightly.
7. Shake the tube vigorously for at least 30 seconds or until all of the solids have been dispersed into suspension
8. Place the tube in the Biohazard Specimen Bag and seal. Write the date and time of collection on the label on the bag.
9. Wash your hands thoroughly.
10. Place the Biohazard Specimen Bag in the Shipping Box.
11. Place the box inside the FedEx Clinical Pak. Affix the preprinted FedEx shipping label to the outside of the Clinical Pak.
12. Follow the Shipping Instructions on the following page. Return the sample within 24 hours of collection. Only ship Monday-Wednesday.

FedEx Shipment of Biological Samples

1. You may drop off your Clinical Pak at a staffed FedEx location (NOT a drop box). Remember that samples should remain at ambient temperature.

THANK YOU!

If you have any questions about any of these procedures do not hesitate to contact the study coordinator at (626) 803-3354 or microbiome@tgen.org.

OM-AC1 Toilet accessory

Used with OMNIgene•GUT

USER INSTRUCTIONS

This toilet accessory is used with OMNIgene®•GUT kit to facilitate collection of fecal samples.

Caution: Do NOT allow toilet water, urine, detergent or fragrance to come in contact with this toilet accessory.

Storage: 15°C to 25°C

Procedure:

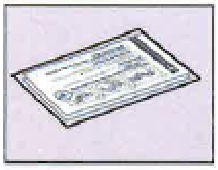

1 Two toilet accessories are provided in the event that you are unable to collect a sample on the first attempt.

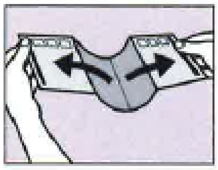

2 Carefully peel open edge with ▲; repeat for edge with ■.

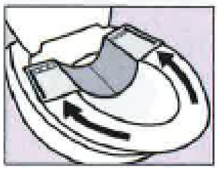

3 Attach adhesive surface of accessory to **BACK of toilet seat** with adhesive close to the outer edge of the seat and press firmly.

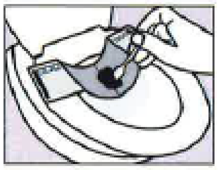

4 Collect fecal sample following OMNIgene•GUT user instructions.

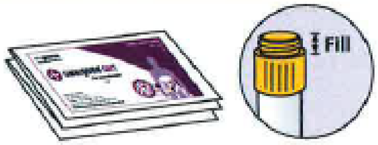

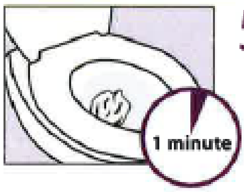

5 Drop used accessory into toilet. Wait 1 minute for paper to become soft, then flush. Alternatively, discard in garbage.

DNA genotek

Superior samples
Proven performance

OM-AC1 is made in Netherlands for DNA Genotek Inc.

3000 - 500 Palladium Drive
Ottawa, ON, Canada K2V 1C2

Toll-free (North America): 1.866.813.6354
Tel.: +1.613.723.5757 • Fax: +1.613.723.5057
info@dnagenotek.com
www.dnagenotek.com

®OMNIgene is a registered trademark of DNA Genotek Inc.
Some DNA Genotek products may not be available in all geographic regions, contact your sales representative for details.
All DNA Genotek protocols, white papers and application notes, are available in the support section of our website at www.dnagenotek.com.

Patent (www.dnagenotek.com/legalnotices)
© 2017 DNA Genotek Inc., a subsidiary of OraSure Technologies, Inc., all rights reserved.
PD-PR-00684 Issue 1/2017-05

CE

Version: 01

OMNIgene®·GUT

For microbiome

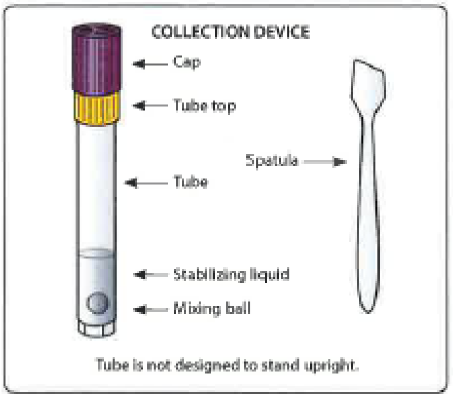

Summary and explanation of the kit:

OMNIgene·GUT provides the materials and instructions for collecting and stabilizing microbial DNA from a fecal sample.

Warnings and precautions:

- FOR EXTERNAL USE ONLY.
- Do NOT remove the yellow tube top from the tube.
- Do NOT spill the stabilizing liquid in the tube.
- Wash with water if liquid comes in contact with eyes or skin. Do NOT ingest.
- If collecting a liquid fecal sample, see separately provided user instructions.
- Small items may pose a choking hazard.

Storage: 15°C to 25°C

Ship in accordance to applicable regulations covering transport of biological specimens. See MSDS at www.dnagenotek.com

Label legend:

	Collect sample by (Use by)
REF	Catalog number
	Manufacturer
15°C – 25°C	Storage instructions
⚠	Caution, consult instructions for use
LOT	Lot number

USER INSTRUCTIONS

Read all instructions prior to collection

Procedure:

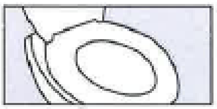

1 **IMPORTANT PREPARATIONS:**
- Empty your bladder before beginning the collection.
- Collect fecal sample free of urine or toilet water.
- Toilet paper or tissues may be required.

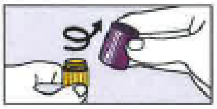

2 While holding the yellow tube top, unscrew ONLY the purple cap from the kit and set aside for later use.

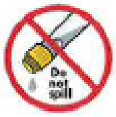

IMPORTANT:
Do NOT remove the yellow tube top.
Do NOT spill the stabilizing liquid in the tube.

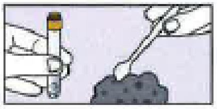

3 Use the spatula to collect a small amount of fecal sample.

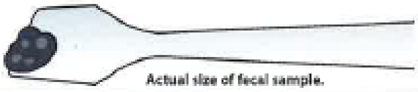

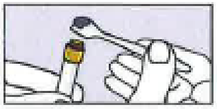

4 Transfer the fecal sample into the yellow tube top. Repeat until the sample fills the yellow tube top.

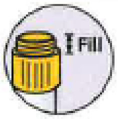

IMPORTANT: Do NOT push sample into the tube.

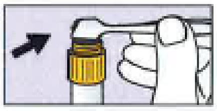

5 Scrape horizontally across the tube top to level the sample and remove any excess.

Wipe exterior of tube and top with toilet paper or tissue as needed.

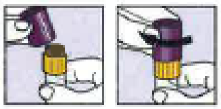

6 Pick up the purple cap with the solid end facing down and screw onto the yellow tube top until tightly closed.

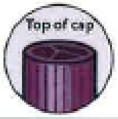

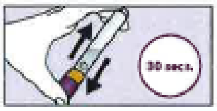

7 Shake the sealed tube as hard and fast as possible in a back and forth motion for a minimum of 30 seconds.

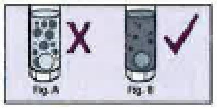

8 The fecal sample will be mixed with the stabilizing liquid in the tube; not all particles will dissolve.

IMPORTANT: Continue shaking if **large** particles remain as shown in Figure A.

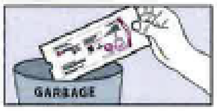

9 Place spatula in original packaging or wrap in toilet paper and discard in garbage.

IMPORTANT: Send the sample for processing following the delivery instructions supplied separately by the kit provider.

DNA genotek

Superior samples
Proven performance

Made in Canada
DNA Genotek Inc.
3000 - 500 Palladium Drive
Ottawa, ON, Canada K2V 1C2

Toll-free (North America): 1.866.813.6354
Tel.: +1.613.723.5757 • Fax: +1.613.723.5057
info@dnagenotek.com
www.dnagenotek.com

Australian Sponsor: Emergo Australia, Level 20, Tower II, Darling Park, 201 Sussex Street, Sydney, NSW 2000 Australia

OMNIgene-GUT (OM-200) is not available for sale in the United States.
OMNIgene-GUT (OMR-200) is for research use only, not for use in diagnostic procedures.
®OMNIgene is a registered trademark of DNA Genotek Inc.
Some DNA Genotek products may not be available in all geographic regions, contact your sales representative for details.
All DNA Genotek protocols, white papers and application notes, are available in the support section of our website at www.dnagenotek.com.

Patent (www.dnagenotek.com/legalnotices)
© 2018 DNA Genotek Inc., a subsidiary of OraSure Technologies, Inc., all rights reserved.
PD-PR-00442 Issue 5/2018-04

APPENDIX C: STUDY LOG

DIET and STOOL FREQUENCY LOG - GENERAL INSTRUCTIONS

As a part of your participation in the current study, we are requesting that you complete a study log every day.

General pointers:

- When you come to the clinic, bring your logs with you.
- Each page has room for seven days – one row should be completed for each day.
- Please make specific note of intake of any yogurt, yogurt-containing foods, or other probiotics

Example of how the top part of the log will look:

- A study team member will complete the information in this box before you leave the clinic.

COMPLETED BY STUDY TEAM	Participant Initials: JSM	Participant Research Number: 1001	Group: A

Example of how the information you enter might look:

- You or someone close to can complete the log for you, so long as the information is correct.
- List all prescription and non-prescription medications.
- The person who completes that day's entry should write his or her initials in the last column.

Day and Date	General description of food I ate:	*Did I eat yogurt or take probiotics?*	*How was my stool frequency?*	*Was a stool sample collected?*	*Medications taken*	*Initials of person filling information*
	Eggs, toast, juice<br>Ham sandwich, coke, potato chips<br>Steak, mashed potatoes, wine	○ Yes<br>☒ No	☒ Seems like baseline<br>○ 1-3 stools more than baseline<br>○ 4-6 stools more than normal<br>○ 7 or more than baseline, or incontinence	○ Yes<br>☒ No	Vitamin C, Lipitor	JBS
			○ Seems like baseline			

Example of the signature line:

- When you hand over the document to the study team, they will ask to sign and date at the bottom of each log if you agree that the information is complete and correct.

At the time of handing over the document -- Participant Signature: Joseph Black Smith Date 12/18/2002

COMPLETED BY STUDY TEAM Participant Initials: Participant Research Number: Group:

Day and Dat e	General description of food I ate:	*Did I eat yogurt or take probiotics?*	*How was my stool frequency?*	*Was a stool sample collected?*	*Medications taken*	*Initials of person filling information*
	**Eggs, toast, juice**<br>**Ham sandwich, coke, potato chips**<br>**Steak, m ashed potatoes, wine**	◯ Yes<br>◯ No X	X ◯Seems like baseline<br>◯1-3 stools more than baseline<br>◯4-6 stools more than normal<br>◯7 or more than baseline, or incontinence	◯ Yes<br>◯ No X	**Vitamin C, lipitor**	*JBC*
		◯ Yes<br>◯ No	◯ Seems like baseline<br>◯1-3 stools more than baseline<br>◯4-6 stools more than normal<br>◯7 or more than baseline, or incontinence	◯ Yes<br>◯ No		
		◯ Yes<br>◯ No	◯ Seems like baseline<br>◯1-3 stools more than baseline<br>◯4-6 stools more than normal<br>◯7 or more than baseline, or incontinence	◯ Yes<br>◯ No		
		◯ Yes<br>◯ No	◯ Seems like baseline<br>◯1-3 stools more than baseline<br>◯4-6 stools more than normal<br>◯7 or more than baseline, or incontinence	◯ Yes<br>◯ No		
		◯ Yes<br>◯ No	◯ Seems like baseline<br>◯1-3 stools more than baseline<br>◯4-6 stools more than normal<br>◯7 or more than baseline, or incontinence	◯ Yes<br>◯ No		
		◯ Yes<br>◯ No	◯ Seems like baseline<br>◯1-3 stools more than baseline<br>◯4-6 stools more than normal	◯ Yes<br>◯ No		

			◯7 or more than baseline, or incontinence			
		◯ Yes<br>◯ No	◯ Seems like baseline<br>◯1-3 stools more than baseline<br>◯4-6 stools more than normal<br>◯7 or more than baseline, or incontinence	◯ Yes<br>◯ No		

STUDY DRUG MEDICATION DIARY

Please record how many packets you take of CBM588 and tablets of Cabozantinib, the time you take them and any comments here below and bring the completed Diary as well as your study drug supply, including empty bottles, to every study visit. This will help us keep track of your study drug and how well you are tolerating it. If a drug is vomited, dosing for the day should be skipped (do not take another tablet/satchel in its place) and missed dose should be recorded in the study medication diary.

STUDY DRUG INSTRUCTIONS:

Study Drug: CMB588
How Much: Your dose is 80 mg
How Often: You will take each dose twice daily
When: You should take your dose once in the morning and once in the evening

		Cycle #: _______________<br>For each morning dose, take 2 packets of **CBM-588**<br>For each evening dose, take 2 packets of **CBM-588**				For each day, take 1 tablet of **Cabozantinib 40mg**		
		**CBM588-Morning Dose**		**CBM588-Evening Dose**		**Cabozantinib-Daily Dose**		
	**Date**	**Time Taken**	**# of Packets Taken**	**Time Taken**	**# of Packets Taken**	**Time Taken**	**# of Tablets Taken**	**Comments**
EXAMPLE:	2/1/2021	9:00 AM	2	8:00 PM	2	12:00 PM	1	Vomited Cabozantinib tablet
Day 1								
Day 2								
Day 3								
Day 4								
Day 5								
Day 6								
Day 7								
Day 8								
Day 9								

Day 10								
Day 11								
Day 12								
Day 13								
Day 14								
Day 15								
Day 16								
Day 17								
Day 18								
Day 19								
Day 20								
Day 21								
Day 22								
Day 23								
Day 24								
Day 25								
Day 26								
Day 27								
Day 28								

At the time of handing over the document -- Participant Signature: ____ Date ____________________________

FOR STUDY TEAM USE ONLY<br>Staff Initials:	
Date Dispensed:	Date Returned:
# pills/caps/tabs dispensed:	# pills/caps/tabs returned:
# pills/caps/tabs that should have been taken:	
Discrepancy Notes:	

DATA ACCESS AGREEMENT

The Translational Genomics Research Institute (TGen) has research data hosted in an established central data repository.

Access to human genomic data will be provided to research investigators who, along with their institutions, have certified their agreement with the expectations and terms of access detailed below. It is the intent of TGen that approved users of TGen datasets recognize all restrictions on data use established by TGen.

The parties to this agreement include: the Principal Investigator (PI) requesting access to the genomic study dataset (the "Approved User"), his/her home institution as represented by the Institutional Signing Official (the "User Institution"), and TGen (the "Data Producers"). The effective date of this agreement shall be the later of a) the Project Approval Date, as specified on the Data Access Committee (DAC) approval notification, and b) the date of the last signature on this Agreement.

These terms and conditions govern access to the managed access datasets (details of which are set out in Appendix I) to which the Approved User and User Institution has requested access. The Approved User and User Institution agree to be bound by these terms and conditions.

DEFINITIONS

Approved User: The Principal Investigator for the Project.

Authorized Personnel*:* The individuals at the User Institution to whom TGen grants access to the Data. This includes the Approved User, the individuals listed in Appendix II and any other individuals for whom the User

Institution subsequently requests access to the Data (in a subsequent, separate request approved by TGen). Details of the initial Authorized Personnel are set out in Appendix II.

***Data**:* The managed access datasets to which the User Institution has requested access.

***Data Producers**:* TGen and the collaborators listed in Appendix I responsible for the development, organization, and oversight of these Data.

***External Collaborator**:* A collaborator of the Approved User, working for an institution other than the User Institution.

***Project**:* The project for which the User Institution has requested access to these Data. A description of the Project is set out in Appendix II.

***Publications**:* Includes, without limitation, articles published in print journals, electronic journals, reviews, books, posters and other written and verbal presentations of research.

***Research Participant**:* An individual whose data form part of the requested Data.

Research Purposes: Shall mean research that is seeking to advance the understanding of genetics and genomics, including the treatment of disorders, and work on statistical methods that may be applied to such research.

***User Institution(s)**:* The User Institution that has requested access to the Data.

TERMS OF ACCESS

1. The User Institution agrees to only use these Data for the purpose of the Project (described in Appendix II) and only for Research Purposes. The User Institution further agrees that it will only use these Data for Research Purposes, which are within the limitations (if any) set out in Appendix I.

2. The User Institution agrees to preserve, at all times, the confidentiality of these Data. In particular, it undertakes not to use, or attempt to use these Data to compromise or otherwise breach the obligations of confidentiality of information on Research Participants. Without prejudice to the generality of the foregoing, the User Institution agrees to use at least the measures set out in Appendix I to protect these Data.

3. The User Institution agrees to protect the confidentiality of Research Participants in any research papers or publications that they prepare by taking all reasonable care to limit the possibility of identification.

4. The User Institution agrees not to link or combine these Data to other information or archived data available in a way that could re-identify the Research Participants, even if access to that data has been formally granted to the User Institution or is freely available without restriction.

5. The User Institution agrees only to transfer or disclose these Data, in whole or part, or any material derived from these Data, to the Authorized Personnel. Should the User Institution wish to share these Data with an External Collaborator, the External Collaborator must complete a separate application for access to these Data.

6. The User Institution agrees that the Data Producers, and all other parties involved in the creation, funding or protection of these Data: a) make no warranty or representation, express or implied as to the accuracy, quality or comprehensiveness of these Data; b) exclude to the fullest extent permitted by law all liability for actions, claims, proceedings, demands, losses (including but not limited to loss of profit), costs, awards damages and payments made by the User Institution that may arise (whether directly or indirectly) in any way whatsoever from the User Institution’s use of these Data or from the unavailability of, or break

in access to, these Data for whatever reason and; c) bear no responsibility for the further analysis or interpretation of these Data.

7. The User Institution agrees to follow the *Fort Lauderdale Guidelines* and the *Toronto Statement*. This includes but is not limited to recognizing the contribution of the Data Producers and including a proper acknowledgement in all reports or publications resulting from the use of these Data.

8. The User Institution agrees to follow the *Publication Policy* in Appendix III. This includes respecting the moratorium period for the Data Producers to publish the first peer-reviewed report describing and analyzing these Data.

9. The User Institution agrees not to make intellectual property claims on these Data and not to use intellectual property protection in ways that would prevent or block access to, or use of, any element of these Data, or conclusion drawn directly from these Data.

10. The User Institution can elect to perform further research that would add intellectual and resource capital to these data and decide to obtain intellectual property rights on these downstream discoveries. In this case, the User Institution agrees to implement licensing policies that will not obstruct further research and to follow the U.S. National Institutes of Health *Best Practices for the Licensing of Genomic Inventions (2005)* in conformity with the Organization for Economic Co-operation and Development *Guidelines for the Licensing of the Genetic Inventions (2006)*.

11. The User Institution agrees to destroy/discard the Data held, once it is no longer used for the Project, unless obliged to retain the Data for archival purposes in conformity with audit or legal requirements.

12. The User Institution will notify TGen in writing within 30 days of any changes or departures of Authorized Personnel.

13. The User Institution will notify TGen in writing prior to any significant changes to the protocol for the Project.

14. The User Institution will notify TGen in writing as soon as it becomes aware of a breach of the terms or conditions of this agreement.

15. TGen or the User Institution may terminate this agreement at any time, and for any reason, by written notice to the other party. If this agreement is terminated, the User Institution shall destroy any Data held, including copies and backup copies. This clause does not prevent the User Institution from retaining these Data for archival purpose in conformity with audit or legal requirements.

16. The User Institution accepts that it may be necessary for the Data Producers to modify the terms of this agreement from time to time. As an example, such modifications may include specific provisions relating to the Data required by Data Producers other than TGen. In the event that changes are required, the Data Producers or their appointed agent will contact the User Institution to inform it of the changes, and the User Institution may elect to accept the changes or terminate the agreement.

17. If requested, the User Institution will allow data security and management documentation to be inspected by TGen to verify that it is complying with the terms of this agreement.

18. The User Institution agrees to distribute a copy of these terms to all Authorized Personnel and require the Authorized Personnel to comply with the terms of this agreement.

19. This agreement (and any dispute, controversy, proceedings or claim of whatever nature arising out of this agreement or its formation) shall be construed, interpreted and governed by the laws of the state of New York, without reference to its conflicts of law provisions.

Agreed for User Institution (Signatory Official)

**Signature:**	
**Name:**	
**Title:**	
**Date:**	

Principal Investigator

I confirm that I have read and understood this Agreement.

**Signature:**	
**Name:**	
**Title:**	
**Date:**	

Agreed for TGen

**Signature:**	
**Name:**	**Stephanie Buchholtz, PhD**
**Title:**	**Sr. Director, Office of Research Compliance & Quality Mgmt**
**Date:**	

APPENDIX I – DATASET DETAILS
APPENDIX II —PROJECT DETAILS
APPENDIX III — PUBLICATION POLICY

APPENDIX I – DATASET DETAILS

Dataset reference (TGen Study ID and Dataset Details)

[insert name of data set] Dataset maintained by the Translational Genomics Research Institute.

Name of project that created the dataset

[insert study title]

Names of other data producers/collaborators

[insert collaborators]

Specific limitations on areas of research

[insert limitations such as consent limitations or "No specific limitations set"]

Minimum protection measures required

The Approved User, their User Institution and all Authorized Users agree to handle the data according to the current *NIH Security Best Practices for Controlled-Access Data Subject to the NIH Genomic Data Sharing (GDS) Policy* *(09MAR2015)*.

File access

Data can be held in unencrypted files on an institutional computer system, with Unix user group read/write access for one or more appropriate groups but not Unix world read/write access behind a secure firewall. Laptops holding these data should have password protected logins and screen locks (set to lock after 5 min of inactivity). If held on USB keys or other portable hard drives, the data must be encrypted.

APPENDIX II – PROJECT DETAILS (to be completed by the Approved User)

Details of dataset requested i.e., EGA Study and Dataset Accession Number

[insert Data set name or ID #] Dataset maintained by the Translational Genomics Research Institute.

Name of Principal Investigator	***Email***	***Job Title***	***User Institution***

Brief abstract of the Project in which the Data will be used (500 words max)

All Individuals who the User Institution requests to be named as Authorized Personnel (add rows as needed)

Name of Authorized Personnel	***Email***	***Job Title***	***Supervisor****

All Individuals that should have an account created at the EGA

**Name of Authorized User**	**Email**	**Job Title**

APPENDIX III – PUBLICATION POLICY

In any publications based on these Data, please describe how the Data can be accessed, including the name of the data set and its accession numbers, and acknowledge its use in a form agreed by the User Institution with TGen.
