# Supplementary material for: Cabozantinib and nivolumab with or without live bacterial supplementation in metastatic renal cell carcinoma: a randomized phase 1 trial
Source: Nat Med. 2024 Jun 28;30(9):2576–85. doi: 10.1038/s41591-024-03086-4 (PMC11405272; doi:10.1038/s41591-024-03086-4)
Supplement: Supplementary file 2 — Reporting Summary [file 41591_2024_3086_MOESM2_ESM.pdf]

nature portfolio

Corresponding author(s): Sumanta K Pal, MD
Abhishek Tripathi, MD

Last updated by author(s): May 17, 2024

Reporting Summary

Nature Portfolio wishes to improve the reproducibility of the work that we publish. This form provides structure for consistency and transparency in reporting. For further information on Nature Portfolio policies, see our Editorial Policies and the Editorial Policy Checklist.

Statistics

For all statistical analyses, confirm that the following items are present in the figure legend, table legend, main text, or Methods section.

n/a	Confirmed	
☐	☒	The exact sample size (*n*) for each experimental group/condition, given as a discrete number and unit of measurement
☐	☒	A statement on whether measurements were taken from distinct samples or whether the same sample was measured repeatedly
☐	☒	The statistical test(s) used AND whether they are one- or two-sided<br>*Only common tests should be described solely by name; describe more complex techniques in the Methods section.*
☐	☒	A description of all covariates tested
☒	☐	A description of any assumptions or corrections, such as tests of normality and adjustment for multiple comparisons
☐	☒	A full description of the statistical parameters including central tendency (e.g. means) or other basic estimates (e.g. regression coefficient) AND variation (e.g. standard deviation) or associated estimates of uncertainty (e.g. confidence intervals)
☐	☒	For null hypothesis testing, the test statistic (e.g. *F*, *t*, *r*) with confidence intervals, effect sizes, degrees of freedom and *P* value noted<br>*Give P values as exact values whenever suitable.*
☒	☐	For Bayesian analysis, information on the choice of priors and Markov chain Monte Carlo settings
☒	☐	For hierarchical and complex designs, identification of the appropriate level for tests and full reporting of outcomes
☒	☐	Estimates of effect sizes (e.g. Cohen's *d*, Pearson's *r*), indicating how they were calculated

Our web collection on statistics for biologists contains articles on many of the points above.

Software and code

Policy information about availability of computer code

Data collection	FLEXMAP 3D system (Luminex); Cytek Aurora spectrum cytometer (5 laser configuration), KingFisher Apex System (ThermoFisher Scientific), NovaSeq X (Illumina)
Data analysis	R software version 4.3.0, GraphPad Prism 10.0.0, BowTie2, Trimmomatic 0.33, MetaPhlAn 4.0, and HUMAnN3, FlowJo 10.7.1

For manuscripts utilizing custom algorithms or software that are central to the research but not yet described in published literature, software must be made available to editors and reviewers. We strongly encourage code deposition in a community repository (e.g. GitHub). See the Nature Portfolio guidelines for submitting code & software for further information.

Data

Policy information about availability of data

All manuscripts must include a data availability statement. This statement should provide the following information, where applicable:

- Accession codes, unique identifiers, or web links for publicly available datasets
- A description of any restrictions on data availability
- For clinical datasets or third party data, please ensure that the statement adheres to our policy

Human genome GRCh38.p7 was accessed trough https://www.ncbi.nlm.nih.gov/datasets/genome/GCF_000001405.33/ (NCBI RefSeq assembly number: GCF_000001405.33). Metagenomic data from stool sufficient to replicate the analyses presented herein will be deposited in Translational Genomics Research Institute (TGen) and will be available upon request. Authors defer depositing the participant genomic data in national and international public repositories due to

institutional policies, and the absence of statements in patient consent forms which would have allowed controlled access distribution and genomic data availability. De-identified individual participant whole metagenome libraries and clinical data that underlie the results reported in this article are available for transfer on a specific secure server housed at TGen. Interested investigators can obtain and certify the data transfer agreement (DTA) and submit requests to the principal investigator, Abhishek Tripathi, MD (spal@coh.org). Proposals will be vetted by the TGen Data Access Committee. Investigators/institutions who consent to the terms of the DTA form, including but not limited to the use of these data for the purpose of a specific project and only for research purposes, protect the confidentiality of the data and limit the possibility of identification of participants in any way whatsoever for the duration of the agreement will be granted access. TGen will then facilitate the transfer of the requested de-identified data. This mechanism is expected to be via an Aspera High Speed File Transfer Server at the time of this publication, but TGen reserves the right to change the specific transfer method at any time, provided appropriate levels of access authorization and control can be maintained.

Research involving human participants, their data, or biological material

Policy information about studies with human participants or human data. See also policy information about sex, gender (identity/presentation), and sexual orientation and race, ethnicity and racism.

Reporting on sex and gender

The findings of this study apply do not apply exclusively to one sex/gender. Patient sex was considered as a demographic variable in the reporting of our results and was determined based on assigned sex as noted in the patients' electronic medical records. No sex- or gender-based analyses were performed in this study as there is currently no clinical or pre-clinical evidence suggesting that there are differential outcomes based on these variables .

Reporting on race, ethnicity, or other socially relevant groupings

Self reported data for race and ethnicity were included when reporting demographic characteristics of our cohort. However, these were not use for subgroup analysis of our results. Permuted block randomization was used to help diminish any potentially confounding variables for our study endpoints

Population characteristics

Patients included in this study had locally advanced unresectable or metastatic RCC with clear-cell, papillary or sarcomatoid component and presence of measurable disease per Response Evaluation Criteria in Solid Tumors (RECIST 1.1). They had to be age 18 years or older and have a Karnofsky performance status ≥70%. No prior systemic therapy for metastatic disease was allowed. A total of 30 patients with advanced or metastatic RCC were randomized and treated between November 3, 2021, and March 6 ,2023. Baseline characteristics were comparable between arms. The median age in the overall cohorts at the time of treatment initiation was 65 (range, 36-84 years). The majority of patients were male (67%) and had intermediate or poor risk disease (60%) as defined by the International Metastatic RCC Database Consortium (IMDC). While clear-cell RCC comprised the majority of patients (87%), 5 patients (17%) had sarcomatoid features/dedifferentiation and 2 patients had papillary RCC. The most common sites of metastases at the time of enrollment were lung (80%), lymph nodes (50%), and bones (40%).

Recruitment

Participants were identified and approached during routine clinical visits at City of Hope through participating clinicians. Patients were supplied with written informed consent and screened for eligibility. If inclusion criteria were met and no exclusion criteria were identified, they would start protocol-based treatment. We do not anticipate a bias in recruiting participants in this study. Patients were not compensated for their participation in this study.

Ethics oversight

The study (NCT05122546) was approved by the US Food and Drug Administration and by the City of Hope Institutional Review Board. Patients were required to supply written informed consent prior to participating. All study procedures were undertaken in accordance with the Declaration of Helsinki.

Note that full information on the approval of the study protocol must also be provided in the manuscript.

Field-specific reporting

Please select the one below that is the best fit for your research. If you are not sure, read the appropriate sections before making your selection.

☒ Life sciences ☐ Behavioural & social sciences ☐ Ecological, evolutionary & environmental sciences

For a reference copy of the document with all sections, see nature.com/documents/nr-reporting-summary-flat.pdf

Life sciences study design

All studies must disclose on these points even when the disclosure is negative.

Sample size

With a cumulative sample size of 30 patients (randomized in a 2:1 fashion), we would have 80% power to detect a one standard deviation change in specific Bifidobacterium spp. between study arms using a Mann-Whitney U-test with a one-sided type I error of 0.05.

Data exclusions

Clinical response and microbiome data for one patient originally randomized to the cabozantinib/nivolumab plus CBM588 arm was not included. The patient relocated to another state and stopped any protocol driven assessments (response, stool microbiome analysis) prior to week 13.

Replication

This study was limited to a single site, and therefore, assessing replicability is beyond the scope of this study. Statistical tests were employed to ensure the significance of the results within our study.

Randomization

To generate the random allocation sequence, permutation within a block was conducted using the "sample" function in R, without replacement, with a set seed documented. A fixed block size of 6 was used. The study statistician supplied the randomization log to the City of Hope central Data Coordinating Center (DCC). This file is kept in a secure computer folder within the DCC and not shared with anyone outside the DCC. The block size and method chosen were not shared with the clinical team by the statistician or DCC, nor was the allocation sequence.

	The study statistician generated the allocation sequence, and the DCC staff conducted the actual assignment. Neither the study statistician nor the DCC staff have any contact with the participants. Access to the randomization table is restricted to the DCC and lead statistician only.
Blinding	Investigators were not blinded to group allocation during data collection as the protocol demanded the use of dietary and medication logs and was not placebo controlled. However, analysis of the samples collected was performed in a blinded fashion.

Reporting for specific materials, systems and methods

We require information from authors about some types of materials, experimental systems and methods used in many studies. Here, indicate whether each material, system or method listed is relevant to your study. If you are not sure if a list item applies to your research, read the appropriate section before selecting a response.

Materials & experimental systems

n/a	Involved in the study
☒	☐ Antibodies
☒	☐ Eukaryotic cell lines
☒	☐ Palaeontology and archaeology
☒	☐ Animals and other organisms
☐	☒ Clinical data
☒	☐ Dual use research of concern
☒	☐ Plants

Methods

n/a	Involved in the study
☒	☐ ChIP-seq
☐	☒ Flow cytometry
☒	☐ MRI-based neuroimaging

Clinical data

Policy information about clinical studies
All manuscripts should comply with the ICMJE guidelines for publication of clinical research and a completed CONSORT checklist must be included with all submissions.

Clinical trial registration	The study (NCT05122546) was approved by the US Food and Drug Administration.
Study protocol	The full trial protocol is available as part of the supplemental material.
Data collection	All data was collected at the City of Hope Comprehensive Cancer Center in Duarte California.<br>Recruitment: 30 patients were recruited randomized from Nov 1, 2021 to March 2, 2023.<br>Data Collection: Collection from patient related data was performed from Nov 3, 2021 to August 16, 2023.
Outcomes	Primary Endpoint:<br>- Change in Bifidobacterium composition of stool from baseline to week 12 of therapy on the CBM588 with cabozantinib/nivolumab vs cabozantinib/nivolumab alone, using the Wilcoxon signed rank test to compare two timepoints within the same treatment arm.<br><br>Secondary Endpoints:<br>- Comparison of the Shannon index (a measure of microbial diversity) from baseline to week 12 of therapy on the CBM588+ cabozantinib/nivolumab vs cabozantinib/nivolumab alone.<br>- Best overall response, by RECIST criteria, with cabozantinib/nivolumab alone vs cabozantinib/nivolumab with CBM588.<br>- Progression-free survival (PFS), assessed as the duration of time from enrollment to progression, with cabozantinib/nivolumab alone vs cabozantinib/nivolumab with CBM588, estimated using the Kaplan-Meier method and compared between treatment arms using the Cox Proportional Hazards model.<br>- Comparison of the proportion of circulating Tregs at baseline to levels of circulating Tregs with cabozantinib/nivolumab alone vs cabozantinib/nivolumab with CBM588, using flow cytometry analysis of immune cells.<br>- Comparison of the proportion of circulating MDSCs with cabozantinib/nivolumab alone versus cabozantinib/nivolumab with CBM588, using flow cytometry analysis of immune cells.<br>- Comparison of IL-6, IL-8 and other cytokines/chemokines with cabozantinib/nivolumab alone versus cabozantinib/nivolumab with CBM588, using the Luminex Flexmap 3D system.<br>- Comparison of toxicities such as diarrhea and nausea using CTCAE v5 criteria with cabozantinib/nivolumab alone versus cabozantinib/nivolumab with CBM588.

Plants

Seed stocks	*Report on the source of all seed stocks or other plant material used. If applicable, state the seed stock centre and catalogue number. If plant specimens were collected from the field, describe the collection location, date and sampling procedures.*
Novel plant genotypes	*Describe the methods by which all novel plant genotypes were produced. This includes those generated by transgenic approaches, gene editing, chemical/radiation-based mutagenesis and hybridization. For transgenic lines, describe the transformation method, the number of independent lines analyzed and the generation upon which experiments were performed. For gene-edited lines, describe the editor used, the endogenous sequence targeted for editing, the targeting guide RNA sequence (if applicable) and how the editor was applied.*
Authentication	*Describe any authentication procedures for each seed stock used or novel genotype generated. Describe any experiments used to assess the effect of a mutation and, where applicable, how potential secondary effects (e.g. second site T-DNA insertions, mosiacism, off-target gene editing) were examined.*

Flow Cytometry

Plots

Confirm that:

☒ The axis labels state the marker and fluorochrome used (e.g. CD4-FITC).

☒ The axis scales are clearly visible. Include numbers along axes only for bottom left plot of group (a 'group' is an analysis of identical markers).

☒ All plots are contour plots with outliers or pseudocolor plots.

☒ A numerical value for number of cells or percentage (with statistics) is provided.

Methodology

Sample preparation	Peripheral blood samples were collected in 10 mL cell preparation tubes (BD Biosciences, San Jose, California, USA) at baseline and weeks 7, 12, 17 and 25. All samples were processed within a window of 4-6 hours upon collection. Processing involved centrifugation at 1800 x g for 20 minutes followed by plasma extraction for circulating cytokine analysis. After plasma extraction, the remaining cell suspension was transferred to conical propylene tubes, washed in cRPMI and recentrifuged at 250 x g for seven minutes at room temperature for isolation of peripheral blood mononuclear cells (PBMC). PBMCs were then immersed in a mixture of phosphate buffered saline, fetal calf serum and sodium azide with Fc III/IIR-specific antibody (commercially available Fc III/IIR-specific antibodies that have been validated by Biolegend, Invitrogen and BD) to block nonspecific binding and stained the cells with viability dye-Zombie NIR (Cat# 423106) and different combinations of fluorochrome labelled antibodies to CD3-BUV496 (Cat# 612940), CD4-PeCy7 (Cat# 25004942), CD8-BUV805 (Cat# 612889) and intracellular FoxP3-PE (Cat# 560852) (all sourced from either Biolegend, Invitrogen or BD Biosciences San Jose, California, USA). Flow cytometry data was collected using Cytek Aurora (Cytek, Freemont, CA, USA) and analyzed using FlowJo software version 10.7.1 (Beckton Dickinson, Franklin Lakes, New Jersey, USA).
Instrument	Cytek Aurora spectral cytometer (5 laser configuration)
Software	Flow cytometry data was analyzed with Flowjo version 10.7.1
Cell population abundance	Viable cells in our analysis were approximately 90% of all single cells. CD3+ T cells were approximately 77% of viable cells and CD4+ T cells were around 70% of CD3+ T cells and CD8+ T cells were 22% of CD3+ T cells. Foxp3+ T regulatory cells were around 3% of CD4+ T cells
Gating strategy	The gating strategy to identify immune cells populations are as follows: The starting cell population were identified using FSC/SSC gates, then single cells were gated using FSC-H/FSC-A. Viable cells were then gated as SSC-A/Zombie NIR negative cells before gating on SSC-A/CD3+ populations as the initial CD3+ T cell population. Following the identification of CD3+ T cells, CD4+ and CD8+ T cells were then gated as CD4+ PeCy7 cells or CD8+ BUV805+ cells respectively. For CD4+ FoxP3+ T regulatory cells, cells were identified as CD3+ and CD4+ T cells before gating on SSC-A/Foxp3+ T cells. The gates for FoxP3+ T cells were based on fluorescence minus one (FMO) controls as shown in the supplementary data.

☒ Tick this box to confirm that a figure exemplifying the gating strategy is provided in the Supplementary Information.
